# Prebiotic synthesis of dihydrouridine by photoreduction of uridine in formamide†

**DOI:** 10.1039/d4cc01823k

**Published:** 2024-06-10

**Authors:** Jianfeng Xu, Mikołaj J. Janicki, Rafał Szabla, John D. Sutherland

**Affiliations:** a MRC Laboratory of Molecular Biology Francis Crick Avenue, Cambridge Biomedical Campus Cambridge CB2 0QH UK jxu@mrc-lmb.cam.ac.uk; b Department of Physical and Quantum Chemistry, Faculty of Chemistry, Wrocław University of Science and Technology Wybrzeże Wyspiańskiego 27 50-370 Wrocław Poland mikolaj.janicki@pwr.edu.pl; c Institute of Advanced Materials, Faculty of Chemistry, Wrocław University of Science and Technology Wybrzeże Wyspiańskiego 27 50-370 Wrocław Poland

## Abstract

In this report, we show that a very common modification (especially in tRNA), dihydrouridine, was efficiently produced by photoreduction of the canonical pyrimidine ribonucleoside, uridine in formamide. Formamide not only acts as a solvent in this reaction, but also as the reductant. The other three components of the canonical alphabet (C, A, G) remained intact under the same conditions, suggesting that dihydrouridine might have coexisted with all four canonical RNA nucleosides (C, U, A, G) at the dawn of life.

## Abstract 2

UV irradiation of mixtures of canonical ribonucleosides (C, U, A, G) leads to a mixture of dihydrouridine (DHU, modified nucleoside in tRNA) with all four RNA canonical nucleosides (C, U, A, G).

## Graphical abstract

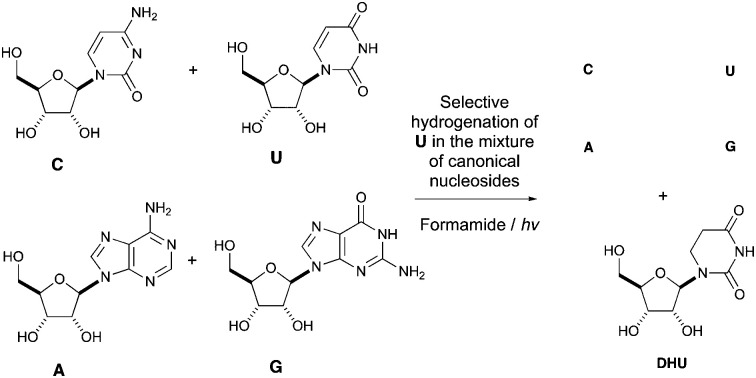

More than 160 modifications have been identified in biological RNA.^1^ The majority of modified nucleosides are present in transfer and ribosomal RNA across all cell types and organisms. tRNA, in particular, contains the highest frequency of modification to enhance its structural stabilization and modulate translation.^2^ Although most nucleobase modifications are believed to be post-transcriptionally incorporated by enzymes,^2^ and thus less prebiotically relevant, our recent studies in the prebiotic synthesis of canonical RNA or DNA nucleosides have suggested that non-canonical nucleosides like inosine, 2-thiocytidine and 2-thiouridine can be efficiently generated in the same geochemical scenario as the canonical nucleosides.^3,4^

It has been proposed that an emerging RNA world may have included many types of nucleobases, but selection pressures from chemical and early biological evolution could have narrowed the composition to that of extant RNA.^5^ UV irradiation has been considered as one of the major chemical selection pressures available on early Earth.^6–10^ For instance, it is well documented that canonical pyrimidine ribonucleosides, cytidine 1 and uridine 2, can reversibly form their photohydrates^11–13^3 and 4 in dilute aqueous solution under UV irradiation, and cytidine 1 can be photochemically converted to uridine 2 in the process (Fig. 1).^14–16^ However, no study in the literature has investigated the photochemical behavior of these two pyrimidine ribonucleosides in formamide 5 – a high boiling point solvent accessible and accumulable under early Earth conditions by the reaction of HCN and H_2_O.^17^

**Fig. 1:**
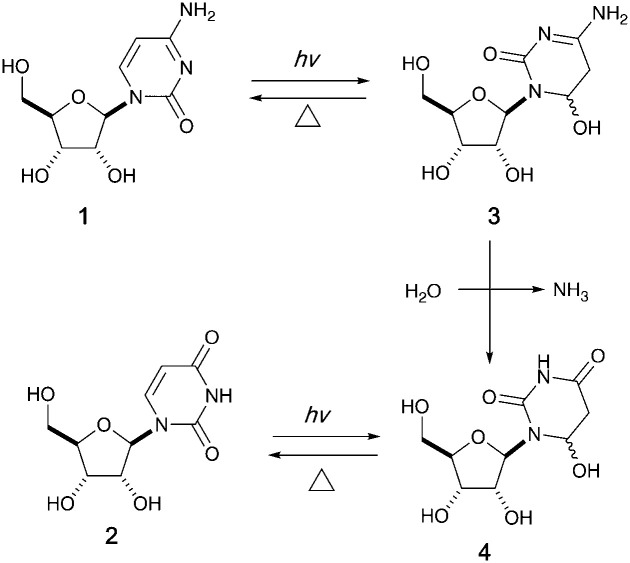
Photohydration of uridine and cytidine in H_2_O.^14^

Intrigued by this gap in the literature, we irradiated dilute solutions (16 mM) of uridine 2 or cytidine 1 in formamide. Surprisingly, we found that uridine was nearly quantitatively (94% yield) converted to 5,6-dihydrouridine (DHU, 6), an important modification found in transfer RNA which allows for conformational flexibility and dynamic motion in RNA structures,^18^ whereas cytidine 1 was inert under the same conditions.

As different products are afforded with two different neat solvents (H_2_O or formamide), different mixtures of these two solvents were then applied to the photoreaction of uridine to investigate the solvent effects. As can be seen from the results summarized in Table 1, formamide and water react competitively with uridine in the mixtures. In a 1 : 1 (v : v) mixture of the two solvents, 4 and 6 are formed in similar yields; however, we observed homogenous product compositions when the solvent purities reach above 75%. As formamide could be readily available by hydration of hydrogen cyanide in H_2_O,^17^ mixtures of formamide and H_2_O could have operated as a mixed solvent on early Earth. The composition (ratio) of the mixture would likely depend on the extent of heating on the primitive Earth. Due to formamide's lower volatility relative to water, it could have become concentrated.^19^

**Table 1:** Summary of ratios of products from photoreactions of uridine 2 (16 mM) in different compositions of solvent mixture

Entry	Ratio of solvents formamide : H_2_O	Ratio of products 6 : 4 : 2^a^
1	90 : 10	100 : 0 : 0
2	75 : 25	90 : 0 : 10
3	50 : 50	35 : 45 : 20
4	25 : 75	5 : 90 : 5
5	10 : 90	0 : 87 : 13

a The remaining uridine 2 resulted from dehydration of the photohydrate 4 during evaporation of formamide.

Photoreaction of uridine 2 in formamide was also investigated at varying concentrations, as shown in Table 2. At additionally higher starting concentrations, formamide adducts 7a/b were formed as a pair of diastereomers at C6 of the uracil moiety (entries 2–4). We propose that the adducts 7a/b are formed by radical recombination of hydrouridyl radicals and formamide radicals, which is favored by higher concentrations of uridine (*vide infra*).

**Table 2:** Summary of yields of products from photoreactions of uridine 2 at different concentrations in formamide. Yields are based on relative integration of the signals in ^1^H NMR spectra compared to an internal standard (sodium succinate)

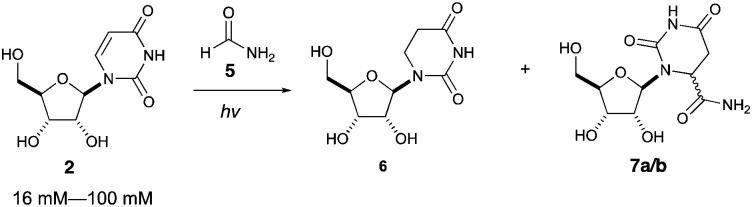			
Entry	[Uridine]/mM	Yields of products	
		6 (%)	7a/b (%)
1	16	94	—
2	32	80	18
3	60	53	37
4	100	35	35

We then investigated photoreduction of uridine nucleotides at lower concentration (16 mM) (Fig. 2). All uridine nucleotides 8–10 are efficiently converted to dihydrouridine nucleotides 11–13 (64–72% yield), providing substrates for efficient activation chemistry^20–22^ and non-enzymatic ligation chemistry^23,24^ to incorporate this modification into RNA molecules. Uridine in RNA short oligomers (trimers, such as UAA and UAC in Fig. S59 and S60, ESI†) could not be converted to dihydrouridine under similar reaction conditions. However, the monomer of dihydrouridine could still potentially be incorporated into short oligomers by self-polymerization,^21^ and then longer oligomers by template-free loop closing ligation.^23^

**Fig. 2:**
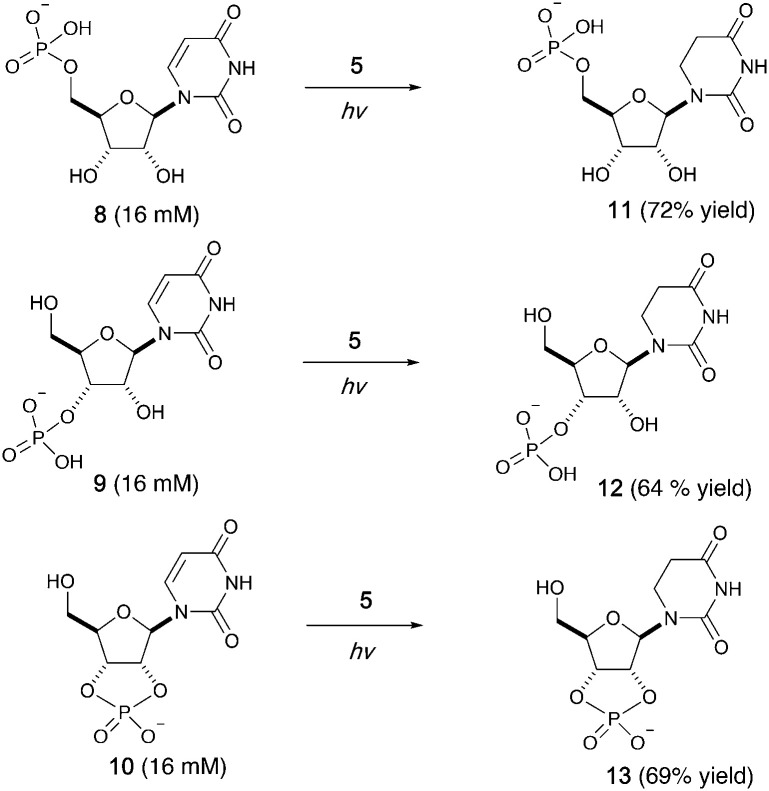
Photoreduction of uridine nucleotides to dihydrouridine nucleotides. Yields are based on relative integration of the signals in ^1^H NMR spectra compared to an internal standard (sodium succinate).

Adopting a systems chemistry approach, we then considered all four RNA nucleosides in the same geochemical scenario. Hence, mixtures of cytidine (C), uridine (U), adenosine (A, 14) and guanosine (G, 15) (all at 8 mM) were subjected to UV irradiation in formamide for 5 hours. 70% of the uridine 2 was converted to dihydrouridine 6 while the other three components of the canonical alphabet remained intact, resulting in a mixture containing C, U, A, G and DHU. This result suggests that the modified nucleoside, DHU, was potentially produced alongside the four canonical nucleosides (C, U, A, G) on early Earth. Whether it played a similar important role in the origin of life as it does in modern biology or was used as a building block in a later stage is unclear. UV irradiation is not only a driving force for the whole process and provides energy for photochemical synthesis, but also applies chemical selection pressure on the system to favor the synthesis of biomolecules that only function in extant biology (Fig. 3).

**Fig. 3:**
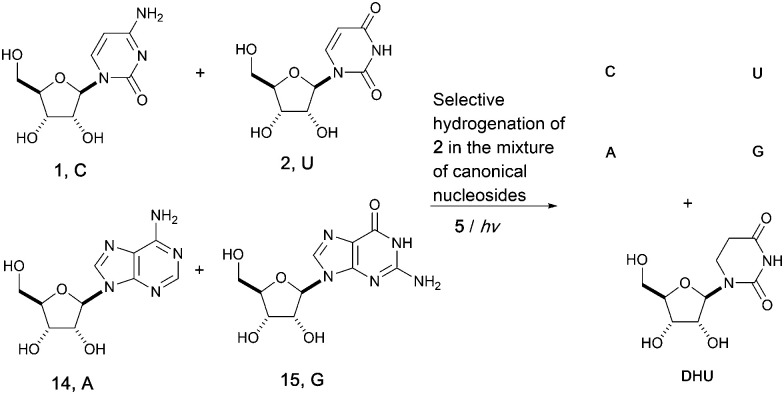
Limited UV irradiation of mixtures of canonical ribonucleosides^*b*^ (C, U, A, G) leads to a mixture of dihydrouridine (DHU, modified nucleoside in tRNA) with all four RNA canonical nucleosides (C, U, A, G). ^*b*^ Thymidine is not reactive under the same conditions.

In order to get clues as to the mechanism of the photoreduction, irradiation of uridine 2 was conducted in deuterated formamide (*N-d*_2_-formamide 16 or 1-*d*-formamide 17). The photoreaction of 2 (60 mM) in *N-d*_2_-formamide 16 afforded both 6 and 7a/b. Deuterium was partially incorporated at both the 5- and 6-positions of 6 while only the 5-position of 7a/b was deuterated, as confirmed by ^13^C-NMR spectroscopy (Fig. S24, ESI†) and integration of the corresponding signal in the ^1^H-NMR spectra (Fig. S23, S26 and S27 in ESI†). In comparison, the photoreaction with 2 again at 60 mM concentration in 1-*d*-formamide 17 only gave the reduction product 6a and deuterium was only partially incorporated at the 5-position of 6a (Fig. 4c and Fig. S21, S22, ESI†).

**Fig. 4:**
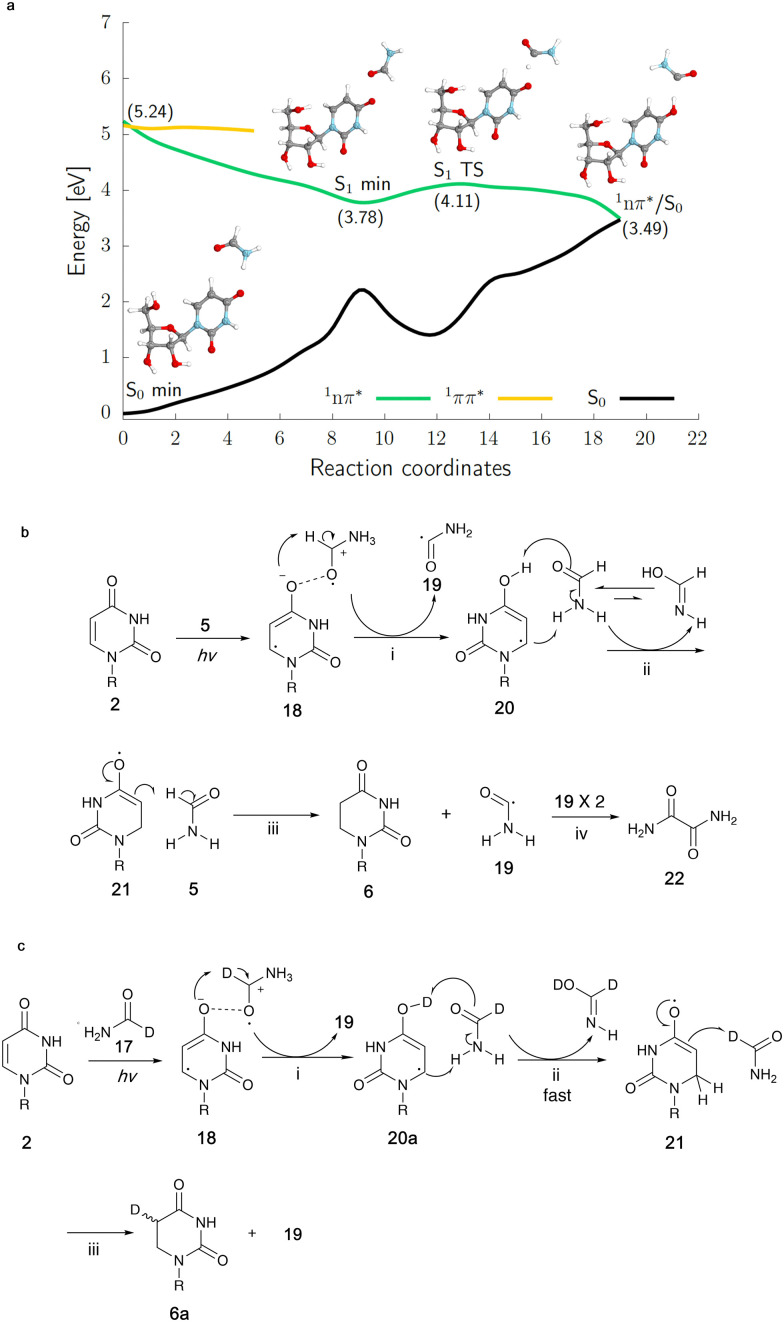
The ^1^nπ* excited-state potential energy surface (green curve) of the uridine-formamide complex presents an electron-driven proton transfer from formamide to uridine resulting in reactive hydrouridyl and formamide radicals. The results were obtained using the SCS-ADC(2) method and the aug-cc-pVDZ basis set. (a) Quantum-chemical calculations of uridine in formamide upon irradiation. (b) Mechanism of reduction of uridine in formamide inferred from calculations. (c) Reduction of uridine in 1-*d*-formamide. (i) Proton transfer following electron transfer; (ii) solvent-assisted tautomerization; (iii) hydrogen atom abstraction; (iv) radical recombination. R = β-d-ribofuranosyl.

To elucidate the origins of products 6 and 7a/b and the molecular mechanism of their formation during continuous UV irradiation, we performed quantum-chemical simulations exploring the photochemical reactivity of 2 in formamide 5 as well as its deuterated forms. This photoreactivity is reflected by our resulting excited-state potential energy surfaces for the uridine–formamide complex shown in Fig. 4a and b, computed with the highly correlated SCS-ADC(2) method and validated with the multiconfigurational XMS-CASPT2 approach (see the ESI†). The photoreduction of 2 to dihydrouridine (DHU) is initiated by the formation of an excited-state charge transfer (CT) complex 18, having an intermolecular interaction between the carbonyl oxygens (O⋯O distance of 2.22 Å) of uridine and formamide, in the lowest-lying ^1^nπ* singlet state (S_1_). Formation of this CT complex involves the transfer of 0.20 e^−^ from the neighboring solvent molecule to the pyrimidine ring, which initiates the photoreduction process. If the transferred negative charge is followed by a proton transfer from the formamide molecule to the C(4)=O oxygen of uridine, the system may undergo photorelaxation to the electronic ground state *via*^1^nπ*/S_0_ state crossing. We argue that the proton transfer occurs solely from the C–H position of formamide. This results in the formation of a carbon-centered formamide radical 19, which is more stable than the N-centered formamide radical by 17.1 kcal mol^−1^. The resultant hydrouridyl radical 20 may further tautomerize to its more stable form 21*via* solvent assisted hydrogen atom transfer. Subsequent abstraction of another hydrogen atom from another formamide molecule to the C5-position of the partially hydrogenated pyrimidine ring yields 6, whereas the two C-centered formamide radicals 19 may undergo radical coupling furnishing oxamide 22 (in 20% yield for the photoreaction of 60 mM of 2 in formamide), which was also identified among the photoproducts of our irradiation experiments (confirmed by a spiking experiment with authentic standard in ^13^C NMR, Fig. S28 in the ESI†). It is worth noting that we were not able to locate any analogous excited-state CT complexes in the case of cytidine complexed with explicit formamide molecules, which is also consistent with the lack of cytidine photoreduction in our experiments.

Higher yields of 7a/b from the irradiation in *N-d*_2_-formamide can be ascribed to substantially slower tautomerization of the initially formed enol form of the hydrouridyl radical 20. When the formamide molecules act as bridges for hydrogen atom relay, H to D exchange results in a six-fold decrease of the tautomerization rate (see our DFT-D calculations in the ESI†) and longer lifetime of the enol hydrouridyl radical 20, which may more easily undergo radical coupling with the formamide radical 19 yielding 7a/b. The deuteration of 6 in the C6-position is observed solely in the *N-d*_2_-formamide reaction and further supports our mechanism involving hydrouridine tautomerization. Selective deuteration of 6a in the C5-position observed in the 1-*d*-formamide reaction corroborates the computational suggestion that the final hydrogen atom abstraction (Fig. 4c(iii)) yields the carbon-centered radical of formamide 19 (detailed explanation for deuteration data in the ESI†). Overall, the proposed mechanism for the generation of 6 is very similar to the previously proposed mechanism for the formation of cytidine photohydrates^25^ or guanosine photolesions.^26^

In summary, dihydrouridine (DHU), a very common modification found in extant tRNA, can be highly efficiently synthesized from its parent canonical nucleoside, uridine (U), in formamide under UV irradiation. Moreover, preliminary data indicates that the hydrolysis product of formamide, formate, can also act as a reductant in water to reduce uridine to dihydrouridine, but in lower yield (50%, Fig. S57, ESI†). From theoretical calculations and deuterium exchange experiments, formamide is acting as a reducing reagent (electron donor or hydrogen atom donor) in the prebiotic conversion while UV irradiation serves as the key energy source to drive the reduction. Under the same prebiotic conditions, the other canonical nucleosides (cytidine, adenosine, guanosine) remain intact, suggesting that dihydrouridine might have coexisted with all four canonical RNA nucleosides (C, U, A, G) at the dawn of life. Whether dihydrouridine played an important role at the origin of life, as it does in modern biology, relies on the efficiency of its incorporation into primitive RNA oligomers. This remains to be investigated.

J. X and J. D. S acknowledge the Medical Research Council (grant no. MC_UP_A024_1009 to J. D. S.) and the Simons Foundation (grant no. 290362 to J. D. S.) for the support. M. J. J and R. S. acknowledge computational resources granted by the Wroclaw Centre for Networking and Supercomputing (WCSS). All authors thank Dr T. Rutherford for assistance with NMR spectroscopy.

## Conflicts of interest

The authors declare no conflicts of interest.

## Supplementary Material

CC-060-D4CC01823K-s001
