# Supplementary material for: Prebiotic synthesis of dihydrouridine by photoreduction of uridine in formamide
Source: Chem Commun (Camb). 2024 Jun 10;60(55):7081–4. doi: 10.1039/d4cc01823k (PMC11223185; doi:10.1039/d4cc01823k)
Supplement: CC-060-D4CC01823K-s001 [file CC-060-D4CC01823K-s001.pdf]

Electronic Supplementary Material (ESI) for ChemComm.
This journal is © The Royal Society of Chemistry 2024

Prebiotic synthesis of dihyrodrouridine by photoreduction of uridine in formamide

Jianfeng Xu, *[a] Mikołaj J. Janicki,*[b] Rafał Szabla[c] and John D. Sutherland[a]

a. MRC Laboratory of Molecular Biology, Francis Crick Avenue, Cambridge Biomedical Campus, Cambridge, CB2 0QH, UK.

b. Department of Physical and Quantum Chemistry, Faculty of Chemistry, Wrocław University of Science and Technology, Wybrzeże Wyspiańskiego 27, 50-370, Wrocław, Poland.

c. Institute of Advanced Materials, Faculty of Chemistry, Wrocław University of Science and Technology, Wybrzeże Wyspiańskiego 27, 50-370, Wrocław, Poland.

*Correspondence to: jxu@mrc-lmb.cam.ac.uk; mikolaj.janicki@pwr.edu.pl

This PDF file includes:

Materials and Methods
Theoretical section
Supplementary Figures S1 – S61
References

Materials and Methods

General Methods

Reagents and deuterated solvents used for reactions were purchased from Sigma-Aldrich or Acros Organics and were used without further purification. All photochemical reactions were carried out in Norell Suprasil quartz NMR tubes purchased from Sigma-Aldrich using Hg lamps with principal emission at 254 nm in a Rayonet photochemical chamber reactor RPR-200, acquired from The Southern New England Ultraviolet Company. A Mettler Toledo SevenEasy pH Meter S20 was used to monitor the pH, and deoxygenation of solution was achieved by sparging anhydrous argon through the solution for 15-20 min. A preparative Varian Prostar HPLC System was used for the reverse phase high-pressure liquid chromatography (RP-HPLC) linked with an Atlantis T3 C18 Prep Column OBD 10 µm (19 × 250 mm). An Analytical HPLC was used to monitor the reaction, using a Thermofisher Ultimate 3000 UPLC and Waters Atlantis T3 C18 column (5 µm, 4.6 mm × 150 mm). All unknown compounds in the reaction mixtures were confirmed by spiking experiments with authentic compounds either purchased from Sigma-Aldrich or synthesized in house using conventional synthetic chemistry. ^{1}H and ^{13}C NMR spectra were acquired using a Bruker Ultrashield 400 Plus operating at 400.1 MHz and 100.6 MHz respectively. Samples consisting of H_2O/D_2O mixtures were analysed using HOD suppression to collect ^{1}H NMR data. The quantitative ^{13}C NMR spectra were acquired with inverse-gated decoupling, with a 90˚ excitation pulse and an inter-pulse delay of 70 seconds. ^{13}C longitudinal relaxation time constants (T1) were measured to be no greater than 13 seconds for any of the ^{13}C resonances at 100.6 MHz. Chemical shifts (δ) are shown in ppm. The conversion yields were determined by relative integrations of the signals in the ^{1}H NMR spectrum. Coupling constants (J) are given in Hertz (Hz) and the notations s, d, m represent the multiplicities singlet, doublet, and multiplet signal. Mass spectra

were recorded with an Agilent Technologies 6130 Quadrupole LC-MS using positive and negative Electron Spray Ionisation.

Photoreaction procedures

Photoreaction of uridine in formamide in different concentrations.

Uridine (16 mM, 32 mM, 60 mM or 100 mM) in degassed formamide (1 mL) was transferred to a Suprasil quartz NMR tube and irradiated at 254 nm for 2-14 hours until the starting material was all consumed. The tube was periodically removed from the reactor to record ^{1}H NMR spectra. Yields were determined based on relative integrations of the signals referred to an internal standard (150 or 250 ul of 50 mM sodium succinate in H_2O) in ^{1}H NMR spectra. Yields for the various products are listed in Table 1.

Photoreaction of uridine in water.

Uridine (16 mM) in degassed water (0.5 mL) was transferred to a Suprasil quartz NMR tube and irradiated at 254 nm for 2 hours until the starting material was all consumed. The tube was periodically removed from the reactor to record ^{1}H NMR spectra. The water was removed by evaporation and the residue was taken up in formamide (0.5 mL) again. The solvent was removed by evaporation under high vacuum at 60 ºC before monitoring the reaction mixture with ^{1}H NMR. About 46% of uridine was reformed from uridine hydrate according to the observations on ^{1}H NMR spectrum (Figure S56).

Photoreaction of uridine in water in the presence of sodium formate.

Uridine (2 mg, 0.008 mmol) and sodium formate (7 mg, 0.10 mmol) were mixed in degassed water (0.5 mL), then transferred to a Suprasil quartz NMR tube and irradiated at 254 nm for 2 hours until the starting material was all consumed. The tube was periodically removed from the reactor to record ^{1}H NMR spectra (Figure S57).

Photoreaction of uridine in formamide/water mixtures.

Uridine (16 mM) in degassed formamide/water mixtures (0.5 mL, containing 10%, 25%, 50%, 75% or 90% of water, respectively) was transferred to a Suprasil quartz NMR tube and irradiated at 254 nm for 2 hours. At the end of reaction, the tube was removed from the reactor to record ^{1}H NMR spectra. The ratios of products were determined based on relative integrations of the signals in ^{1}H NMR spectra, which are listed in Table 2.

UV irradiation of thymidine at 254 nm.

Thymidine (16 mM, 32 mM, 60 mM or 100 mM) in degassed formamide (0.5 mL) was transferred to a Suprasil quartz NMR tube and irradiated at 254 nm for 2 hours. No reaction was observed from ^{1}H NMR spectra (Figure S58).

UV irradiation of trimer UAA at 254 nm.

UAA trimer (2 mg) in degassed formamide (0.5 mL) was transferred to a Suprasil quartz NMR tube and irradiated at 254 nm for 2 hours. No reaction was observed from ^{1}H NMR spectra (Figure S59).

UV irradiation of trimer UAC at 254 nm.

UAC trimer (2 mg) in degassed formamide (0.5 mL) was transferred to a Suprasil quartz NMR tube and irradiated at 254 nm for 2 hours. No reaction was observed from ^{1}H NMR spectra (Figure S60).

Preparation and characterization of 5, 6-dihydro-6(*S*, *R*)-carboxamido-uridine.

Uridine (61 mg, 0.25 mmol) in 5 ml of formamide was transferred to a Suprasil quartz tube (purchased from Cambridge Glassblowing Ltd), then irradiated at 254 nm for 9 hours. The solvent was removed by evaporation under high vacuum and the residue was dissolved in water, then subjected to preparative reverse phase HPLC, using acetonitrile in water as the eluent, to afford two pure diastereomers in 17 % (12 mg, 0.042 mmol) and 20% (14 mg, 0.050 mmol) yield respectively.

Isomer 1 : ^{1}H NMR (400 MHz, D_2O): δ 5.82 (1H, d, J = 6.2 Hz, H1'), 4.50 (1H, dd, J_1= 7.3 Hz, J_2 = 1.7 Hz, H6), 4.21 (1H, t, J = 5.9 Hz, H2'), 4.08 (1H, dd, J_1= 5.7 Hz, J_2 = 4.0 Hz, H3'), 3.96 (1H, m, H4'); 3.67 (2H, m, H5'); 3.15 (1H, dd, J_1= 17.4 Hz, J_2 = 7.3 Hz, H5); 2.82 (1H, dd, J_1= 17.4 Hz, J_2 = 1.6 Hz, H5). ^{13}C NMR (101 MHz, D_2O) δ 174.5 (carboxamido-C), 171.4 (C4), 154.2 (C2), 88.0 (C1'), 83.9 (C4'), 71.7 (C2'), 70.2 (C3'), 61.4 (C5'), 50.5 (C6), 33.7 (C5); ESI-LCMS (neg. m/z): 288.1 $[M-H]^-$.

Isomer 2: ^{1}H NMR (400 MHz, D_2O) δ 5.77 (1H, d, J = 6.1 Hz, H1'), 4.68 (1H, m, H6), 4.11 (1H, t, J = 5.8 Hz, H2'), 4.04 (1H, dd, J_1= 5.5 Hz, J_2 = 4.0 Hz, H3'); 3.96 (1H, m, H4'); 3.76 (1H, dd, J_1= 12.6 Hz, J_2 = 3.2 Hz, H5'); 3.67 (1H, dd, J_1= 12.6 Hz, J_2 = 4.5 Hz, H5'); 3.13 (1H, m, H5); 2.85 (1H, dd, J_1= 17.3 Hz, J_2 = 1.6 Hz, H5). ^{13}C NMR (101 MHz, D_2O) δ 174.0 (carboxamido-C), 172.0 (C4), 156.4 (C2), 88.5 (C1'), 82.2 (C4'), 72.5 (C2'), 70.1 (C3'), 61.4 (C5'), 49.7 (C6), 34.0 (C5); ESI-LCMS (neg. m/z): 288.1 $[M-H]^-$.

Preparation of 1-d_1-formamide.

6 ml (98.26 mmol) of d_1-methyl formate (99 atom % D, Sigma -Aldrich) was mixed with 100 ml of 7 M NH_3 in methanol in a sealed tube. The mixture was stirred at room temperature for 30 mins. Solvent was removed by evaporation under high vacuum to give 4.14 g (91.6% yield) of 1-d_1-formamide which was used without further purification.

Preparation of *N*-d_2-formamide.

5 ml (125.89 mmol) of formamide was mixed with 10 ml of D_2O for 30 mins before removing the solvents (H_2O, HDO, D_2O) at lower boiling point under high vacuum. The procedure was repeated for three times to obtain 5 g of *N*-d_2-formamide (90 atom% D, 85 % yield) which was used without further purification.

Photoreaction of uridine in 1-d_1-formamide or *N*-d_2-formamide.

15 mg (0.06 mmol) of uridine in 1ml of 1-d_1-formamide or *N*-d_2-formamide was irradiated at 254 nm until starting material was all consumed. The solvent was removed under high vacuum. The residue was dissolved in D_2O to record the crude ^{1}H NMR spectrum, then subjected to preparative reverse phase HPLC, using acetonitrile in water

as the eluent, to afford pure partially deuterated dihydrouridine and 5, 6-dihydro-6(*S*, *R*)-carboxamido-uridine. (Fig. S21-27)

Photoreaction of other ribonucleosides (C, A, G, I) in formamide.

Cytidine, adenosine, guanosine or inosine (16 mM) in degassed formamide (0.5 mL) was transferred to a Suprasil quartz NMR tube and irradiated at 254 nm for 2 hours, a time period during which uridine is all converted to dihydrouridine. The solvent was then removed under high vacuum. The residue was dissolved in D_2O to record the crude ^1H NMR spectra. No reaction from these ribonucleosides was observed in ^1H NMR spectra.

Photoreaction of mixtures of cytidine and uridine in formamide.

The mixture of cytidine and uridine (16 mM each) in degassed formamide (0.5 mL) was transferred to a Suprasil quartz NMR tube and irradiated at 254 nm for 6 hours, during which uridine is all converted to dihydrouridine. The solvent was then removed under high vacuum, and the residue was dissolved in D_2O to record the crude ^1H NMR spectra. No reaction from cytidine was observed in ^1H NMR spectrum.

Photoreaction of mixtures of all four canonical ribonucleosides (U, C, A, G) in formamide.

A mixture comprising 1 mg each of uridine, cytidine, adenosine and guanosine in formamide (0.5 mL) was transferred to a Suprasil quartz NMR tube and irradiated at 254 nm for 5 hours in total. The solvent was then removed under high vacuum. The residue was dissolved in D_2O to record the crude ^1H NMR spectrum. 78% of uridine was converted to dihydrouridine while all other three ribonucleosides remained intact.

Photoreaction of uridine-5'-phosphate in formamide.

6 mg (0.016 mmol) of uridine-5'-phosphate in 1ml of formamide was transferred to a Suprasil quartz NMR tube and irradiated at 254 nm for 2 hours. The solvent was then removed under high vacuum. The residue was dissolved in D_2O to record the crude ^1H NMR and ^{31}P NMR spectra. 60 uL of 50 mM sodium succinate was added to the mixture to determine the yield of dihydrouridine-5'-phosphate by relative integration (72% yield).

Photoreaction of uridine-3'-phosphate in formamide.

6 mg (0.016 mmol) of uridine-3'-phosphate in 1ml of formamide was transferred to a Suprasil quartz NMR tube and irradiated at 254 nm for 2 hours. The solvent was then removed under high vacuum. The residue was dissolved in D_2O to record the crude ^1H NMR and ^{31}P NMR spectra. 60 uL of 50 mM sodium succinate was added to the mixture to determine the yield of dihydrouridine-3'-phosphate (64% yield).

Photoreaction of uridine-2',3'-phosphate in formamide.

2 mg (0.006 mmol) of uridine-2',3'-phosphate in 0.5ml of formamide was transferred to a Suprasil quartz NMR tube and irradiated at 254 nm for 2 hours. The solvent was then removed under high vacuum. The residue was dissolved in D_2O to record the crude ^1H NMR and ^{31}P NMR spectra. 60 uL of 50 mM sodium succinate was added to the mixture to determine the yield of dihydrouridine-2',3'-phosphate (69% yield).

Theoretical section

Computational methods

To locate the most stable geometries of uridine in a polar solvent, the conformational space was studied using the Conformer-Rotamer Ensemble Sampling Tool[1] (CREST) at the semi-empirical GFN2-xTB[2] level of theory. The conformational space was explored using the iMTD-GC scheme together with the analytical linearized Poisson–Boltzmann (ALPB) implicit solvent model, assuming a water solvent. The optimized ground-state structures and the corresponding harmonic vibrational frequencies for isolated uridine and the uridine and formamide complex were located using the Kohn–Sham density functional theory (KS-DFT) and the range-separated hybrid ωB97X-D functional including dispersion correction.[3] These calculations were performed using the def2-TZVP[4] basis set. To estimate solvent effects exerted by formamide on our model systems, the polarizable continuum model[5,6] (PCM) with the integral equation formalism variant (IEFPCM) was applied. The level of theory is further described as the PCM/ωB97X-D/def2-TZVP abbreviation. The KS-DFT and PCM computations were performed by means of the Gaussian[7] 16 package.

Vertical excitation energies of the uridine and formamide complex were obtained, using the spin-component scaled variant[8] of the algebraic diagrammatic construction to the second order method[9,10] [SCS-ADC(2)] with the aug-cc-pVDZ[11] basis set (SCS-ADC(2)/aug-cc-pVDZ). For these calculations we used the S_0 geometries optimized at the PCM/ωB97X-D/def2-TZVP level of theory. The molecular orbital character of lowest-lying excited states was characterized through the analysis of natural transition orbitals (NTOs). The charge transferred between the explicit formamide molecule and the nucleoside was evaluated using the one-electron transition density matrix (1TDM) and the Löwdin style analysis. The 1TDM and NTOs analyses were performed employing the TheoDore[12] 3 package. The crucial points on excited-state potential energy surfaces of the studied system were found at the SCS-ADC(2)/aug-cc-pVDZ level of theory. The minimum-energy crossing point (MECP) between the first excited and ground state was optimized using the sequential penalty constrained optimization method implemented in the CIOpt[13] package. The MECP was optimized utilizing the energies and analytical gradients obtained using the MP2/ADC(2)/aug-cc-pVDZ. The CIOpt and Turbomole[14] 7.3 programs were interfaced to perform the MECP optimization. The potential energy profiles (PEPs) of the photochemical reaction were constructed using the image dependent pair potential (IDPP) interpolation[15] between the optimized S_0 structure and the S_1 minimum-energy geometry. The PEPs, between the S_1 minimum and the S_1 transition-state structure, were built with geometries generated utilizing the reaction path (RP) optimization method[16] implemented in the Turbomole[14] 7.3 program. To obtain the PEPs from the S_1 transition-state structure towards the S_1/S_0 minimum-energy, the intrinsic reaction coordinate method[17] (IRC) was employed. The RP and IRC methods allowed us to obtain smooth potential energy profiles between the crucial excited-state points. All optimized structures and the energies of ground and excited states were located using the SCS-MP2/aug-cc-pVDZ and SCS-ADC(2)/aug-cc-pVDZ methods, respectively. All excited-state calculations were performed using the Turbomole[14] 7.3 package. The IDPP method was used in the ORCA[18] 5.0.3 program.

To benchmark the single-reference SCS-ADC(2) method which was used for the optimization of excited-state minimum-energy structures and the S_1/S_0 minimum-energy crossing point, the critical excited-state geometries were reoptimized with the extended multi-state complete active space second-order perturbation theory[19,20]

(XMS-CASPT2), based on the state-averaged complete active space self-consistent field (SA-CASSCF) method with the cc-pVDZ basis set. To find the S_1 minimum-energy structure at the XMS-CASPT2 level, the complete active space (CAS) was built of molecular orbitals (MOs) having natural orbital occupations in the range of 0.02–1.98,[21] and contained one σ, three π, and one n_O occupied MOs alongside with three π^* and one σ^* virtual MOs (overall 10 electrons were correlated in 9 orbitals, as shown in Fig. S48, left panel). The SA-CASSCF wave function was averaged over three lowest-lying electronic states. The S_1/S_0 minimum-energy conical intersection was optimized assuming the CAS constructed of the three π, n_O, and four π^* MOs (10 electrons in 9 orbitals, shown in Fig. S48, right panel). In both cases, the SA-CASSCF wave function was averaged over three lowest-lying electronic states. The vertical shift, i.e., the empirical correction applied to the zeroth-order Hamiltonian, was equal to 0.4 Hartree, and the XMS-CASPT2 calculations were conducted using the BAGEL[22] 1.2.0 package.

The ground-state structures of substrates, transition states and products of studied chemical reactions were obtained at the PCM/ωB97X-D/def2-TZVP level of theory, assuming the IEFPCM model of bulk formamide. To evaluate the kinetic isotope effect for a given chemical process, we recalculated the thermal corrections to Gibbs free energy for the corresponding substrate and transition-state structures, replacing a hydrogen atom with deuterium at a given site, to estimate a new energy barrier. Then, the following formula $k_H/k_D = e^{-(\Delta G_H^{\ddagger}-\Delta G_D^{\ddagger})/RT}$ was used, in which the $\Delta G_H^{\ddagger}$ *and* $\Delta G_D^{\ddagger}$ corresponding to the relative difference Gibbs free energies between the substrate and transition state structures for formamide and deuterated formamide molecules, respectively. In addition, the R and T variables are the gas constant and temperature (298 K), respectively. The ratio k_H/k_D expresses the change of the reaction rate when the studied system is isotopically substituted with deuterium.

Equilibrium ground-state structure of uridine and its complex with formamide

To find the equilibrium ground-state geometry of β-uridine in formamide solution, we conducted the semi-empirical GFN2-xTB[2] calculations to explore the conformational space of the canonical pyrimidine nucleoside. Subsequently, we selected the most stable C2'-endo and C3'-endo structures of β-uridine, and then we reoptimized these structures (Fig. S41) at the PCM/ωB97X-D/def2-TZVP level of theory, assuming the continuum model of bulk formamide, and the corresponding harmonic vibrational frequencies were also computed. Our Gibbs free energy computations show that the C2'-endo and C3'-endo conformers of uridine are almost isoenergetic, and the corresponding energy difference amounts to -0.03 kcal/mol. This result indicates that both conformers of β-uridine can coexist in an equilibrium in formamide solution. The key structural difference between the considered conformers, apart from the conformational arrangement of the sugar part, is an intramolecular hydrogen bond (1.93 Å) between the hydroxyl group at the C2' atom (sugar fragment) and the carboxyl oxygen atom at the C2 atom (nucleobase part) that occurs only in the C2'-endo structure (see Fig. S41). Since the C2=O moiety of the nucleobase can create the intramolecular hydrogen bond with the C2'-endo sugar, we anticipate that this carbonyl group might only periodically interact with neighbouring formamide molecules, when it is not involved in the intramolecular hydrogen bond. Thus, we added an explicit quantum-mechanical formamide molecule at the C4=O carbonyl group of the chromophore and optimized the geometry of this complex (Fig. S42) using the PCM/ωB97X-D/def2-TZVP method. In the complex (Fig. S42), there is a hydrogen bond of 1.93 Å between the uridine C4=O carbonyl oxygen and the amino group of formamide. Importantly, an explicit formamide solvent molecule can also interact in an

unconstrained manner with the C3'-endo conformer at the C4=O site, thus we assume that both conformers should exhibit similar photochemical reactivity in formamide solution. In this work, our aim was to investigate the active participation of a single neighbouring formamide molecule in the photochemistry of β-uridine in order to elucidate the photoreduction mechanism of uridine to dihydrouridine.

Photophysical properties of uridine and formamide complex

For the optimized S_0 structure of the uridine and formamide complex, we calculated vertical excitation energies and corresponding oscillator strengths for the three lowest-energy excited states at the SCS-ADC(2)/aug-cc-pVDZ level of theory, and the results are presented in Tab. S1.

According to these calculations, the first excited state at 5.16 eV (240.3 nm) is an optically bright state with a substantial oscillator strength of 0.299 and can be assigned as a $\pi\pi^*$ transition (see Fig. S43). The second excited state at 5.236 eV (236.8 nm) can be considered as a mostly dark state with insignificant contribution to the overall absorption given the oscillator strength of 0.015. In the case of the S_2 state, the electron is mainly promoted (see Fig. S43) from the lone electron pair orbital (n_O), localized at the C4=O site, to the π^* molecular orbital located on the aromatic ring of uridine. Both of these excited states lie energetically close to each other and can be easily populated in the Franck-Condon region of the investigated system. The third excited state is located at higher energy (5.656 eV and 219.2 nm) and is characterized by the $n_O\pi^*$ excitation (Fig. S43) occurring within the formamide molecule.

In our work, we irradiated samples using 254 nm wavelength (4.88 eV) to perform the photochemical reduction of uridine to dihydrouridine. In the previous paragraph, we pointed out that the photoexcitation of formamide, assuming 254 nm light, is very unlikely owing to significant energy separation of the S_3 state from the S_1 and S_2 states. Thus, the presented photophysical properties of the uridine and formamide complex indicate that irradiation at 254 nm populates excited states associated with the pyrimidine ring. Our next step was to investigate the photochemistry of the uridine-formamide complex based on explorations of excited-state potential energy surfaces beyond the Frank-Condon region.

Photochemistry of uridine and formamide complex

Exploring the S_1 excited-state potential energy surface of the uridine and formamide complex (Fig. S44) at the SCS-ADC(2)/aug-cc-pVDZ level of theory, we found that our system, after photoexcitation, can easily reach the S_1 excited state having the $n_O\pi^*$ molecular orbital character. As demonstrated by previous experimental and theoretical studies, high-energy vibrational motions of the UV-excited molecule can force the elongation of the C4=O carbonyl bond,[23–26] which lowers the energy of the $n_O\pi^*$ excited state. Since the $S_1(n_O\pi^*)$ excited-state region could be readily populated, we optimized a local S_1 minimum that is shown in Fig. S44. This $S_1(n_O\pi^*)$ minimum-energy structure is characterized by an excited-state intermolecular O···O interaction involving lone electron pair orbitals (Fig. S44) originating from the uridine and the formamide carbonyl oxygen atom, and the corresponding C4-O···O=C distance amounts to 2.22 Å. This intriguing excited-state oxygen-oxygen interaction replaced the ground-state hydrogen bond between uridine and formamide, and can significantly stabilize the UV-excited complex by lowering the energy of the system by 1.38 eV when compared to the vertical excitation energy of the optically bright state.

Furthermore, the wavefunction analysis of the S_1 minimum having the $n_O\pi^*$ transition (Fig. S44) indicated a charge-transfer (CT) process of 0.20 e^- from the n_O orbital of formamide to the π^* molecular orbital of uridine. Thus, the S_1 minimum-energy structure is an excited-state charge-transfer complex, in which the formamide molecule acts as an electron donor when both molecules are in an appropriate arrangement. It is worth adding that this kind of excited-state intermolecular interaction was also found and described for other chromophores in the presence of water molecules, including 7H-adenine,[27] methylated cytosine[23] and guanosine[24].

We next performed SCS-ADC(2) calculations of excited-state potential energy surfaces which show the plausible photochemical mechanism involving the CT complex as the key reactive species (see Fig. S45). After photoexcitation of the uridine and formamide complex to the optically bright excited state (orange line, at 5.16 eV), the system can easily populate the $^1n_O\pi^*$ excited state (green line) and then reach the charge-transfer S_1 minimum-energy structure at 3.78 eV. Having the excess energy originating from the electronic excitation, our model system can subsequently pass through the S_1 transition-state structure (at 4.11 eV), which involves an excited-state hydrogen atom transfer from the formamide carbon atom to the uridine carbonyl oxygen atom resulting in the formation of the biradical system. This photochemical reaction leading to the production of hydrouridyl and formamide radicals also leads to the S_1/S_0 state crossing (at 3.49 eV) that can serve as a radiationless deactivation channel to the electronic ground state. We have demonstrated that the formation of an excited-state intermolecular CT complex in the S_1 minimum can entail the hydrogen atom transfer process leading to the photoreduction of uridine by solvent formamide molecule. It is worth adding that the critical point for the whole photoreduction process is the S_1 transition-state geometry (at 4.11 eV) which lies about 1 eV below the vertical excitation energy of the $^1\pi\pi^*$ excited state (in the Franck-Condon region).

In addition to the easily accessible photoreduction process described in the previous paragraph, we found another plausible photochemical pathway in a different region of the $S_1(n_O\pi^*)$ hypersurface (see Fig. S46). After photoexcitation of the uridine and formamide complex, the system can readily reach the $^1n_O\pi^*$ excited-state potential energy surface as shown in Fig. S45. Subsequently, the model system can follow a distinct virtually barrierless photochemical pathway (see Fig. S46, green line) to the $S_1(n_O\pi^*)/S_0$ intersection seam at 4.55 eV, corresponding to the $S_1(n_O\pi^*)$ transition-state excited-state geometry on the S_1/S_0 crossing seam. In the optimized $S_1(n_O\pi^*)$ structure (see inset in Fig. S46) occurs an excited-state intermolecular O···O interaction engaging lone electron pair orbitals from the uridine and the formamide carbonyl oxygen atom, and the corresponding C4-O···O=C distance is of 2.02 Å. The excited-state intermolecular interaction facilitates a charge-transfer process of 0.41 e^- from the n_O orbital of formamide to the π^* molecular orbital of uridine in the S_1 state. Together with the excited-state charge transfer process, there is also a significant elongation of the N-H bond (by 0.23 Å) in the amino group towards the C5 atom of uridine. Since an electron and proton are being transferred from formamide to uridine during the single step of the photochemical mechanism, we classify the process as a concerted proton-coupled electron transfer mechanism (PCET). Consequently, in the vicinity of the S_1/S_0 crossing seam, the concerted PCET enables the hydrogen atom transfer from the amino group of formamide to the C5 atom of UV-excited uridine, yielding the nitrogen-centered formamide and uridine radicals. The former radical is energetically unstable (see Fig. S54) and should tautomerize to the carbon-centered formamide radical. It is worth emphasizing that the S_1 transition-state structure can serve as a pathway for

radiationless deactivation to the electronic ground state, as it is located at the S_1/S_0 intersection seam.

Both discussed photochemical processes demonstrate that this first crucial step of the photoreduction mechanism might be easily initiated photochemically and can occur via a stepwise or concerted mechanism. The latter involves the hydrogen atom transfer from the amino group to the C5 atom and is marked by a higher energetically photochemical pathway compared to the two-stepwise hydrogen atom transfer from the C-H site to the carbonyl oxygen atom of uridine (Fig. S45 and S46). Nevertheless, both excited-state processes are expected to play an important role in the photochemistry of uridine dissolved in a formamide solution.

Furthermore, our quantum-chemical calculations show that photoinduced reduction chemistry can be exclusively initiated by the electronic excitation of uridine. There is no need to excite solvent formamide molecules, for releasing solvated electrons, to conduct the photoreduction process.

Based on these static quantum-chemical calculations, we have found another example, in which ground-state solvent molecules can actively participate in the photochemistry of the chromophore.

Excited-state key structures at XMS-CASPT2 and SCS-ADC(2) methods

In the previous section, we discussed the plausible radiationless deactivation pathway as a photoreduction mechanism of UV-excited uridine utilizing formamide at the SCS-ADC(2) level. Recently, it was shown that the SCS-ADC(2) method[8] can offer accurate results for the description of the photochemistry of carbonyl-containing chromophores.[25] In particular, the method can physically represent the elongation of the carbonyl bond in heteroaromatic chromophores, while the original ADC(2) method[9,10] offers much less accurate excited-state potential energy surfaces and energies for this group of chromophores.[25] However, the single-reference SCS-ADC(2) method[8] still has some limitations, which should always be considered when a new photochemical mechanism is postulated at this level of theory. The reference electronic ground state is described by the single-reference SCS-MP2 method,[28] which cannot correctly describe multireference systems, and consequently, the S_1/S_0 surface crossing might be nonphysical in some cases. Therefore, we reoptimized the S_1 and S_1/S_0 minimum-energy structures (shown in Fig. S45), obtained using the SCS-ADC(2)/aug-cc-pVDZ method, employing the multireference XMS-CASPT2/SA-3-CASSCF(10,9)/cc-pVDZ level of theory. The active spaces used in the XMS-CASPT2 calculations are presented in Fig. S48. The superimposed XMS-CASPT2 and SCS-ADC(2) minimum-energy geometries are shown in Fig. S47 as silver and orange structures, respectively. To evaluate the similarity of the corresponding structures, we computed root-mean-square deviations (RMSDs), which amount to 0.113 and 0.223 Å for the S_1 and S_1/S_0 minimum-energy structures, respectively. It demonstrates that the SCS-ADC(2) and XMS-CASPT2 geometries are virtually identical. Thus, the SCS-ADC(2) method can be used for the studied system.

The key structural difference between the SCS-ADC(2) and XMS-CASPT2 $S_1(n_O\pi^*)$ minimum-energy geometries is the intermolecular C4-O···O=C distance equal to 2.22 and 2.50 Å, respectively. It is worth noting though that the excited-state $^1n_O\pi^*$ potential energy surface for increasing the O···O distance is very flat at the multireference XMS-CASPT2 level. More specifically, comparing the $S_1(n_O\pi^*)$ minimum (O···O distance is 2.50 Å) with the $S_1(n_O\pi^*)$ structure having the O···O distance equal to 2.28 Å, the corresponding energy difference only amounts to -0.74

kcal/mol at the XMS-CASPT2 level. Furthermore, our excited-state geometry optimizations of the model system were conducted in the gas phase without any solvent models. Thus, we cannot unequivocally assess the excited-state equilibrium intermolecular O⋯O distance that would be relevant in the presence of bulk formamide solvent. To estimate the charge-transfer character of the XMS-CASPT2 S_1 minimum, having the O⋯O distance equal to 2.50 Å, we performed the wavefunction analysis at the SCS-ADC(2) level of theory, indicating the intermolecular electron transfer is 0.10 e^-. It is worth adding that the excited-state CT complex should be energetically stabilized in a polar formamide solution[29] because the dipole moment of the S_1 CT state in implicit COSMO solvent model is larger by approximately 3 D compared to the gas-phase structure. Therefore, we conclude that since the XMS-CASPT2 approach has predicted the existing charge-transfer intermolecular O⋯O interaction, the SCS-ADC(2) method offers a reliable description of this S_1 minimum.

The SCS-ADC(2) S_1/S_0 minimum-energy crossing point (MECP) was predicted and confirmed by the XMS-CASPT2 calculations. The main structural difference is only associated with the position of the formamide radical with regard to the hydrouridine radical. The formamide radical is noticeably shifted towards the hydrouridine radical at the XMS-CASPT2 level compared to the corresponding SCS-ADC(2) structure. Considering the energetic availability of the S_1/S_0 MECPs, we found that the ⊗E between S_1 minimum and S_1/S_0 MECP at the XMS-CASPT2 and SCS-ADC(2) levels amounts to 0.84 and 0.29 eV, respectively. It indicates that both methods predicted a peaked S_1/S_0 conical intersection that might be readily energetically accessible.

In order to assess the optimized $S_1(n_O\pi^*)$ transition-state excited-state geometry on the S_1/S_0 crossing seam, at the SCS-ADC(2)/aug-cc-pVDZ level of theory (presented in Fig. S46), we performed a single-point calculation using the XMS-CASPT2/SA-2-CASSCF(10,9)/cc-pVDZ method. Our analysis revealed that the energy difference between the S_1 and S_0 states amounts to 0.57 eV at the XMS-CASTP2 level. Thus, the multireference calculations confirmed that the S_1 structure is situated close to the S_1/S_0 crossing seam and might be responsible for the radiationless deactivation mechanism.

In conclusion, the performed benchmark of the SCS-ADC(2) method with the multireference XMS-CASPT2 approach has shown that the SCS-ADC(2) results are valid and the single-reference method can be applied for the investigated system in this paper.

Excited-state complex of cytidine and formamide

Uridine and cytidine exhibit similar structural motifs in the chromophore part of their nucleosides. In particular, both contain the carbonyl oxygen atom attached to the aromatic structure, which possesses a photochemically reactive lone electron pair that could be involved in excited-state CT complexes (see above). Furthermore, the cytidine molecule has another lone electron pair associated with the N3 nitrogen atom that is a part of the pyrimidine ring. Since both molecules are characterized by similar structural features, we decided to also perform UV irradiation experiments with cytidine in a formamide solution. Under such conditions, we have not observed any photoreduction of cytidine to dihydrocytidine experimentally, as demonstrated for uridine.

To explain the striking difference in photochemical reactivity of both nucleosides, we performed quantum-chemical calculations for a complex of cytidine

with a single formamide molecule at the SCS-ADC(2)/aug-cc-pVDZ level of theory, and the results are shown in Fig. S49. Exploring the S_1 excited-state potential energy surface of the complex, we couldn't find a similar S_1 minimum that was found in the uridine and formamide complex presented in Fig. S44. Instead, we optimized another the $S_1(n\pi^*)$ minimum-energy structure (Fig. S49) in which there is an excited-state intermolecular interaction between lone electron pair orbitals of the cytidine nitrogen atom and the formamide carbonyl oxygen atom. Although a similar excited-state complex between these molecules is formed in the $S_1(n\pi^*)$ state, the corresponding molecular n and π^* orbitals are mainly localized on the formamide molecule. Consequently, our wavefunction analysis of the optimized S_1 structure revealed no intermolecular electron transfer between the chromophore and formamide. The lack of intermolecular charge-transfer mechanism necessary for the photoreduction process explains why cytidine cannot undergo a photochemical reduction in formamide solution.

Generation of dihydrouridine in the electronic ground state

Our photochemical computational studies concerning the uridine and formamide complex have shown that the system can undergo an excited-state intermolecular hydrogen atom transfer leading to the formation of the hydrouridyl and formamide radicals in the vicinity of the S_1/S_0 surface crossing (Fig. S45). UV-irradiation experiments of uridine in a formamide solution resulted in very efficient generation of dihydrouridine. Therefore, our next step was to elucidate the molecular mechanism of subsequent photoreduction of the hydrouridyl radical.

The hydrouridyl radical can undergo a tautomerization process in which a proton can be transferred through solvent formamide molecules from the C4-OH site to the C6 atom of the nucleoside (Fig. S50) yielding a tautomer more stable by 9.3 kcal/mol when compared to the enol form resulting from the S_1/S_0 state crossing. According to our KS-DFT ground-state calculations for this proton transfer process, the corresponding transition state is characterized by the energy barrier of 9.9 kcal/mol. In turn, the protonation at the C6 atom (Fig. S51, at -2.5 kcal/mol) leads to the more stable hydrouridyl radical tautomer and the enol tautomer of formamide is formed. It is worth adding that solvent molecules act as a proton relay during the tautomerization through a formamide molecule. Consequently, the solvent changes its tautomeric form from keto to enol or vice versa in the case of formamide. Therefore, to characterize the proton transfer process, we added two explicit formamide molecules to the hydrouridyl radical (Fig. S51). To avoid the unneeded generation of the enol form of formamide during the tautomerization reaction, we included one formamide molecule in the enol form and the other in the keto form. The formamide-assisted proton transfer entails two consecutive tautomerizations of the involved formamide molecules. During this process, one formamide molecule changes its form from enol to keto and the other from keto to enol. This approach allowed for maintaining the tautomeric balance of the solvent molecules that is unchanged during the hydrouridine radical tautomerization process. It is worth adding that single tautomerization of formamide from its keto to enol form is associated with ΔE of 16.39 kcal/mol.

Our theoretical explorations showed that the proton transfer process, allowing for tautomerization of the hydrouridine radical, is the lowest-energy mechanism that could occur shortly after the deactivation of the model system to the ground state. Subsequently, the most stable hydrouridyl radical can abstract a hydrogen atom from a nearby neutral formamide molecule, and the corresponding hydrogen atom abstraction

transition state was found at 13.0 kcal/mol (Fig. S52). Passing through this modest barrier results in the formation of neutral dihydrouridine and formamide radical (Fig. S52, 1.4 kcal/mol). It is worth noting here, that this pathway leading to the formation of a single dihydrouridine molecule involves the generation of two formamide radicals, one generated photochemically and one in the subsequent dark photoreaction. These formamide radicals may next undergo radical recombination to yield a single oxamide molecule which is also observed among the products of our irradiation UV experiments.

To further elaborate on the uridine photoreduction process and analyze the mechanism, we conducted additional UV-irradiations experiments in deuterated formamide, such as *N*-d_2-formamide or 1-*d*-formamide. The photochemical reaction of uridine in *N*-d_2-formamide yielded dihydrouridine and the formamide C6-adduct of uridine. Deuterium was found in the C5 and C6 positions of dihydrouridine, whereas only the C5 atom was deuterated in the formamide C6-adduct of uridine. In the *N*-d_2-formamide solution, a proton transfer mechanism, resulting in the formation of the most stable tautomer of the hydrouridine radical (Fig. S51) should be noticeably slower since the amino group of formamide which is involved in the proton relay process leading to the tautomerization possesses deuterium atoms. Based on our calculations, hydrouridine tautomerization is not the rate-limiting step, albeit we expected this H to D substitution on the amino group to result in a noticeable primary kinetic isotope effect on this particular step of the reaction. Slower tautomerization of the hydrouridine radical, could enable more efficient radical recombination process between the formamide radical and the C6 position of hydrouridyl radical, leading to the formation of formamide-uridine adduct. To estimate the extent of this effect in the case of *N*-d_2-formamide, we recomputed the Gibbs free energy values for the hydrouridine radical, and the corresponding transition state associated with the enol to C6 tautomerization (Fig. S51). The corresponding transition state with the *N*-d_2-formamide molecule has higher energy by approximately 1 kcal/mol compared to the same transition state structure involving non-deuterated formamide molecules. Consequently, our estimated kinetic isotope effect, that is the ratio of k_H/k_D is equal to 5.9. It indicates that the proton transfer process in an *N*-d_2-formamide solution should be 5.9 times slower versus a formamide solution. It is worth adding that in the entire tautomerization process of hydrouridine radical, occurring from the C4-OH to C6 site, requires a few proton transfers through N-d_2-formamide molecules. Consequently, since the proton transfer from the C4-OH to C6 site is significantly slower in an N-d_2-formamide solution, the photochemically generated formamide radical could have enough time to be attached to the C6 atom of hydrouridyl radical and form the formamide C6-adduct (Fig. S53). This explains why the C6-adduct is observed when the formamide solution is replaced by *N*-d_2-formamide. Furthermore, the proposed proton transfer mechanism also elucidates the incorporation of deuterium in the C6 atom of uridine. According to Fig. S52, the final step of dihydrouridine formation occurs through hydrogen atom transfer from the formamide molecule to the C5 atom of uridine. However, this process would not lead to the deuteration in the C5 position. The deuterated C5 site can be explained through a direct photochemical hydrogen atom transfer from the Nd_2 site of *N*-d_2-formamide to the C5 atom of uridine (Fig. S46). In other words, upon UV excitation of uridine, the Nd_2 site can act as an active donating group, allowing for deuteration at the C5 position, which is in line with our experimental results.

The photochemical reduction of uridine was also performed in 1-*d*-formamide solution, which led to the formation of dihydrouridine having only a partially deuterated C5 position. In the proposed mechanism of dihydrouridine generation, only the final step occurring in the dark (Fig. S52) might be altered by 1-*d*-formamide since the final

intermolecular hydrogen atom transfer takes place preferentially from the formamide C1 site to the uridine C5 atom, yielding the energetically most stable formamide radical at the carbon atom (see Fig. S54). Therefore, assuming the 1-*d*-formamide molecule, we recomputed the ground and transition states to evaluate the kinetic isotope effect (Fig. S52), and the ratio of k_H/k_D equal to 4.97 for this particular step. However, it is worth noting that this final intermolecular hydrogen atom transfer is not the rate determining step and the effect of deuteration on the overall reaction should be negligible. Consequently, the formation of the uridine C5 formamide adduct is highly unlikely because any nearby 1-*d*-formamide molecule can still efficiently donate its hydrogen atom with reaction rate that should exceed the diffusion rate of the formamide radical formed in previous steps. According to this mechanism, generated dihydrouridine should be selectively deuterated in the C5 position which very well matches the experimental results.

In summary, our mechanistic rationale proposed based on quantum chemical calculations is entirely consistent with the additional irradiation experiments performed in *N*-d_2-formamide and 1-*d*-formamide solvents since and it explains the selective deuteration of the photoproduct as well as formation of the formamide adduct and oxamide.

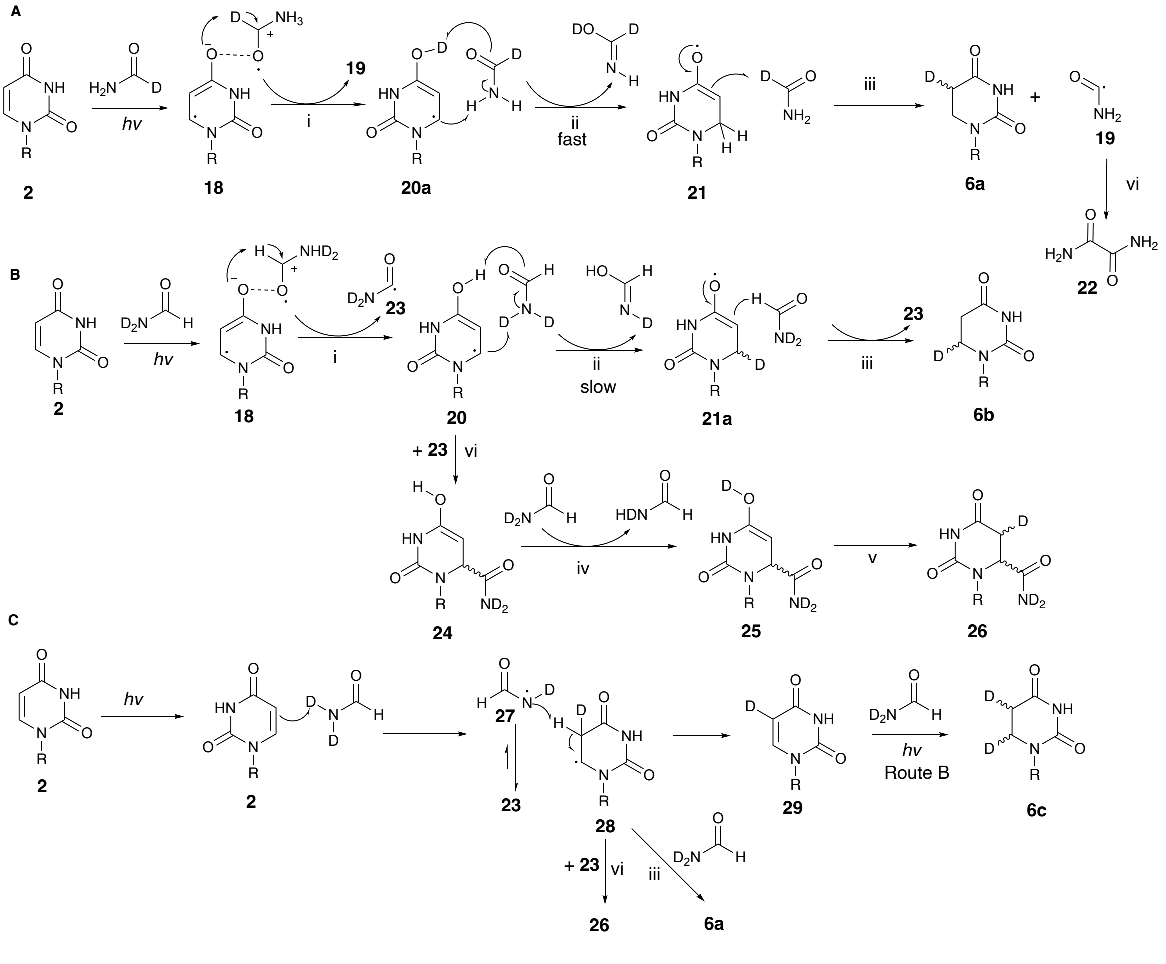

Scheme S1 Photochemical mechanism of uridine reduction in 1-*d*-formamide (Panel A) or *N*-d_2-formamide (Panel B and C). i) Proton transfer following electron transfer; ii) solvent-assisted tautomerization; iii) hydrogen atom abstraction from formamide;

iv) deuterium exchange; v) keto-enol tautomerization; vi) radical recombination. R= β-D-ribofuranosyl.

Discussion on deuteration data inferred from theoretical calculation.
The photoreductions in deuterated formamide are initiated by the formation of an excited-state charge transfer (CT) complex **18**. Electron transfer allows immediate proton transfer from C-D (panel A) or C-H (panel B) of deuterated formamide. The hydrouridyl radical **20a** or **20** subsequently tautomerizes to its more stable form **21** or **21a** via solvent assisted hydrogen atom transfer. Hence, the deuteration of **6** in the C6 position is observed solely in the *N*-d_2-formamide reaction (**6b** in panel B). Moreover, the formamide molecules act as bridges for proton relay. In the photoreaction in *N*-d_2-formamide, H to D exchange results in a six-fold decrease of the tautomerization rate, resulting in a longer lifetime of the enol hydrouridyl radical **20**, which may more easily undergo radical coupling with the formamide radical **23** yielding enol **24**. After deuterium exchange between enol **24** proton and deuterium of *N*-d_2-formamide, C5 deuterated formamide adducts of uridine **26** could be formed via **25** after keto-enol tautomerization. In the reaction with 1-*d*-formamide, final abstraction of another deuterium atom from another 1-*d*-formamide molecule to the C5 position of the partially hydrogenated pyrimidine ring yields **6a**. Selective deuteration of **6a** at C5 position observed in the 1-*d*-formamide reaction corroborates the computational suggestion that the final deuterium atom abstraction yields the carbon-centered radical of formamide **19**, which could dimerize to furnish byproduct oxamide **22**. In addition to the stepwise (proton transfer following charge transfer) mechanism, a concerted proton coupled electron transfer (PCET) mechanism (panel C) can also occur and play a role in the photochemistry of uridine in formamide solution. The concerted PCET enables the hydrogen atom transfer from the amino group of formamide to the C5 atom of uridine, producing nitrogen centered formamide radical **27** and uridine radical **28**. The energetically unstable radical **27** either tautomerizes to the carbon-centered formamide radical **23** or abstracts a proton from the C5 atom of uridine radical **28**, yielding C5 deuterated uridine **29**, which could be isolated together with natural uridine **2** in an uncompleted photoreaction. Prolonged irradiation in *N*-d_2-formamide would convert **29** to 5,6-dideutero-uridine **6c**. Recombination of radicals **28** and **23** produces formamide adduct **26**. Hydrouridyl radical **28** could also abstract a hydrogen atom from *N*-d_2-formamide to give **6a**.

Supplementary Figures

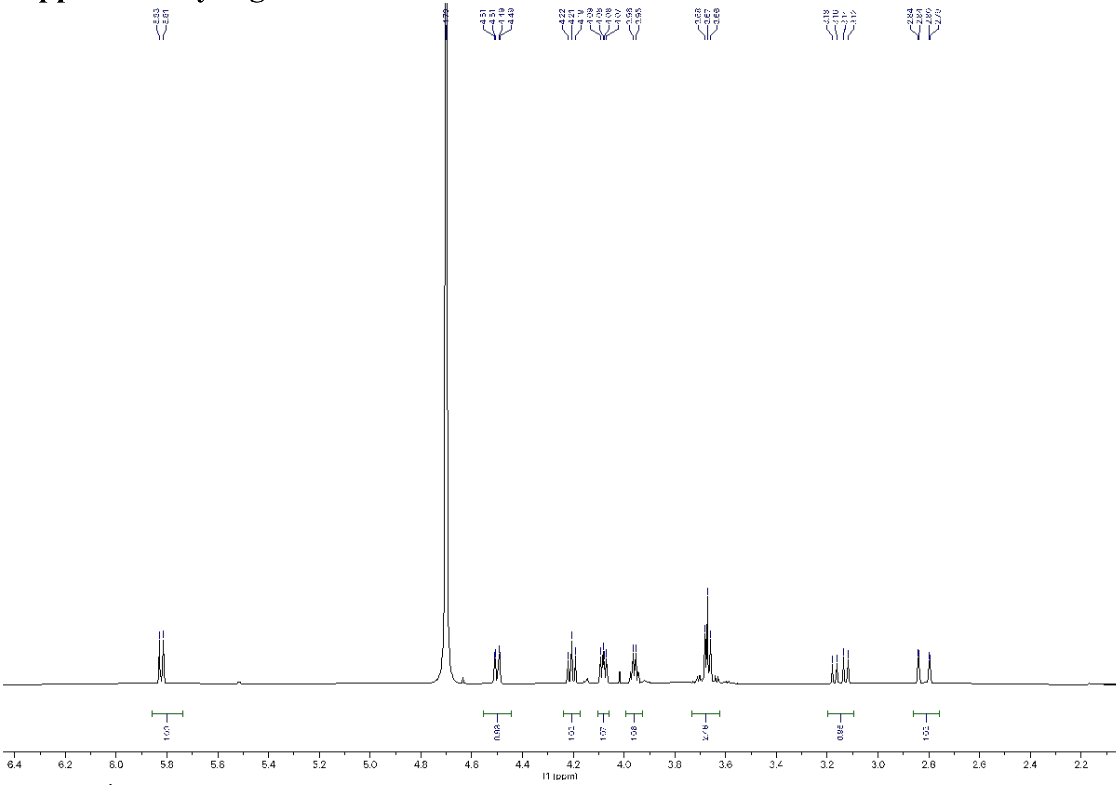

Fig. S1 ^{1}H NMR spectrum of 5, 6-dihydro-6 (*S* or *R*)-carboxamido-uridine (isomer 1) in D_2O.

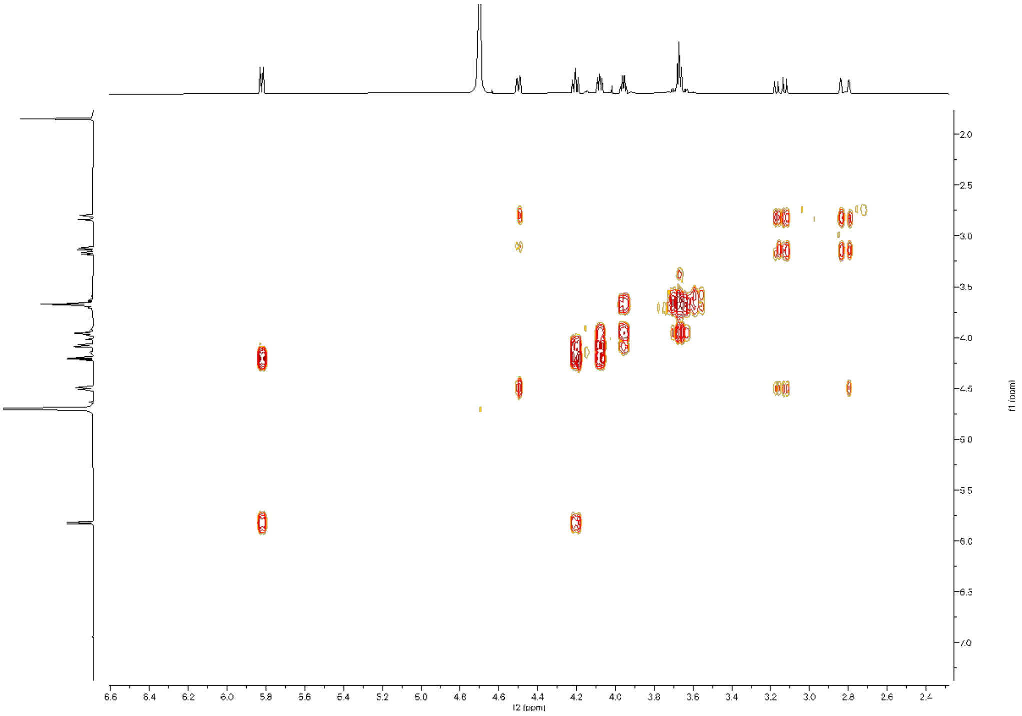

Fig. S2 COSY NMR spectrum of 5, 6-dihydro-6 (*S* or *R*)-carboxamido-uridine (isomer 1) in D_2O.

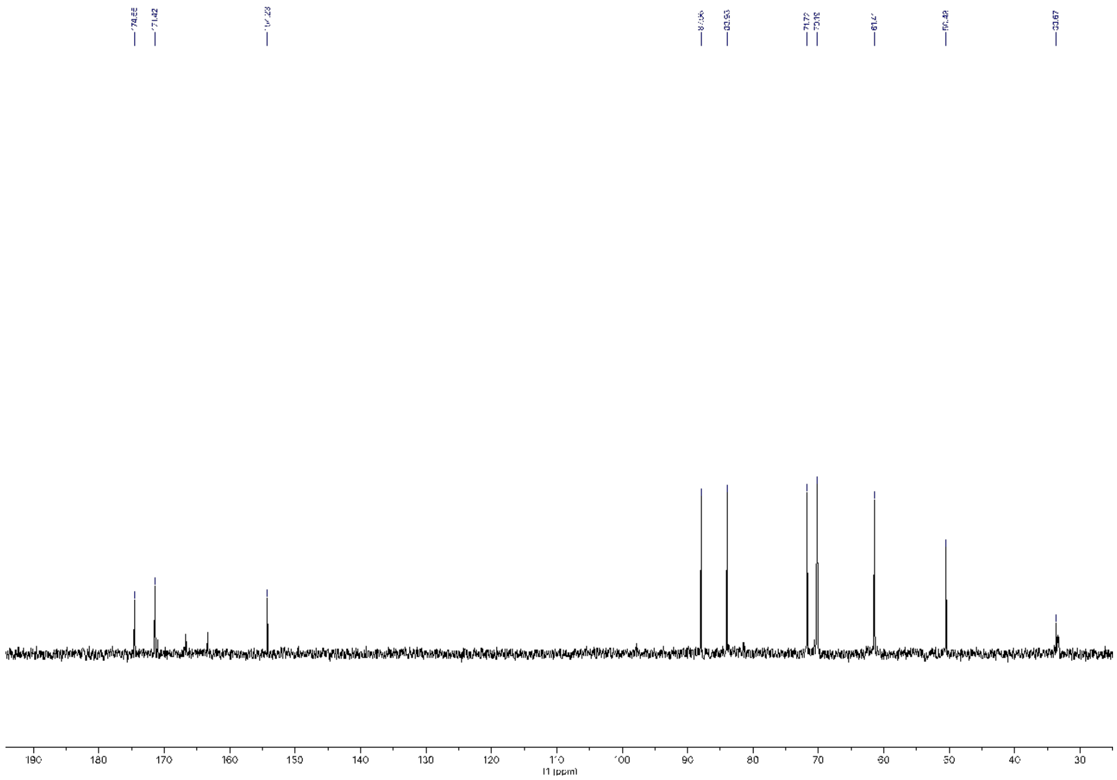

Fig. S3 ^{13}C NMR spectrum of 5, 6-dihydro-6 (*S* or *R*)-carboxamido-uridine (isomer 1) in D_2O.

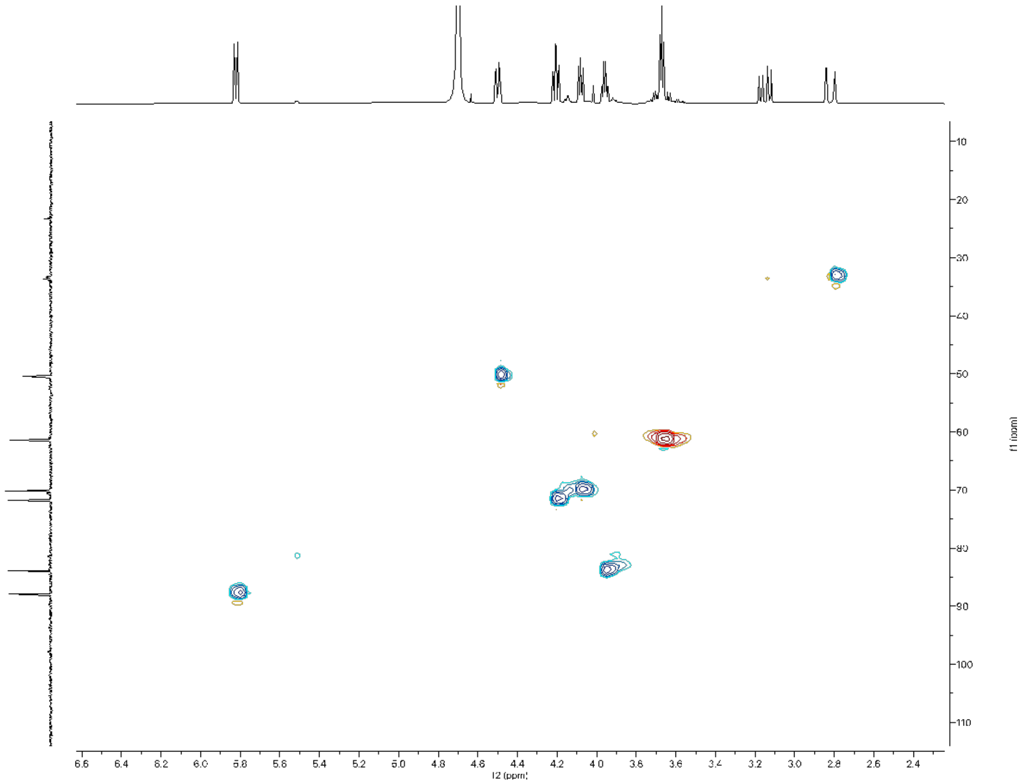

Fig. S4 HSQC spectrum of 5, 6-dihydro-6 (*S* or *R*)-carboxamido-uridine (isomer 1) in D_2O.

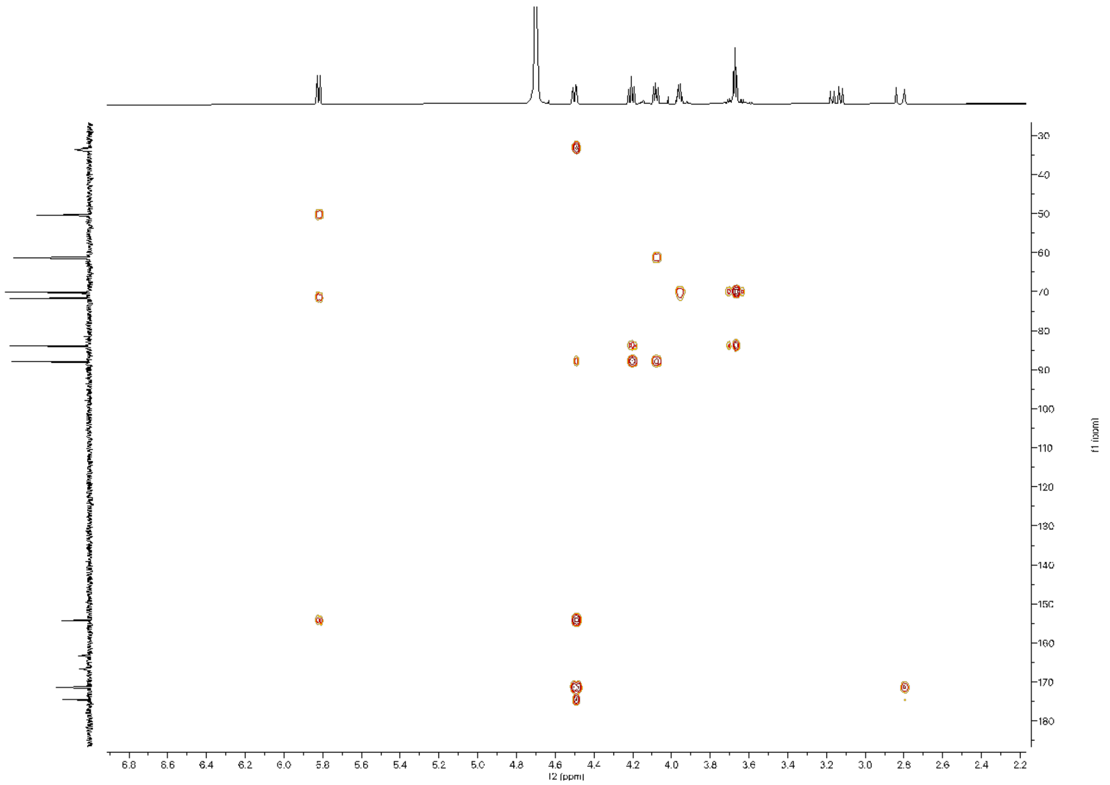

Fig. S5 HMBC spectrum of 5, 6-dihydro-6 (*S* or *R*)-carboxamido-uridine (isomer 1) in D_2O.

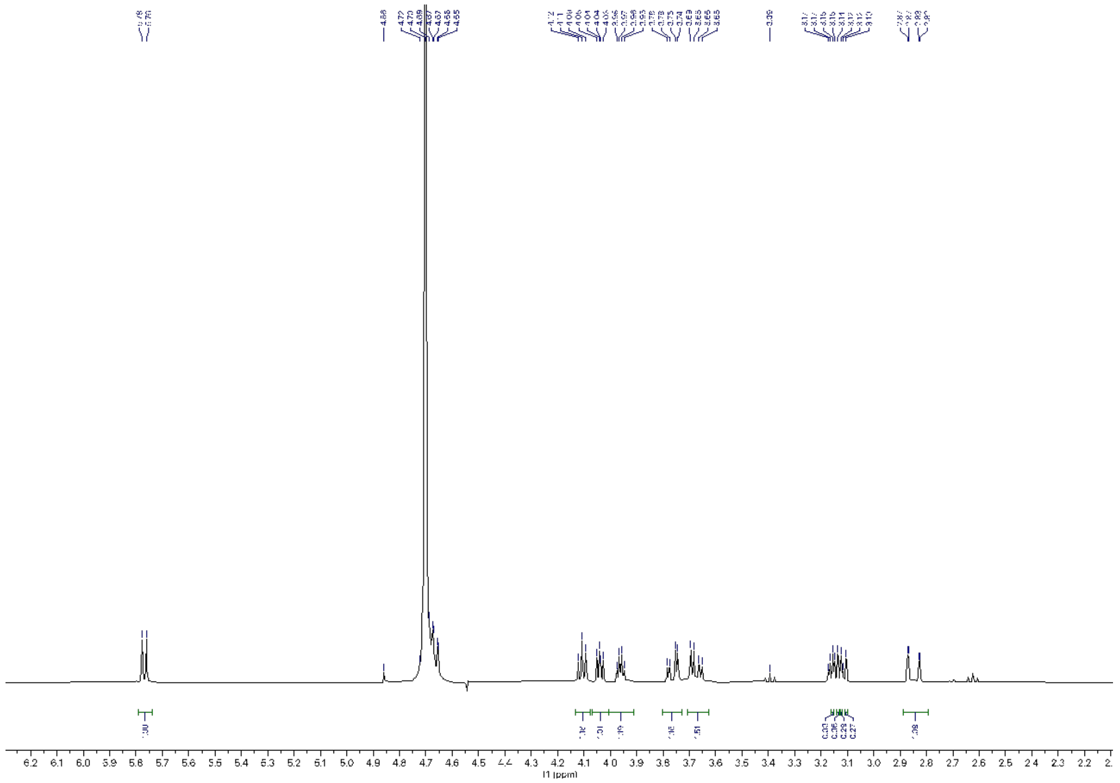

Fig. S6 ^{1}H NMR spectrum of 5, 6-dihydro-6 (*S* or *R*)-carboxamido-uridine (isomer 2) in D_2O.

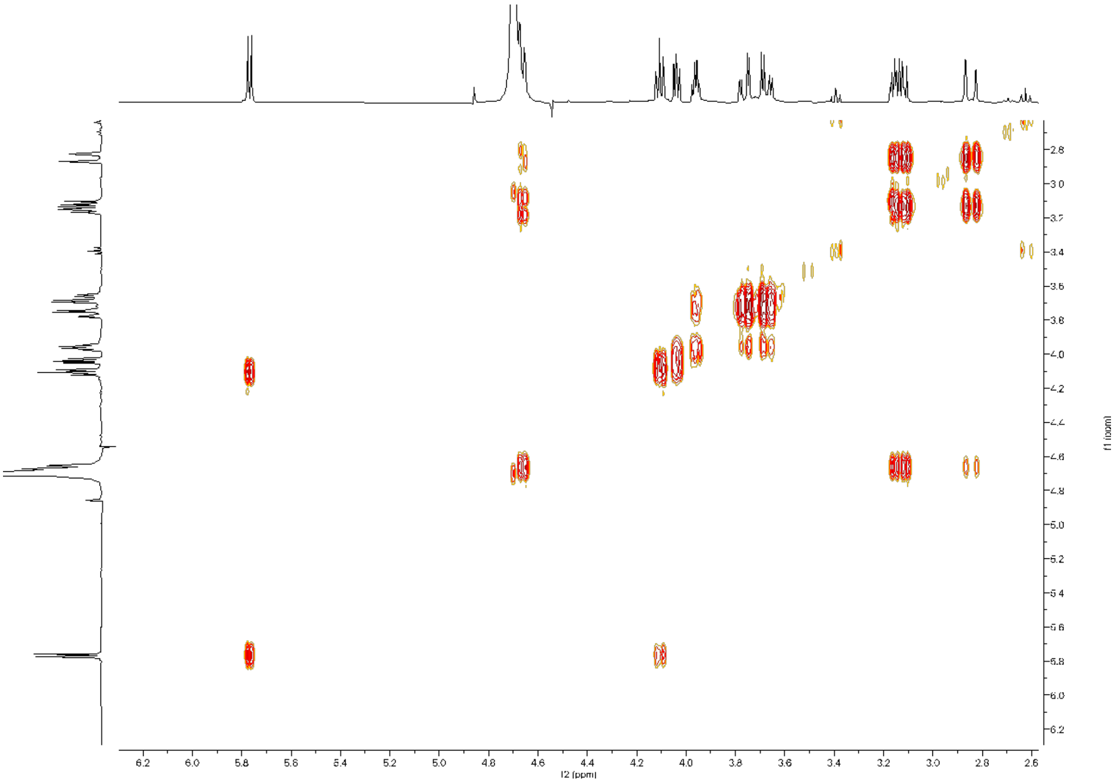

Fig. S7 COSY spectrum of 5, 6-dihydro-6 (*S* or *R*)-carboxamido-uridine (isomer 2) in D_2O.

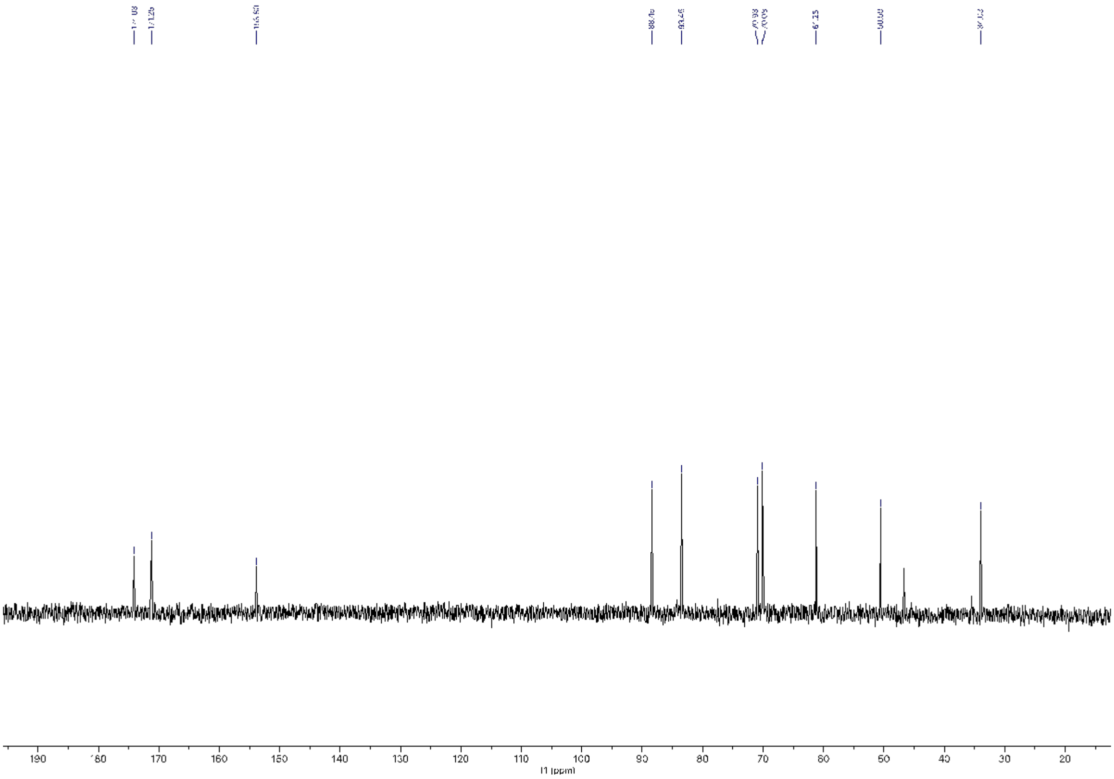

Fig. S8 ^{13}C NMR spectrum of 5, 6-dihydro-6 (*S* or *R*)-carboxamido-uridine (isomer 2) in D_2O.

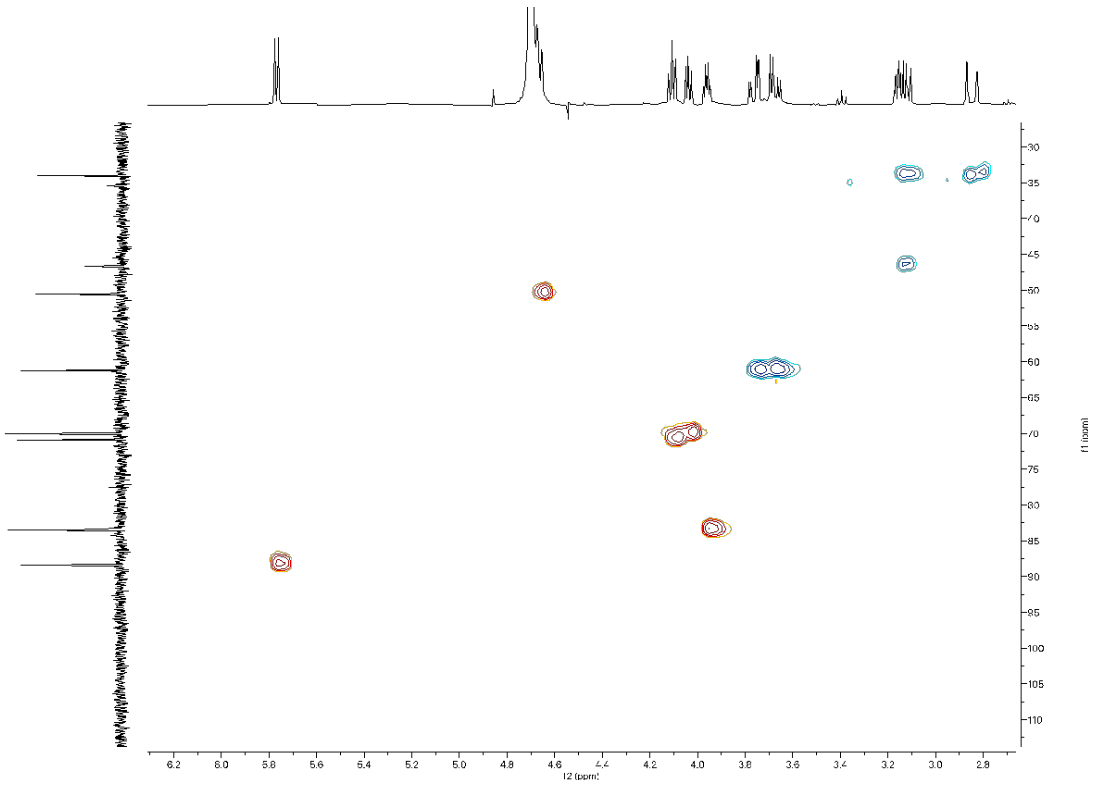

Fig. S9 HSQC NMR spectrum of 5, 6-dihydro-6 (*S* or *R*)-carboxamido-uridine (isomer 2) in D_2O.

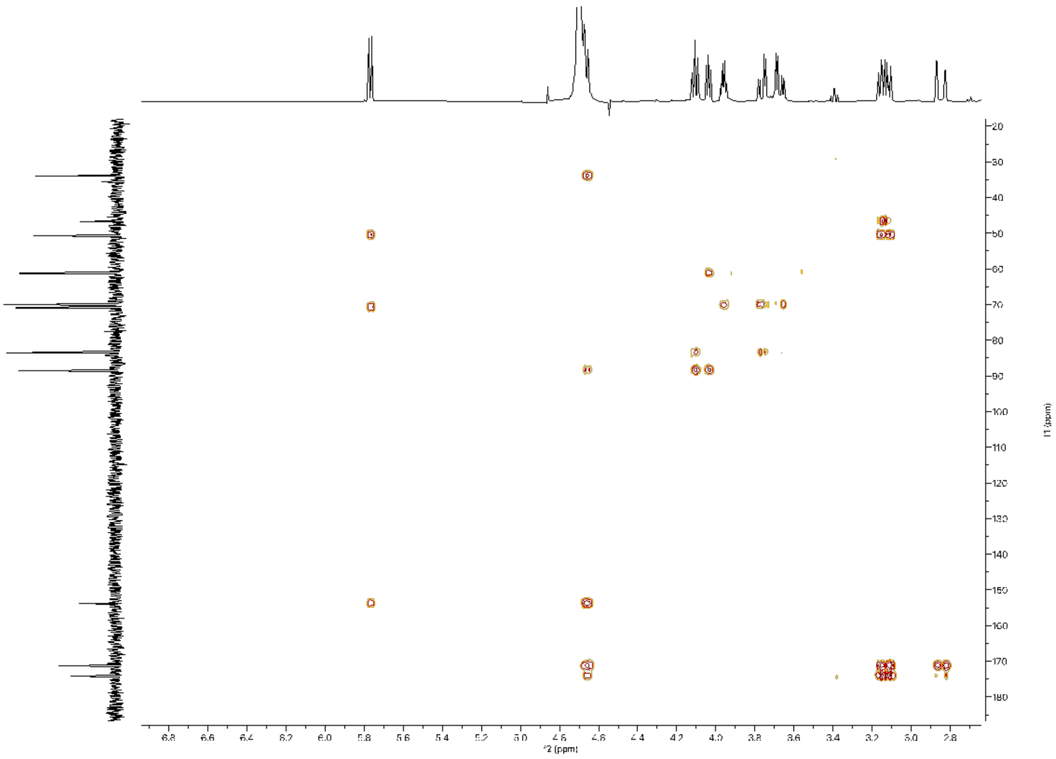

Fig. S10 HMBC NMR spectrum of 5, 6-dihydro-6 (*S* or *R*)-carboxamido-uridine (isomer 2) in D_2O.

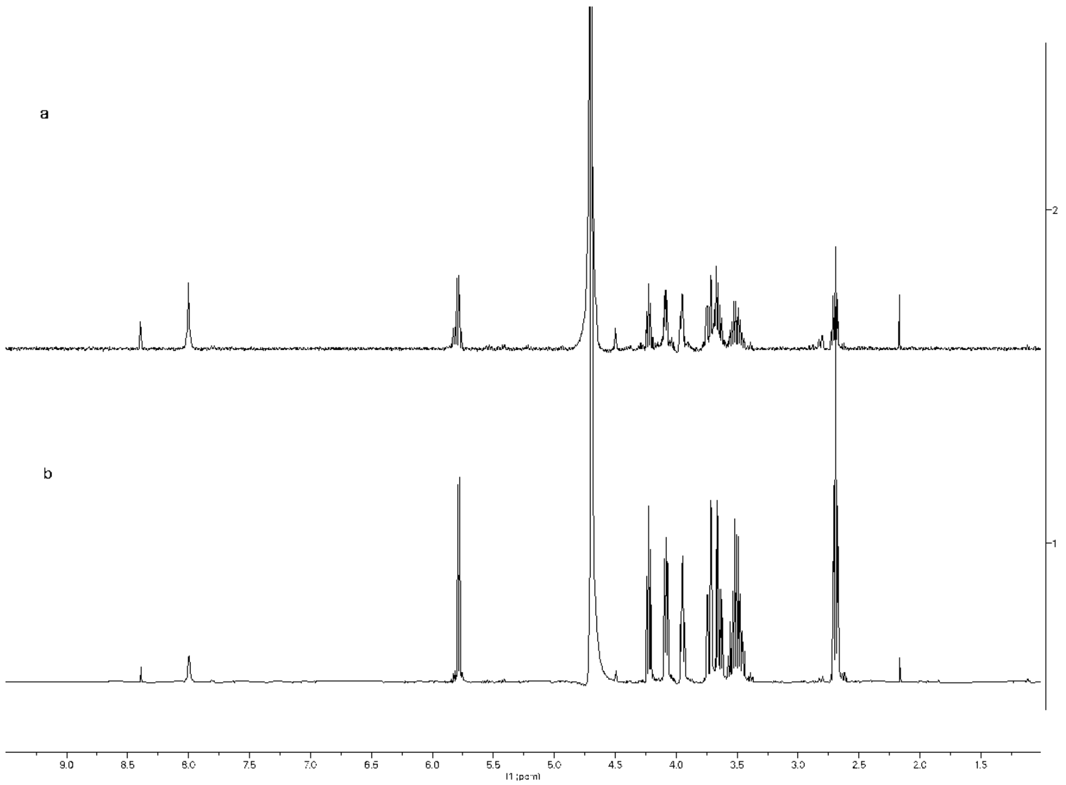

Fig. S11 ^{1}H NMR spectra of the mixture after photoreaction of uridine (60 mM) in formamide, and the spiking experiment with dihydrouridine. a) ^{1}H NMR spectrum for the reaction mixture after 7 hours of irradiation; b) as a), spiked with dihydrouridine.

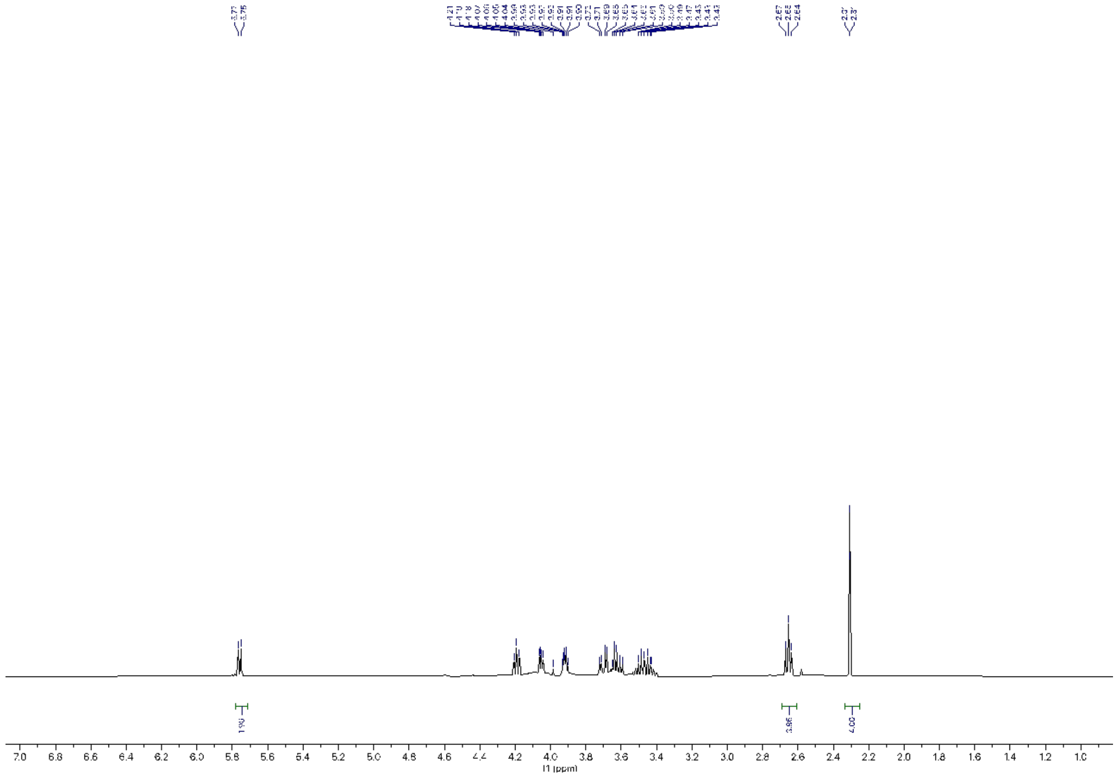

Fig. S12 ^{1}H NMR spectrum of the mixture after photoreaction of uridine (16 mM) in formamide with added sodium succinate (150 uL of 50 mM solution).

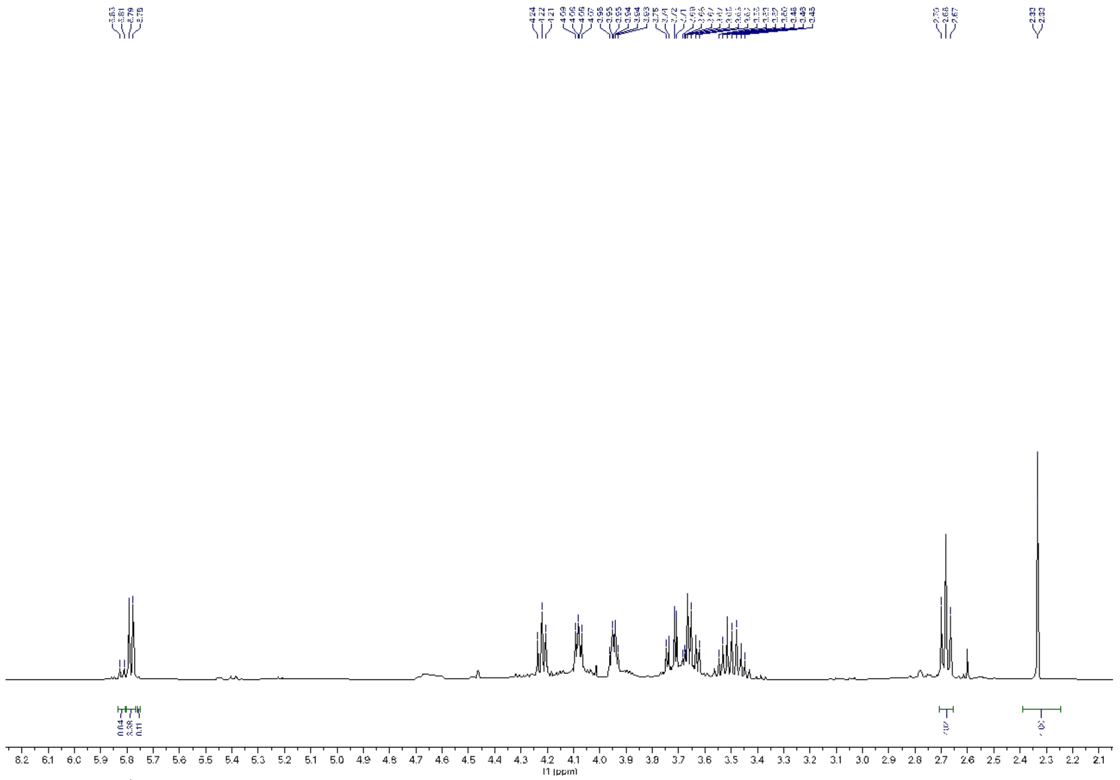

Fig. S13 ^1H NMR spectrum of the mixture after photoreaction of uridine (32 mM) in formamide with added sodium succinate (150 uL of 50 mM solution).

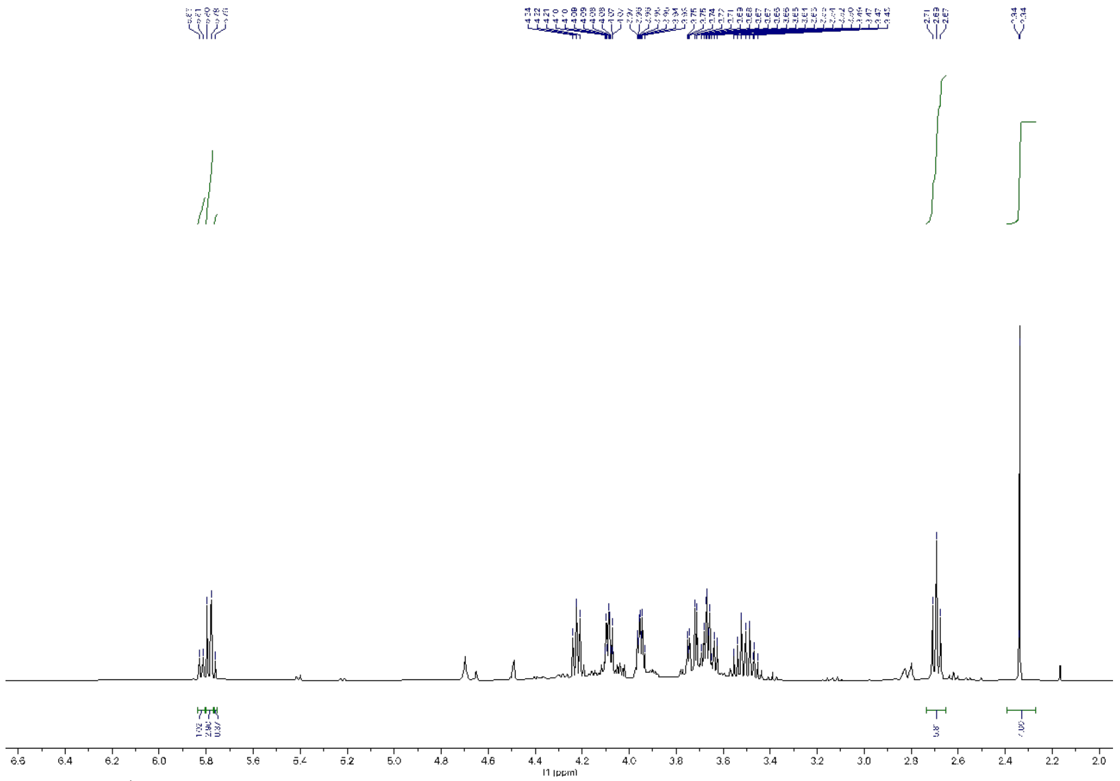

Fig. S14 ^1H NMR spectrum of the mixture after photoreaction of uridine (60 mM) in formamide with added sodium succinate (250 uL of 50 mM solution).

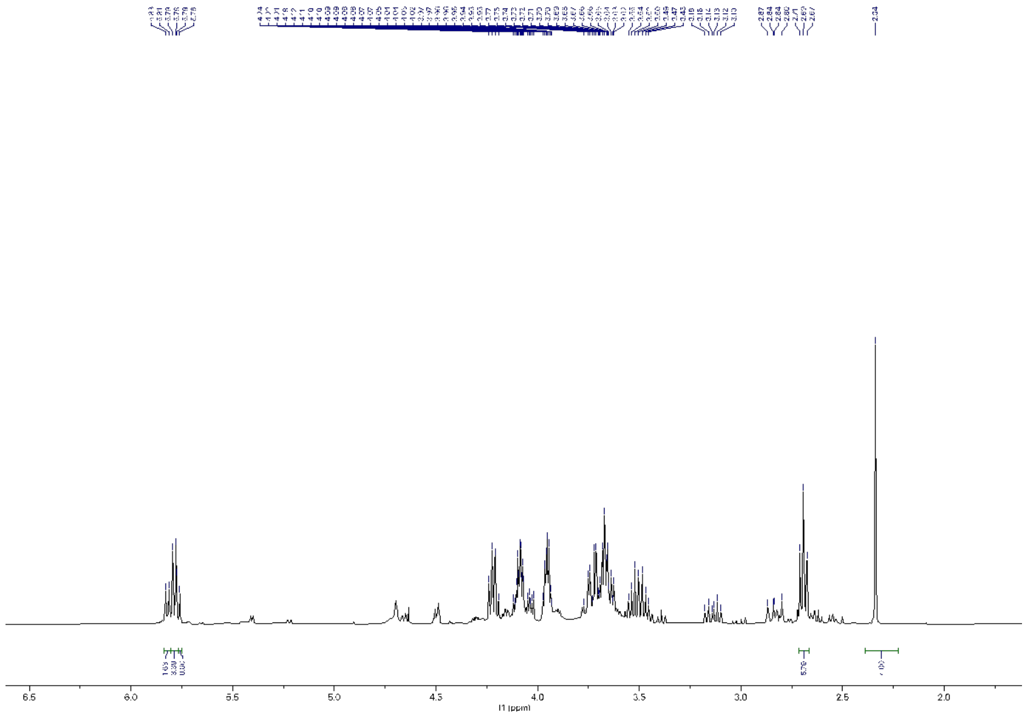

Fig. S15 ^{1}H NMR spectrum of the mixture after photoreaction of uridine (100 mM) in formamide with added sodium succinate (250 uL of 50 mM solution).

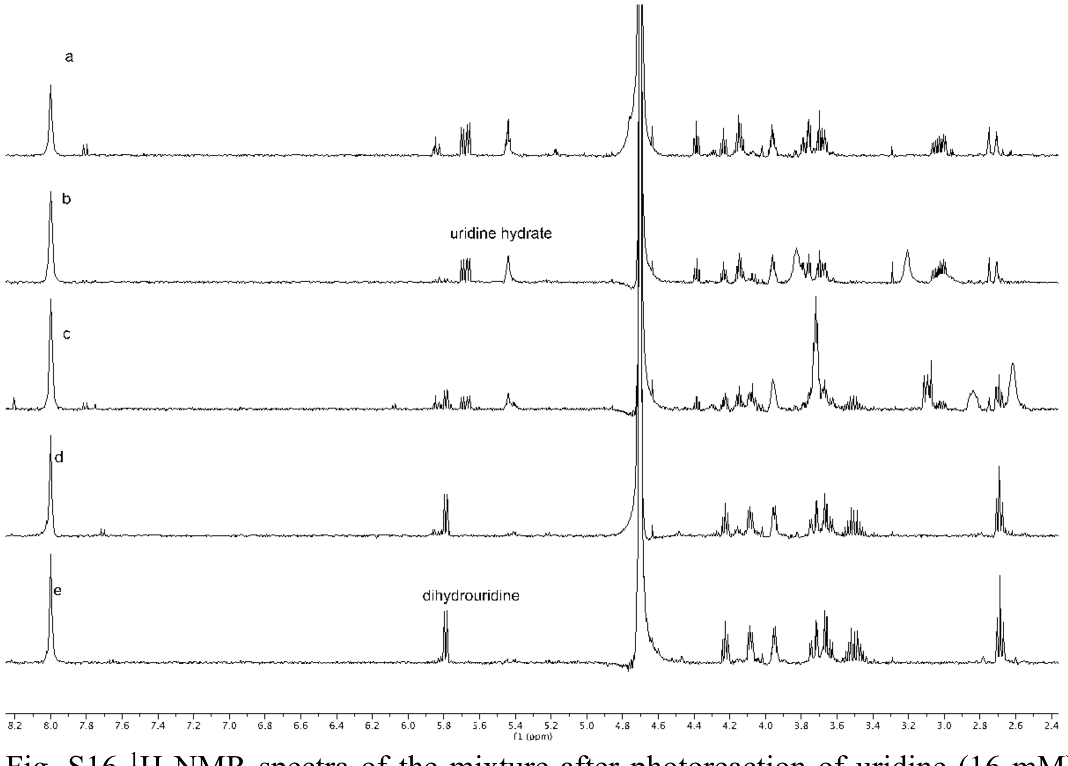

Fig. S16 ^{1}H NMR spectra of the mixture after photoreaction of uridine (16 mM) in formamide/water mixtures. a) Reaction in 10% formamide in water (containing 13 % of remaining uridine); b) Reaction in 25% formamide in water; c) Reaction in 50% formamide in water; d) Reaction in 25% water in formamide; e) Reaction in 10% water in formamide.

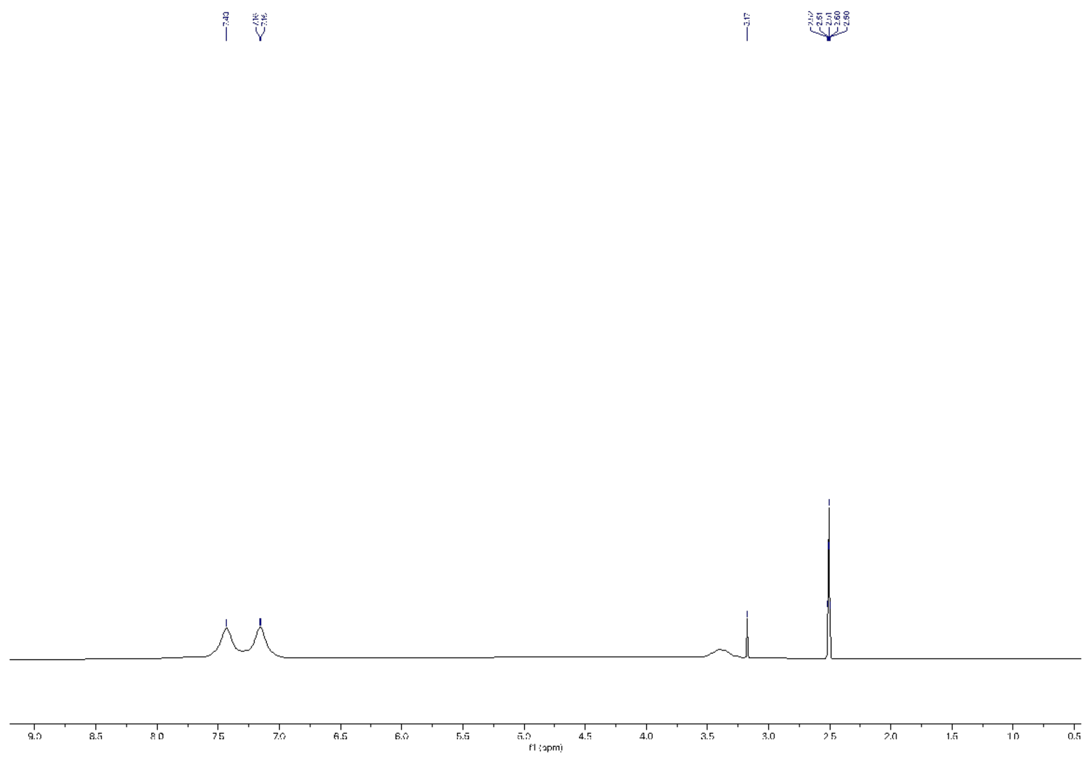

Fig. S17 ^{1}H NMR spectrum of 1-d_1-formamide in DMSO-d6.

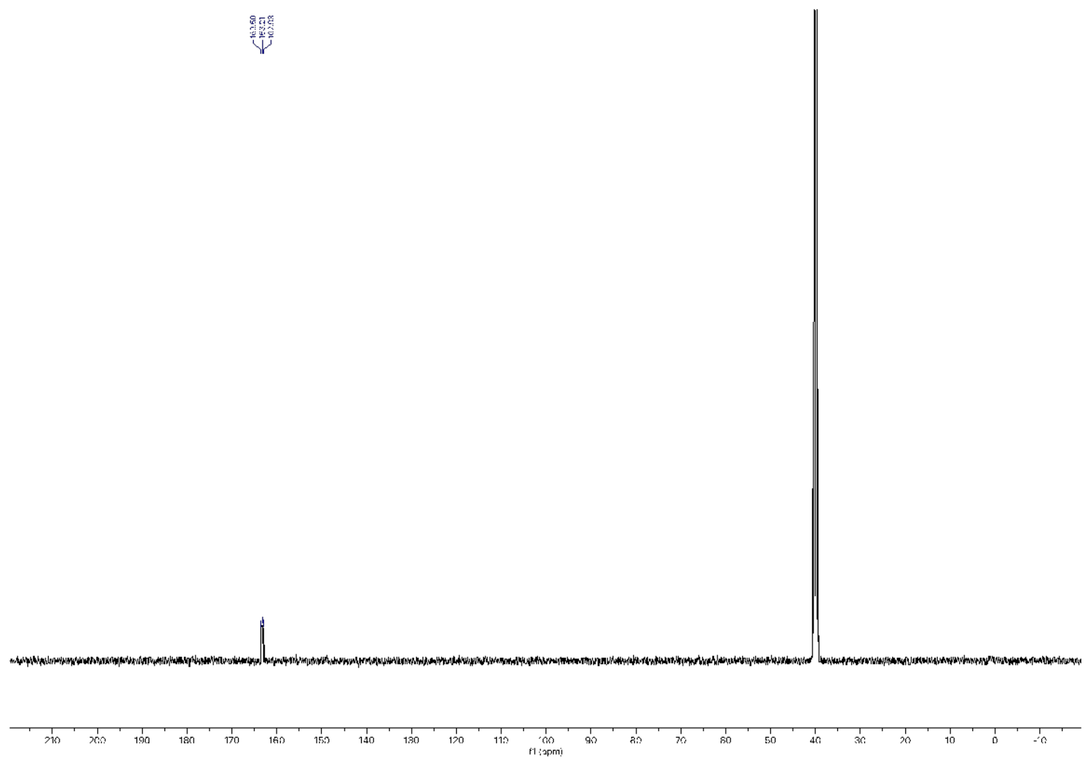

Fig. S18 ^{13}C NMR spectrum of 1-d_1-formamide in DMSO-d6.

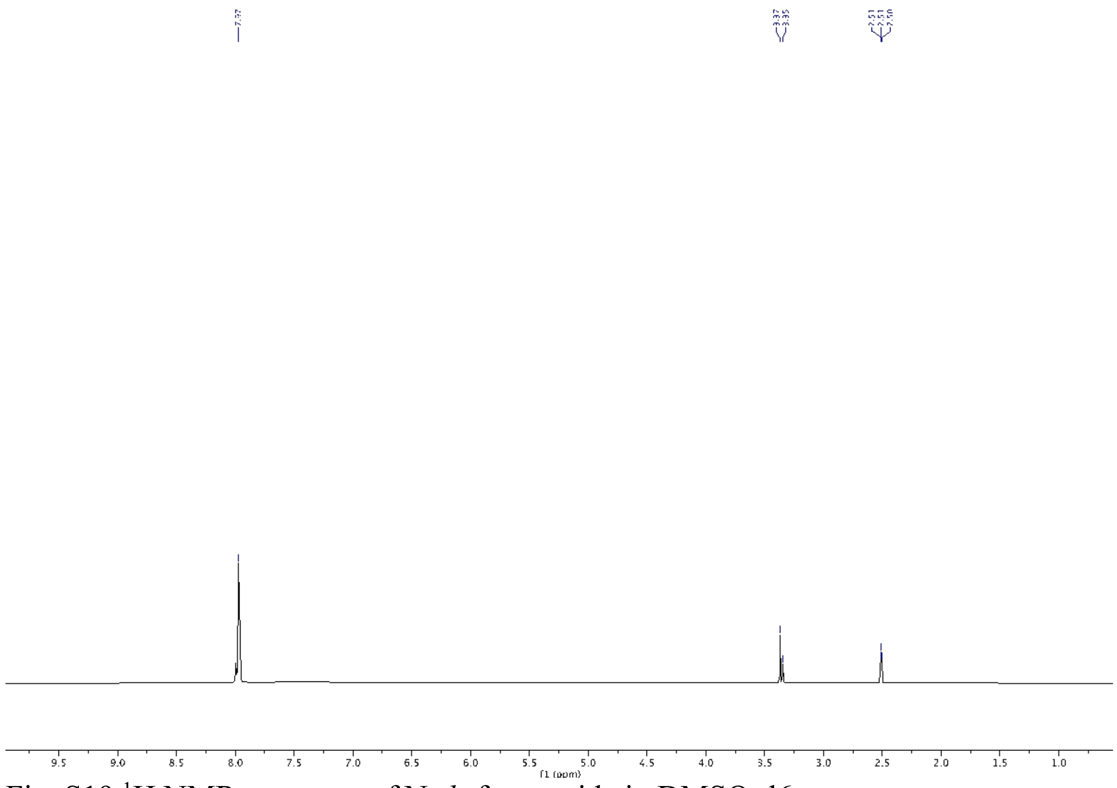

Fig. S19 ^{1}H NMR spectrum of N-d_2-formamide in DMSO-d6.

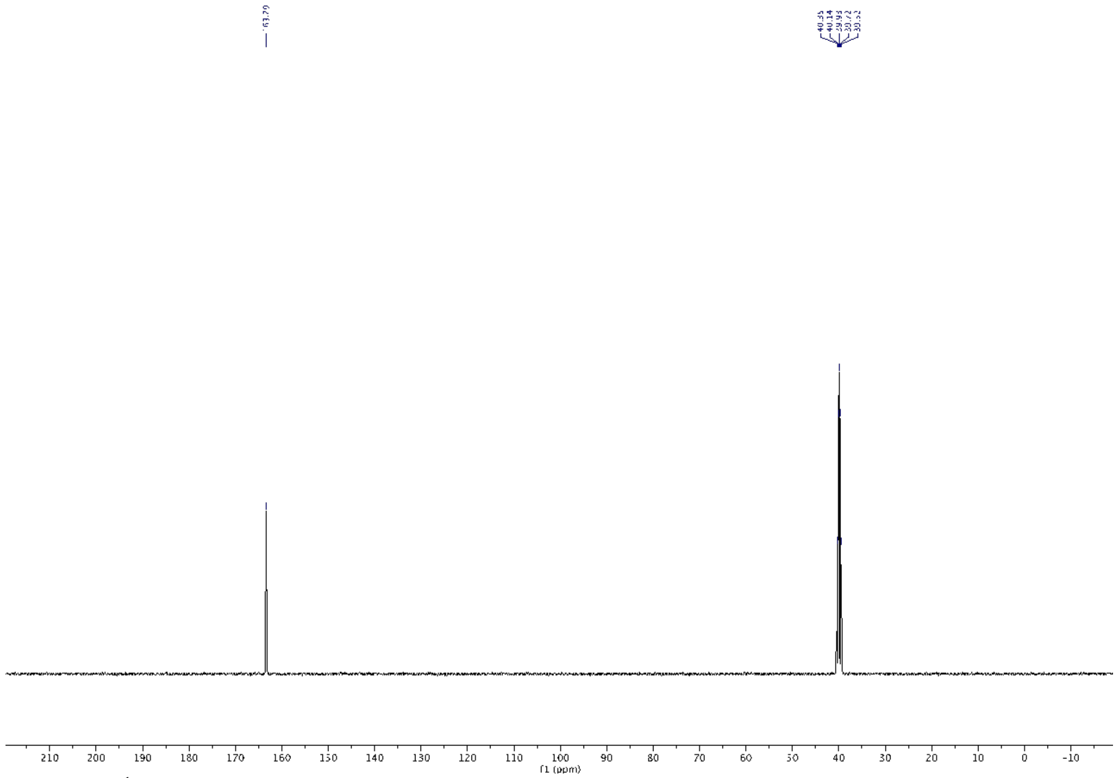

Fig. S20 ^{1}H NMR spectrum of N-d_2-formamide in DMSO-d6.

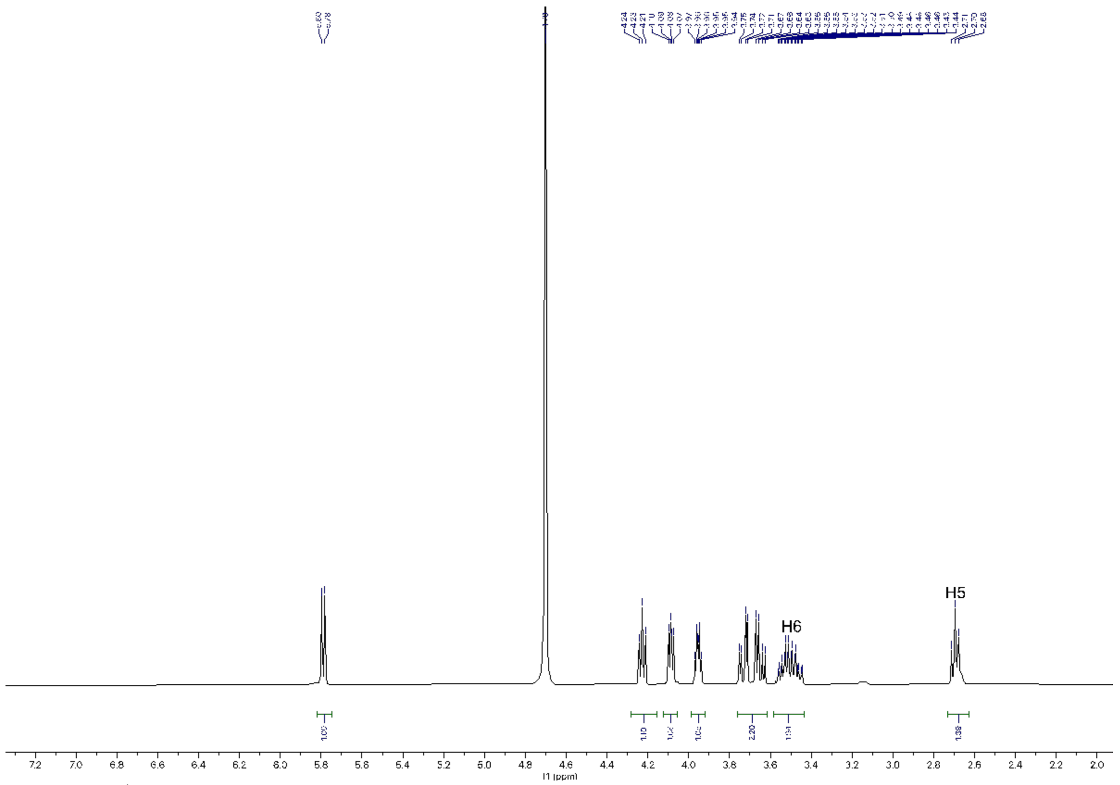

Fig. S21 ^{1}H NMR spectrum of dihydrouridine in D_2O, prepared from the reaction with 1-d_1-formamide.

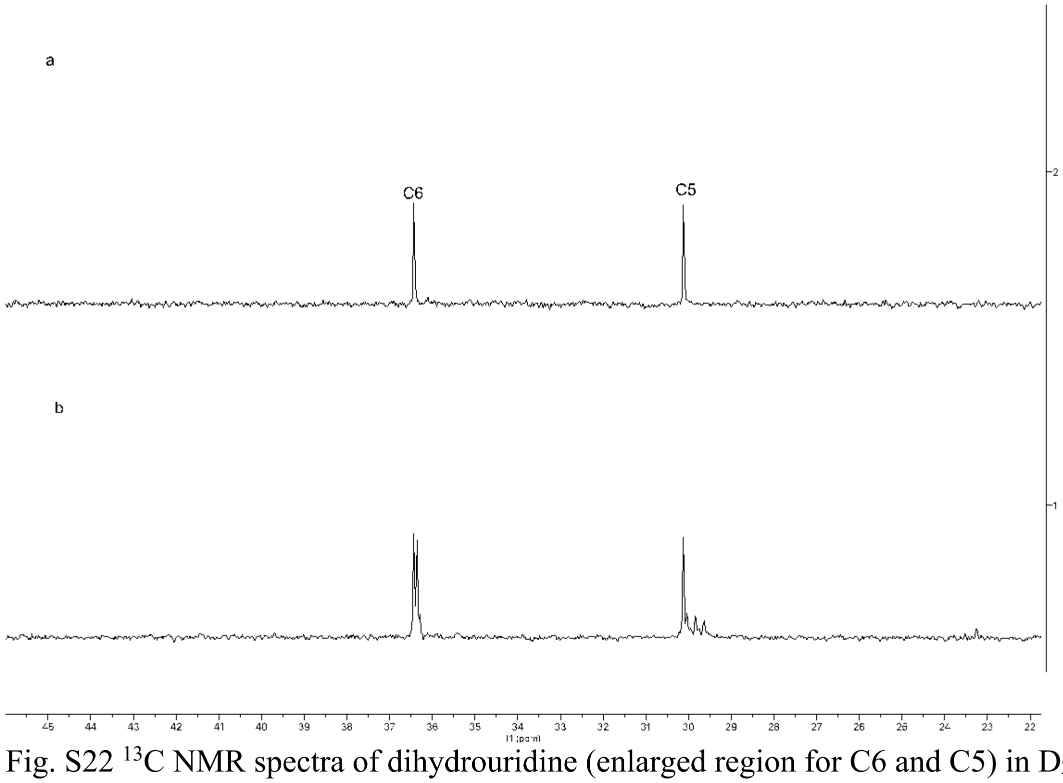

Fig. S22 ^{13}C NMR spectra of dihydrouridine (enlarged region for C6 and C5) in D_2O. a) DHU standard; b) DHU produced from reaction with 1-d_1-formamide.

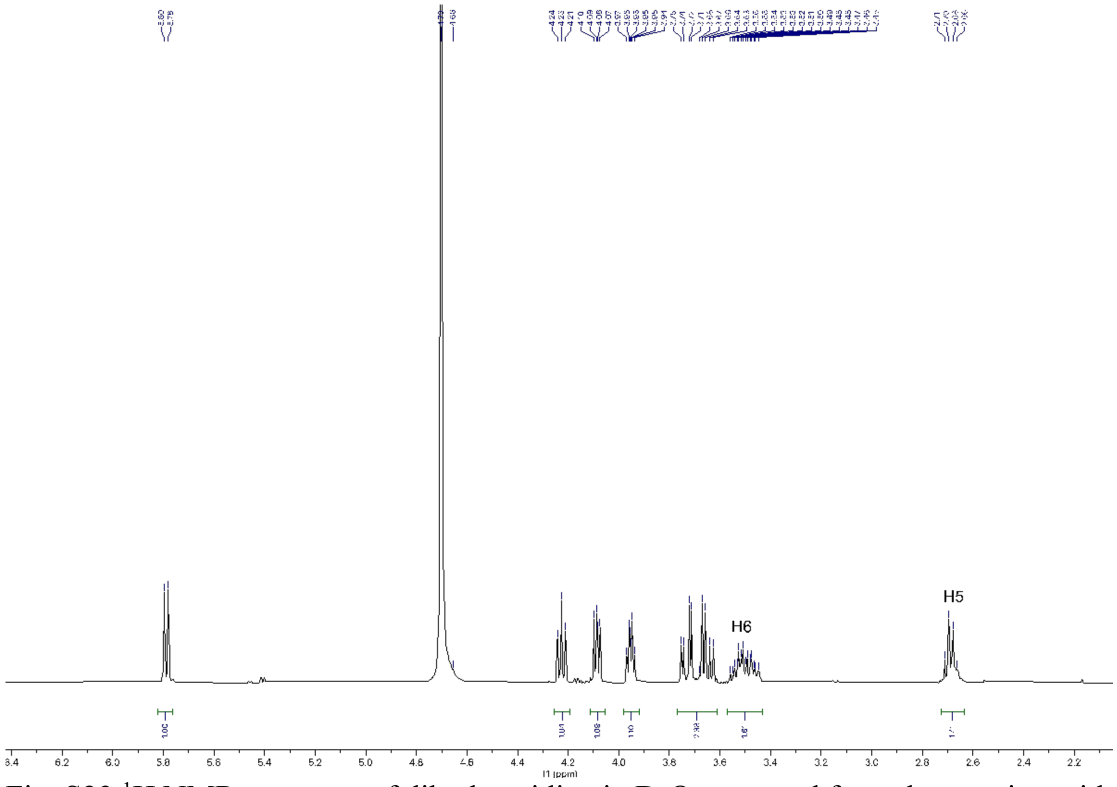

Fig. S23 ^{1}H NMR spectrum of dihydrouridine in D_2O, prepared from the reaction with N-d_2-formamide.

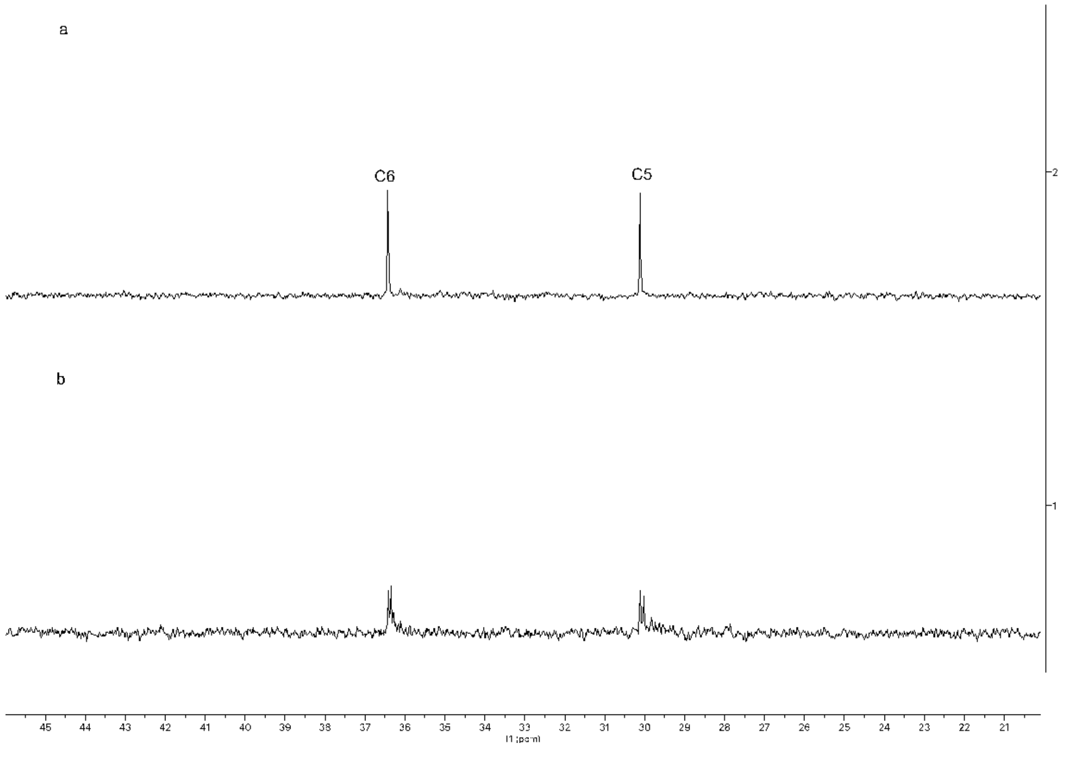

Fig. S24 ^{13}C NMR spectra of dihydrouridine (enlarged region for C6 and C5) in D_2O. a) DHU standard; b) DHU produced from reaction with N-d_2-formamide.

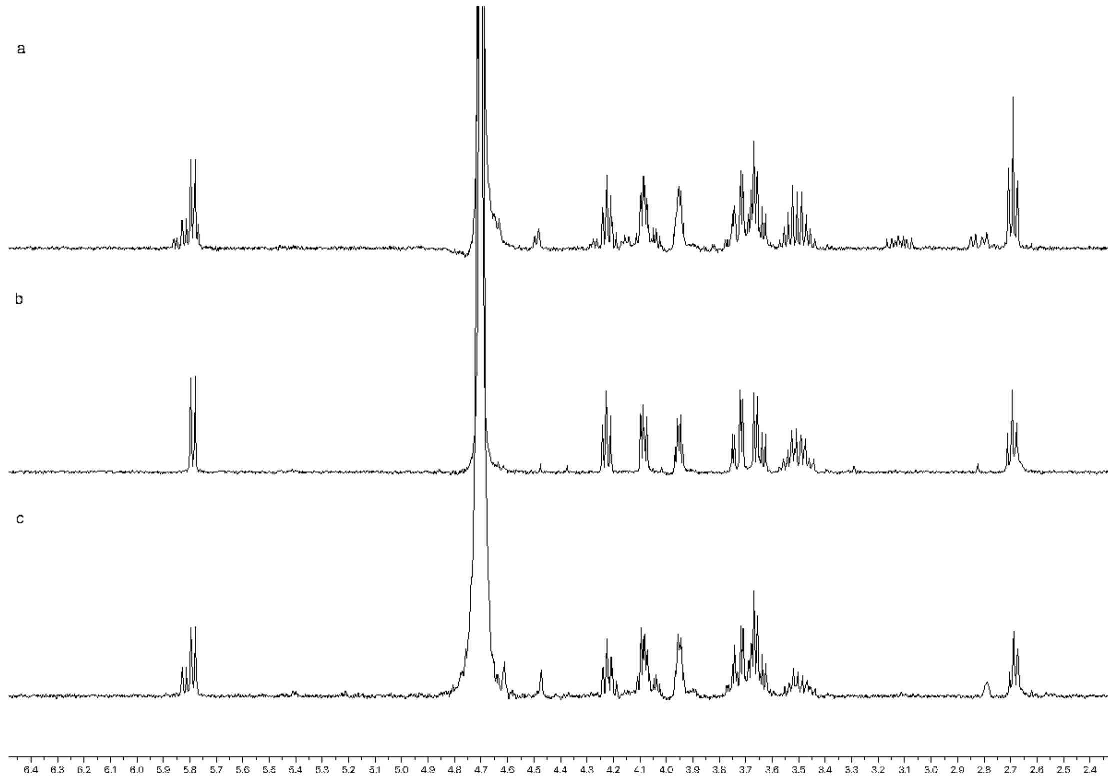

Fig. S25 ^{1}H NMR spectra of the crude mixture after irradiation of uridine (60 mM) in formamide or deuterated formamide. a) In normal formamide; b) in 1-d_1-formamide; c) in N-d_2-formamide.

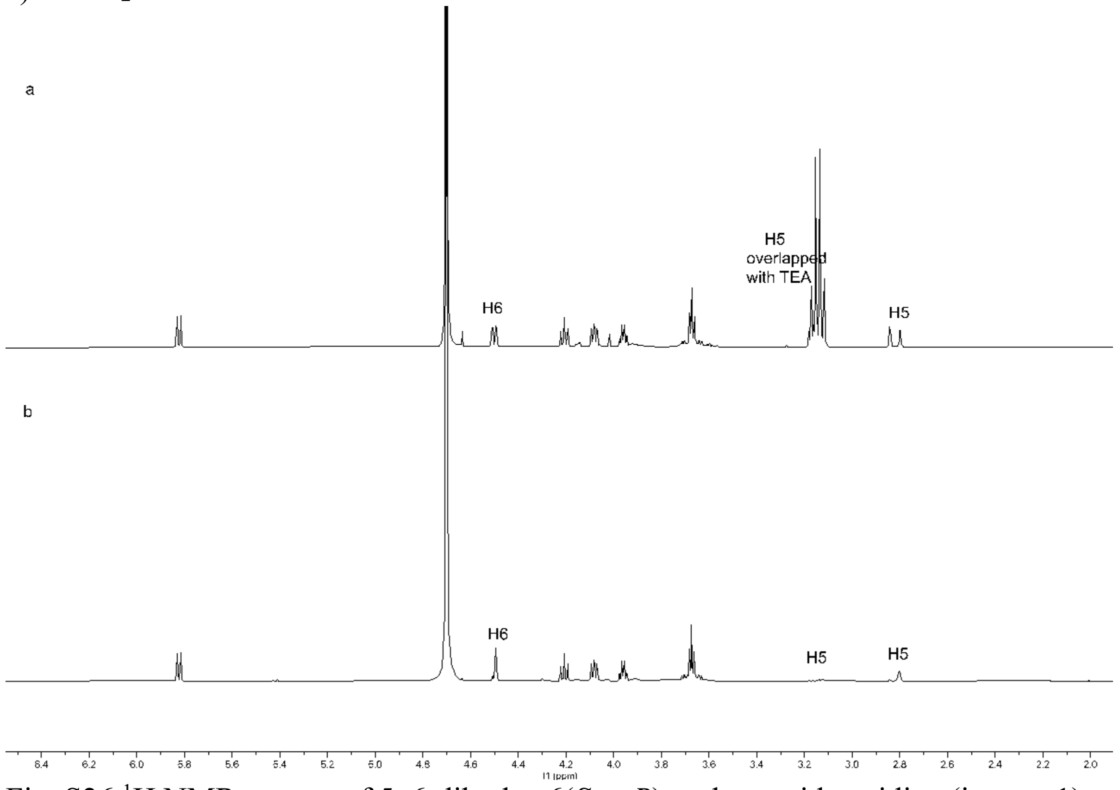

Fig. S26 ^{1}H NMR spectra of 5, 6-dihydro-6(*S* or *R*)-carboxamido-uridine (isomer 1). a) obtained from the reaction in normal formamide (peaks for H5 are overlapped with triethylamine's); b) obtained from the reaction in N-d_2-formamide.

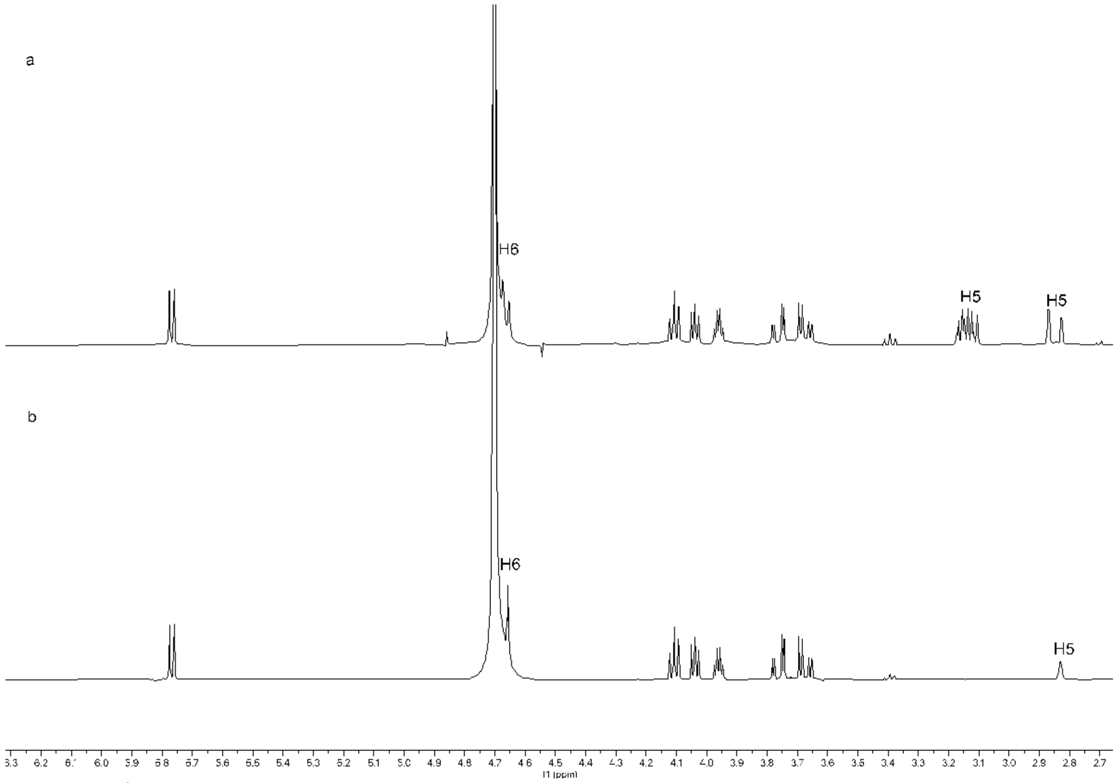

Fig. S27 ^{1}H NMR spectra of 5, 6-dihydro-6(*S* or *R*)-carboxamido-uridine (isomer 2). a) obtained from the reaction in normal formamide; b) obtained from the reaction in N-d_2-formamide.

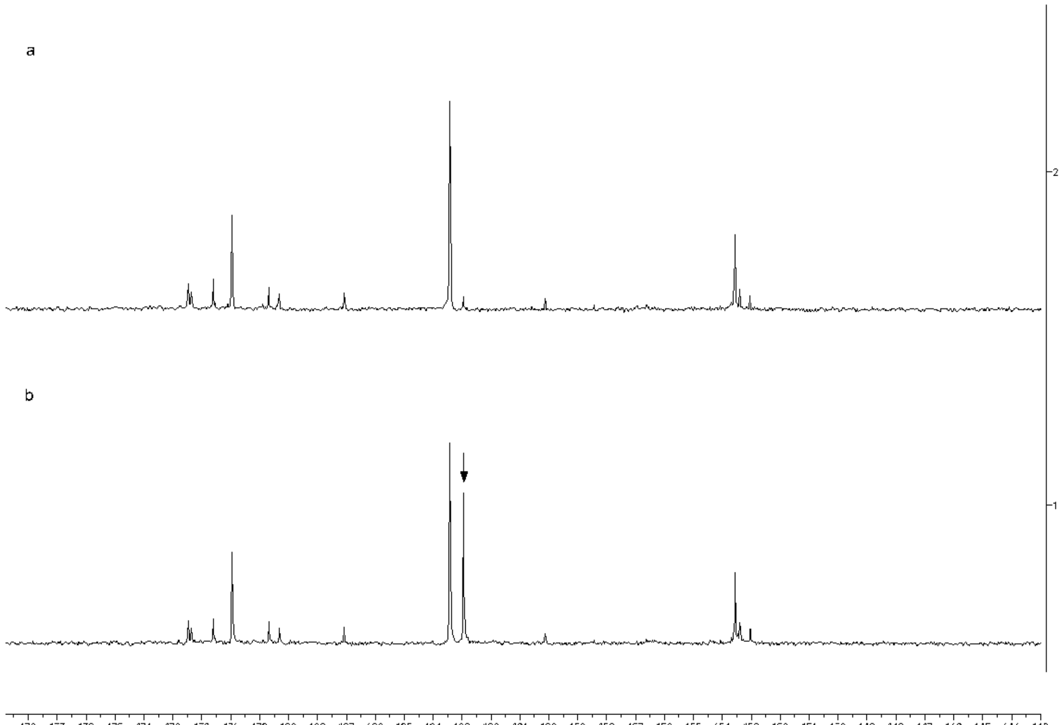

Fig. S28 ^{13}C NMR spectrum of the mixture after photochemical reaction of uridine in formamide (enlarged high field region) and the spiking spectrum with oxamide. a) ^{13}C NMR spectra of the reaction mixture in DMSO-d_6; b) as a), spiked with oxamide.

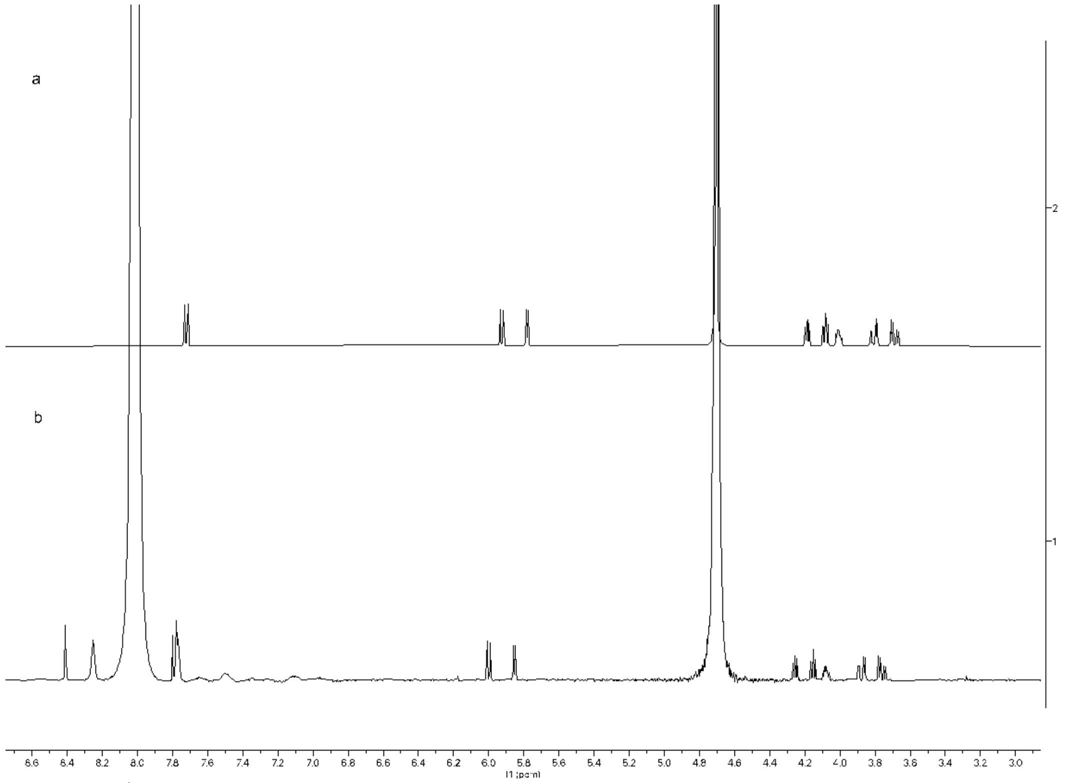

Fig. S29 ^{1}H NMR spectra of cytidine and the mixture after photoreaction of cytidine (16 mM) in formamide. a) ^{1}H NMR spectrum of cytidine in D_2O; b) ^{1}H NMR spectrum of cytidine photoreaction in formamide.

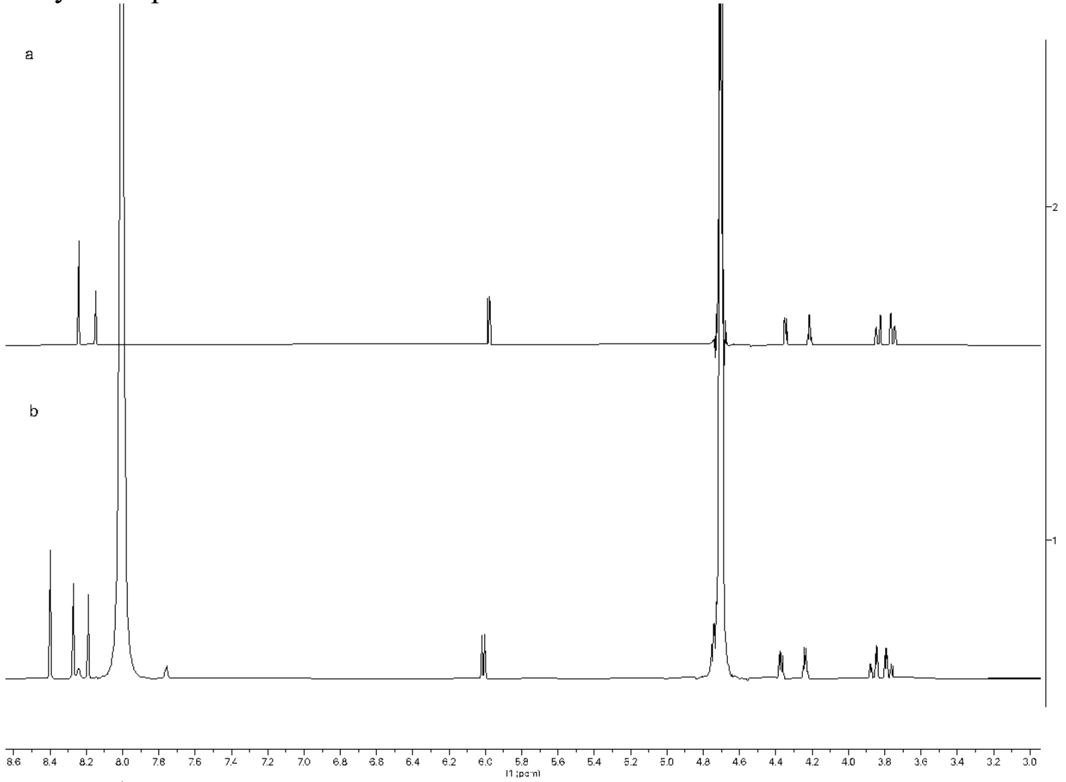

Fig. S30 ^{1}H NMR spectra of adenosine and the mixture after photoreaction of adenosine (16 mM) in formamide. a) ^{1}H NMR spectrum of adenosine in D_2O; b) ^{1}H NMR spectrum of adenosine photoreaction in formamide.

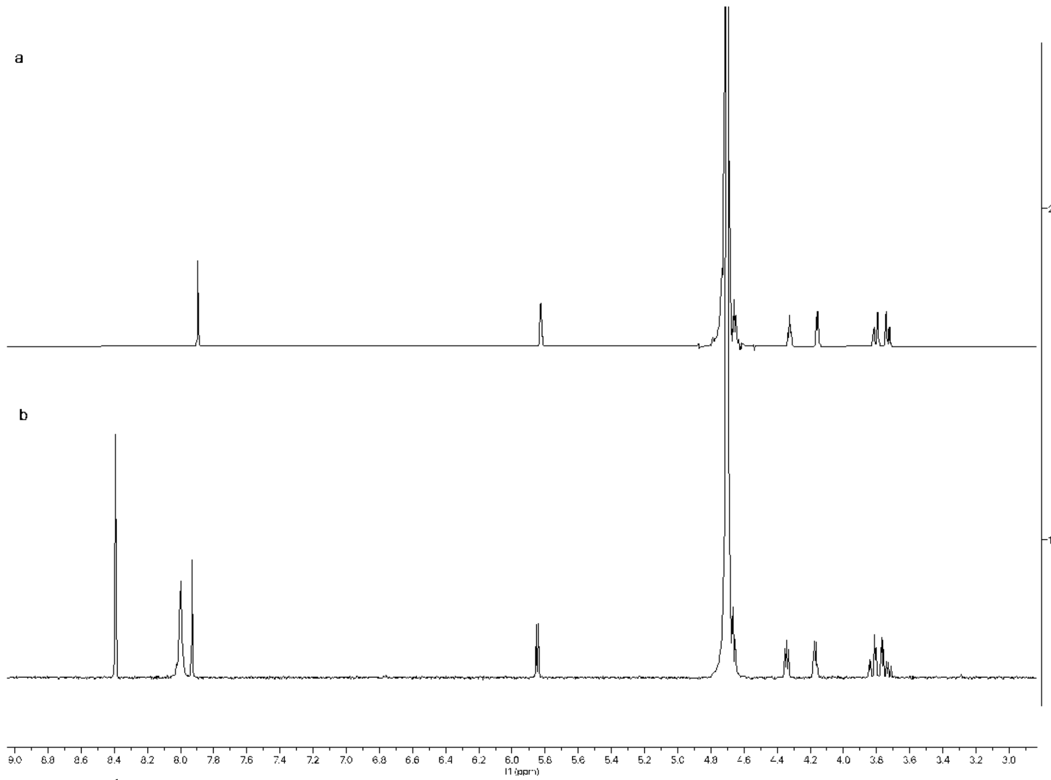

Fig. S31 ^{1}H NMR spectra of adenosine and the mixture after photoreaction of guanosine (16 mM) in formamide. a) ^{1}H NMR spectrum of guanosine in D_2O; b) ^{1}H NMR spectrum of guanosine photoreaction in formamide.

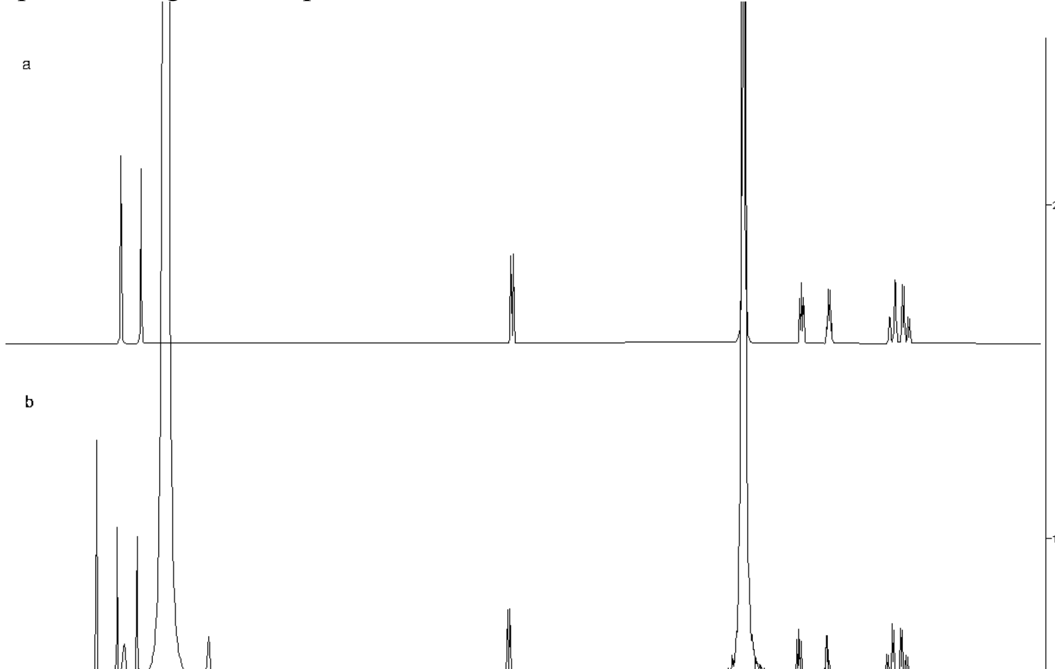

Fig. S32 ^{1}H NMR spectra of inosine and the mixture after photoreaction of inosine (16 mM) in formamide. a) ^{1}H NMR spectrum of inosine in D_2O; b) ^{1}H NMR spectrum of inosine photoreaction in formamide.

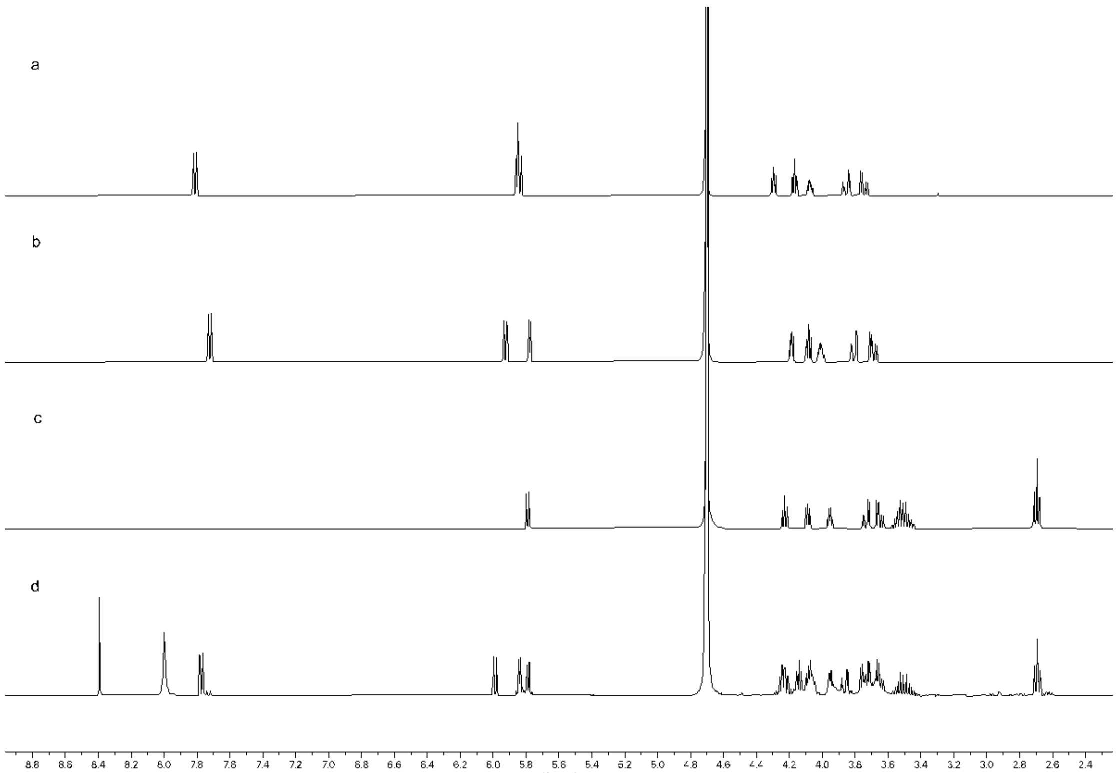

Fig. S33 ^{1}H NMR spectra of cytidine and uridine, photoreaction of the mixture (16 mM each) in formamide. a) ^{1}H NMR spectrum of uridine in D_2O; b) ^{1}H NMR spectrum of cytidine in D_2O; c) ^{1}H NMR spectrum of dihydrouridine in D_2O; d) ^{1}H NMR spectrum of photoreaction of mixtures of uridine and cytidine in formamide.

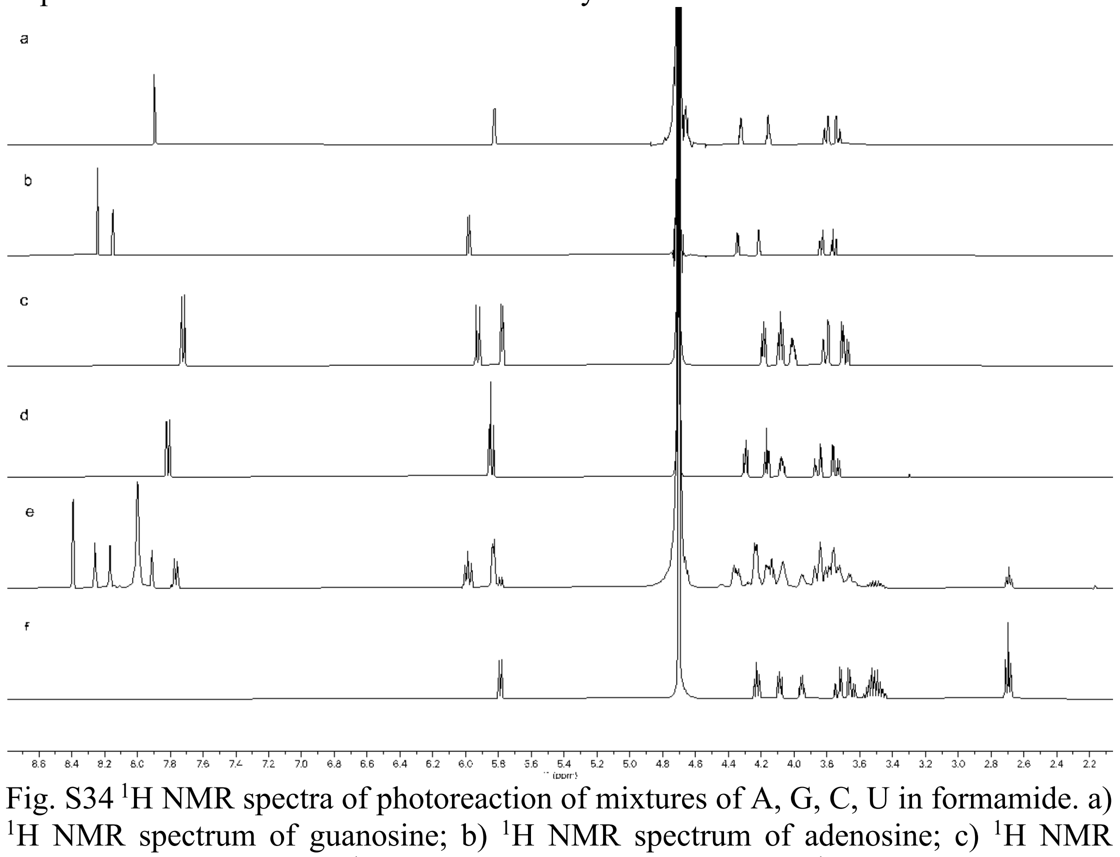

Fig. S34 ^{1}H NMR spectra of photoreaction of mixtures of A, G, C, U in formamide. a) ^{1}H NMR spectrum of guanosine; b) ^{1}H NMR spectrum of adenosine; c) ^{1}H NMR spectrum of cytidine; d) ^{1}H NMR spectrum of uridine; e) ^{1}H NMR spectrum of photoreaction of mixture in formamide; f) ^{1}H NMR spectrum of dihydrouridine.

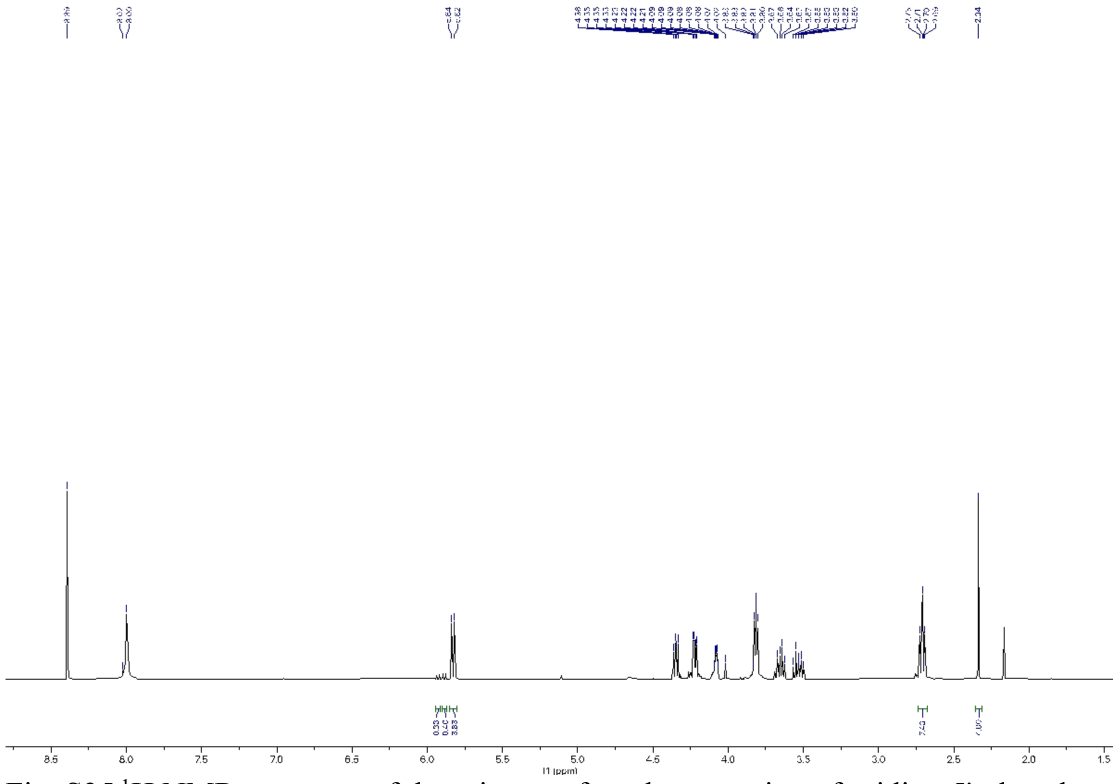

Fig. S35 ^{1}H NMR spectrum of the mixture after photoreaction of uridine-5'-phosphate in formamide with added sodium succinate (60 uL of 50 mM solution).

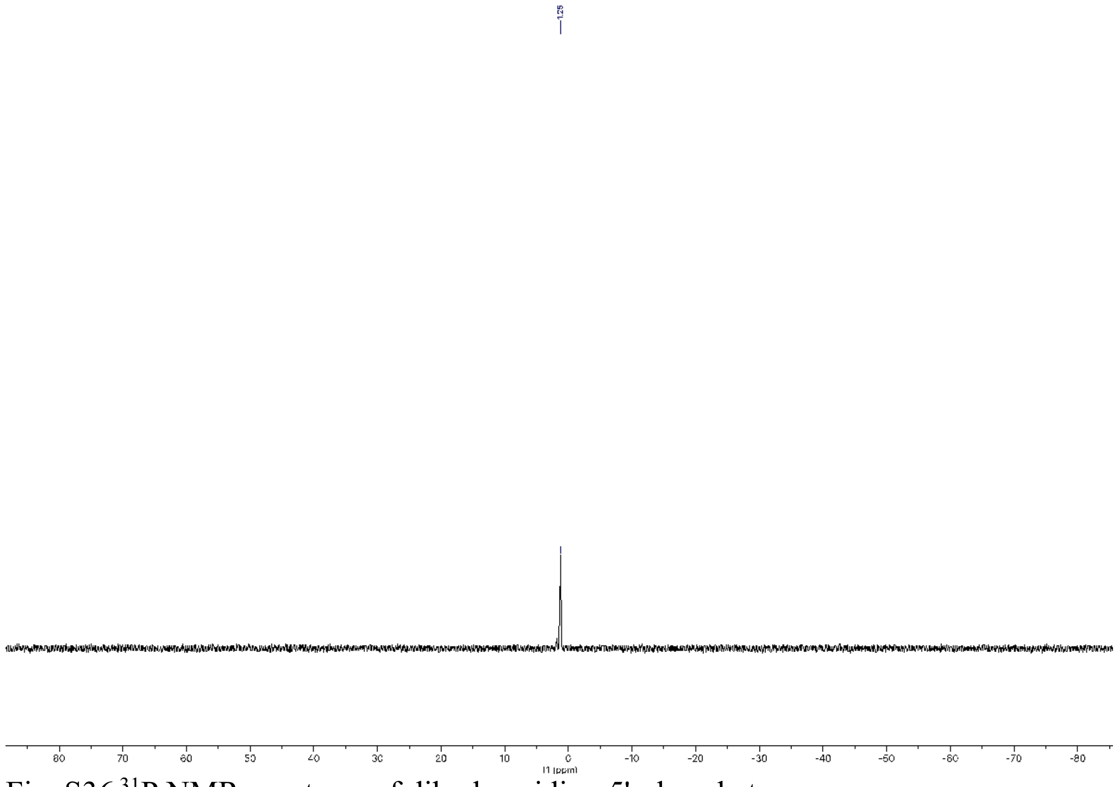

Fig. S36 ^{31}P NMR spectrum of dihydrouridine-5'-phosphate.

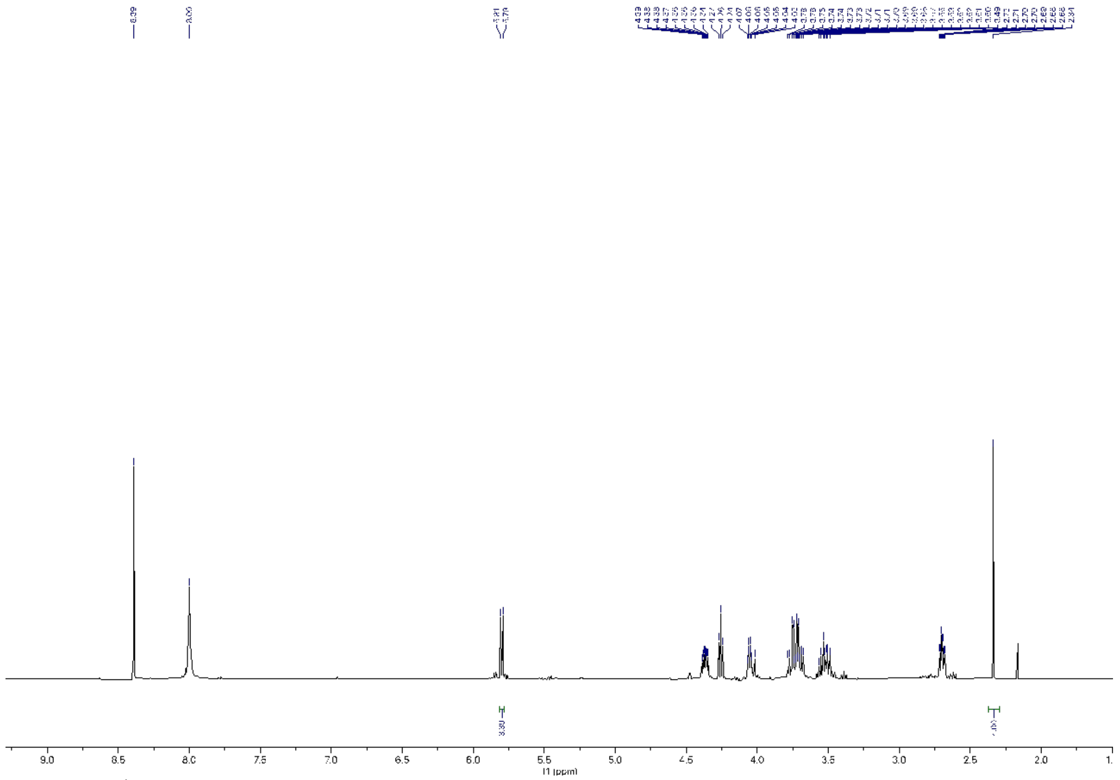

Fig. S37 ^{1}H NMR spectrum of the mixture after photoreaction of uridine-3'-phosphate in formamide with added sodium succinate (60 uL of 50 mM solution).

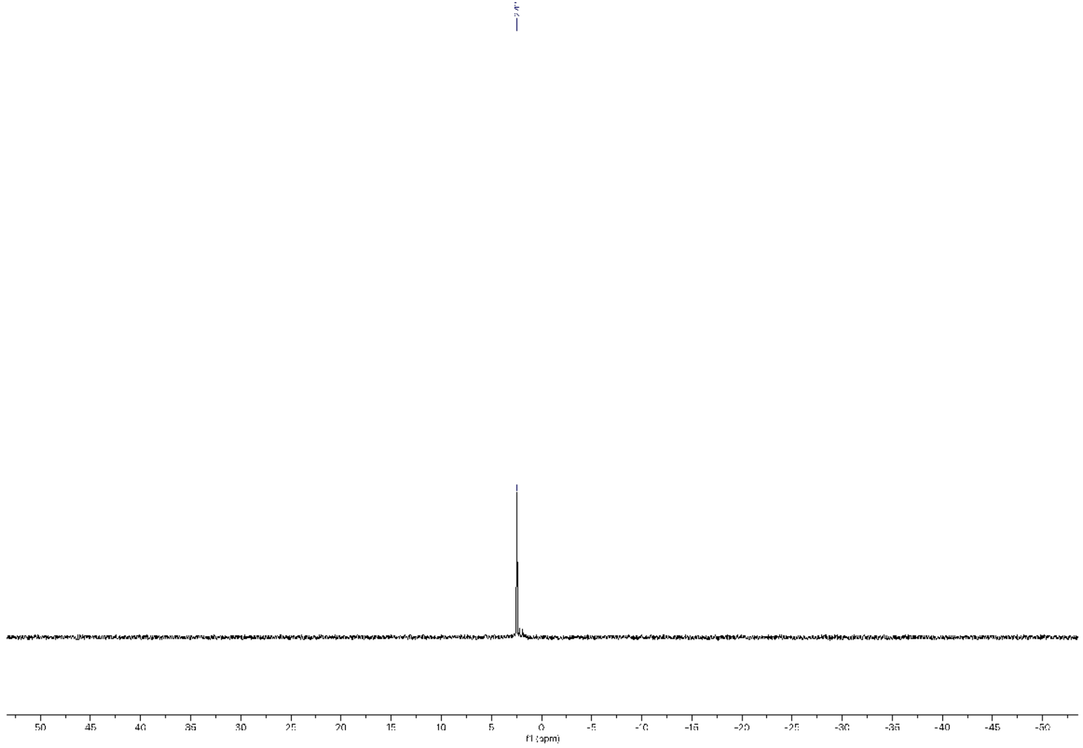

Fig. S38 ^{31}P NMR spectrum of dihydrouridine-3'-phosphate.

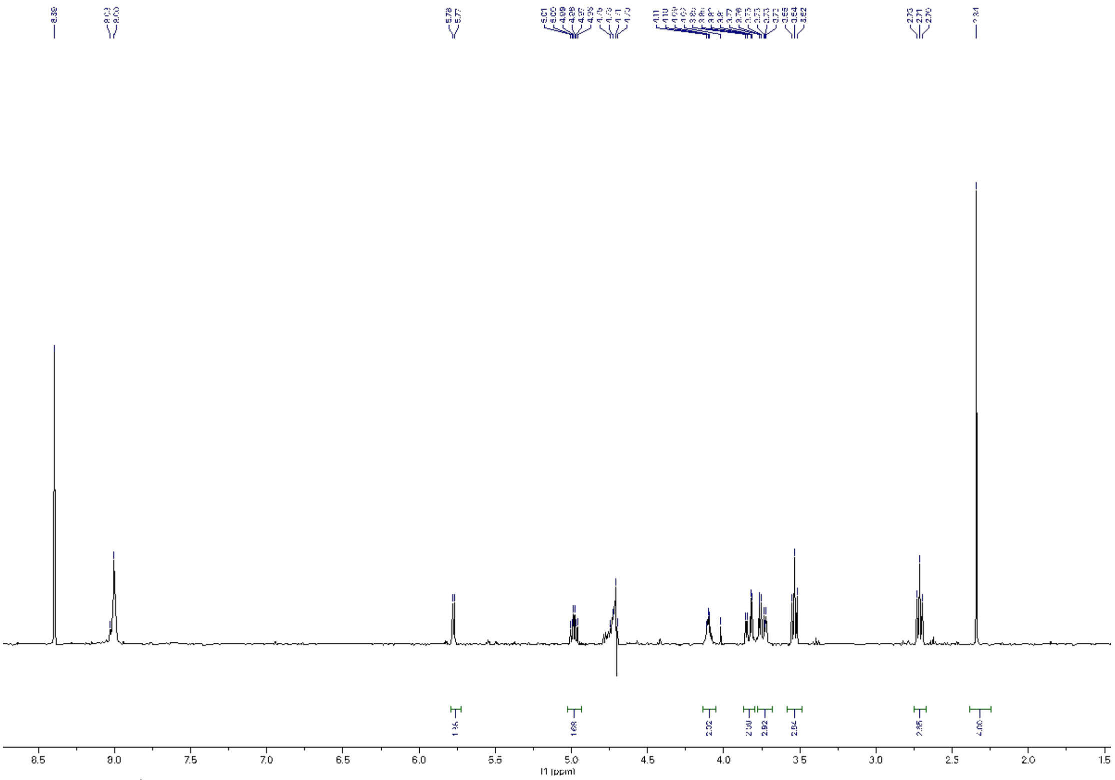

Fig. S39 ^{1}H NMR spectrum of the mixture after photoreaction of uridine-2',3'-phosphate in formamide with added sodium succinate (60 uL of 50 mM solution).

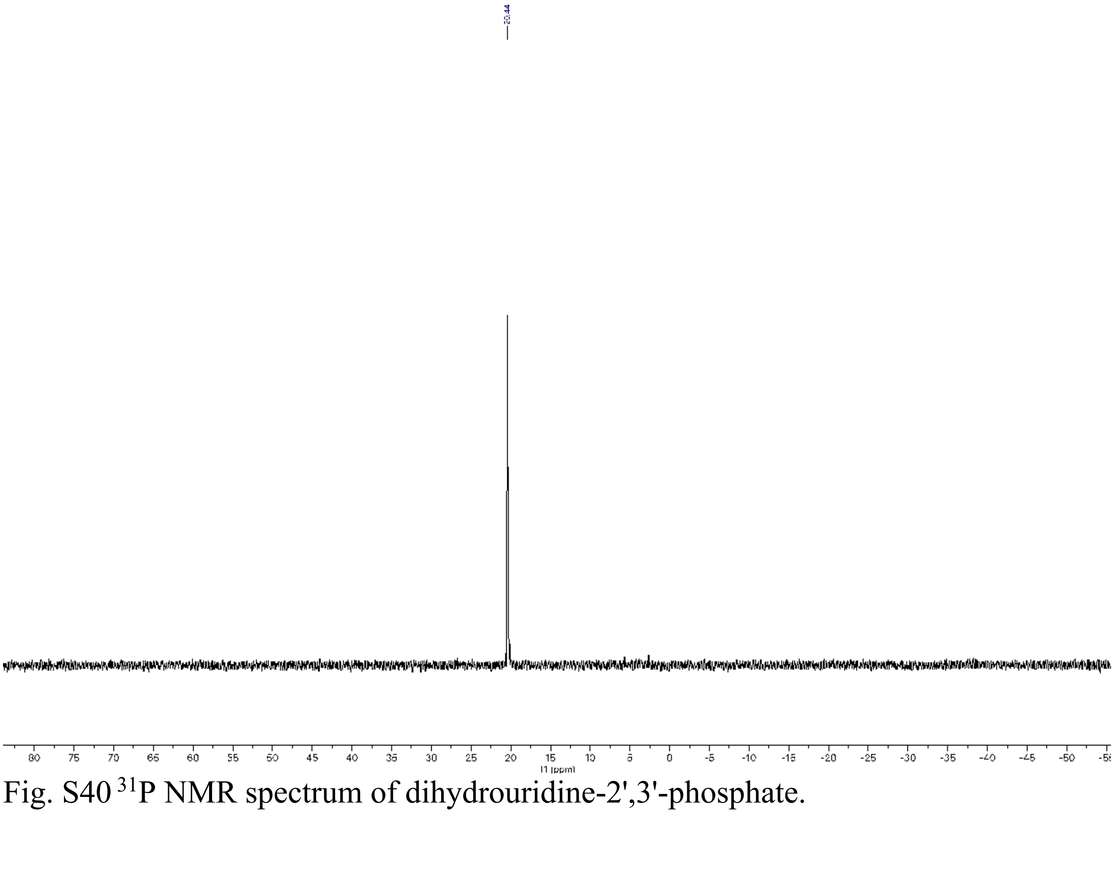

Fig. S40 ^{31}P NMR spectrum of dihydrouridine-2',3'-phosphate.

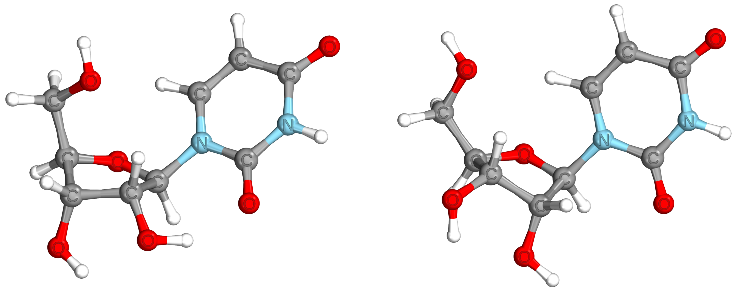

C2'-endo Uridine C3'-endo Uridine

Fig. S41 - The optimized ground-state structures of uridine having the C2'-endo and C3'-endo arrangement of the ribose fragment at the PCM(formamide)/ωB97X-D/def2-TZVP level of theory.

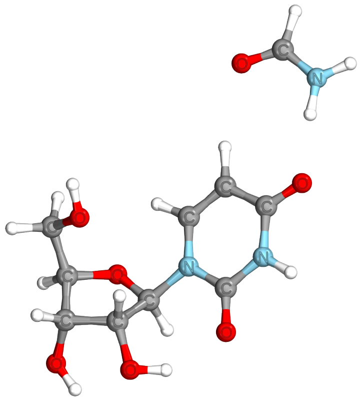

Fig. S42 - The equilibrium S_0 geometry of the C2'-endo uridine and formamide complex is shown, and the explicit solvent formamide molecule was placed at the C4=O carbonyl group. The presented structure was found at the PCM(formamide)/ωB97X-D/def2-TZVP level of theory.

State / Transition		E_{exc} [eV]	f_{osc}	λ [nm]
**Uridine-formamide complex**				
S_1	$\pi\pi^*$	5.160	0.299	240.3
S_2	$n_O\pi^*$	5.236	0.015	236.8
S_3	$n_O\pi^*$	5.656	0.001	219.2

Table S1 - Vertical excitation energies (in eV) of the uridine-formamide complex were computed using the SCS-ADC(2)/aug-cc-pVDZ level of theory.

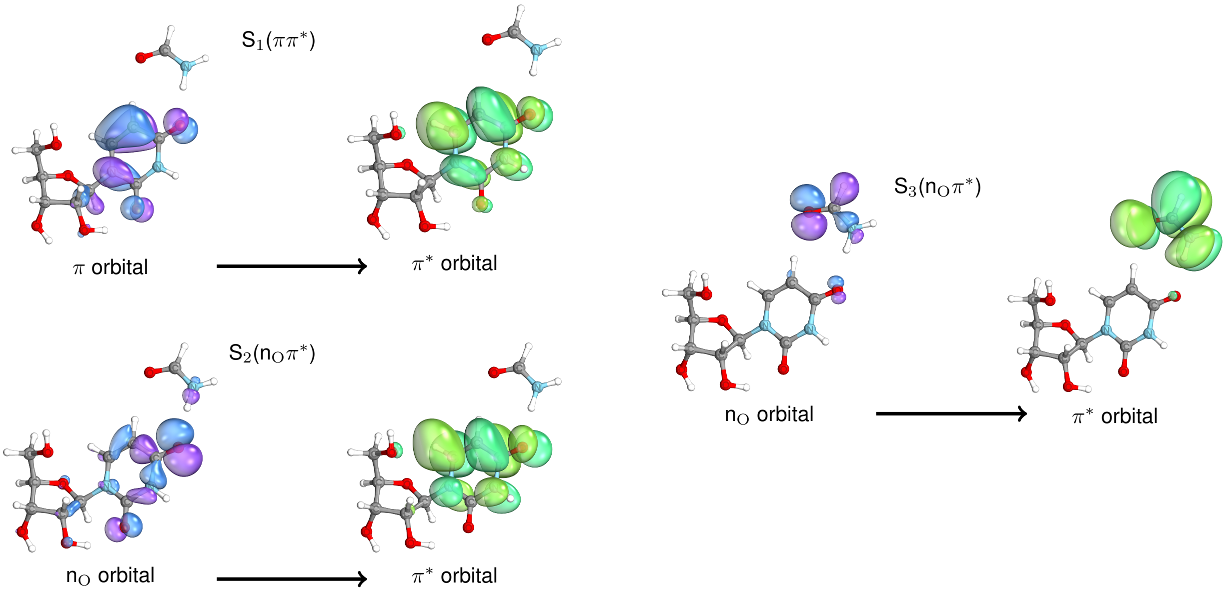

Fig. S43 – The natural transition orbitals involved in electronic transitions of the lowest-lying three excited states for the complex of uridine and formamide molecules, assuming the equilibrium ground-state structure.

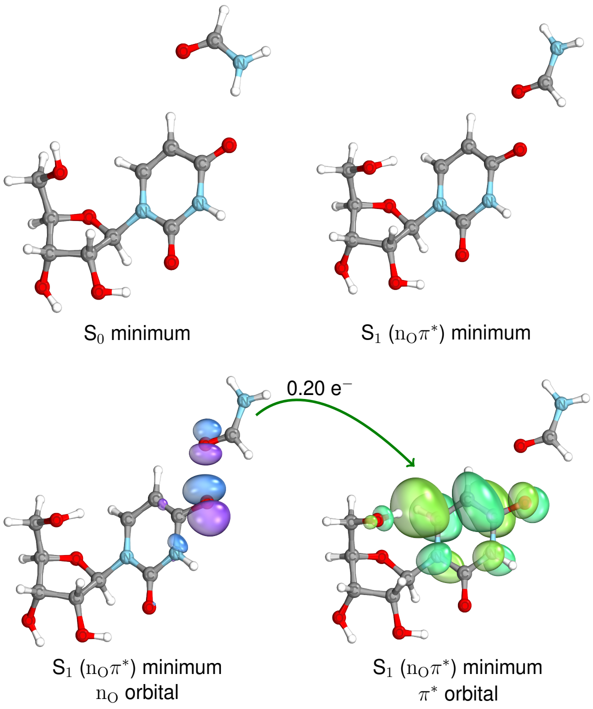

Fig. S44 – In the first row, there are shown the optimized S_0 and S_1 structures obtained using the PCM/ωB97X-D/def2-TZVP and SCS-ADC(2)/aug-cc-pVDZ methods, respectively. Below are depicted natural transition orbitals, generated for the S_1 -minimum-energy geometry, which participate in the $n_O\pi^*$ transition. The wavefunction analysis revealed an intermolecular charge transfer of 0.20 e^- from the formamide n_O orbital to the uridine π^* molecular orbital in the S_1 state.

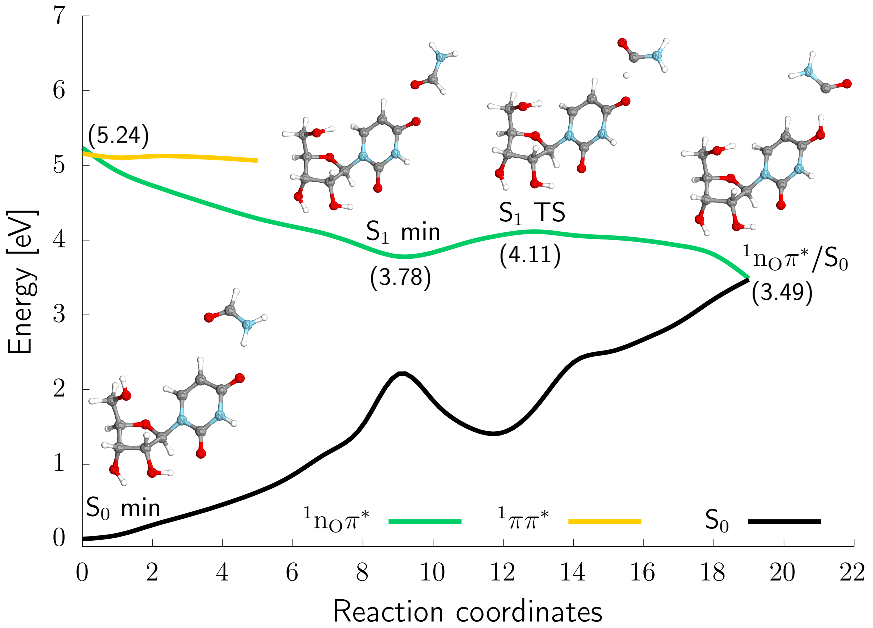

Fig. S45 – The non-radiative photorelaxation pathway demonstrates a high-energy chemical reaction that starts from the Franck-Codon region (5.24 eV), then the system reaches the S_1 minimum at 3.78 eV, and subsequently, there is the S_1 transition-state geometry (4.11 eV) enabling for the hydrogen atom transfer from formamide to the uridine carbonyl oxygen atom. The excited-state intermolecular hydrogen transfer leads to the biradical system, having hydrouridine and formamide radicals, and the formation of the S_1/S_0 minimum-energy crossing point (3.49), allowing for the deactivation to the electronic ground state. The presented photochemical mechanism possesses only one energy barrier between the S_1 minimum and S_1 transition-state structure, equal to 0.33 eV. The orange and green lines correspond to the $^1\pi\pi^*$ and $^1n_O\pi^*$ excited states, respectively. The black line shows the electronic closed-shell ground state. The ground and excited states potential energy surfaces were obtained at the SCS-MP2/aug-cc-pVDZ and SCS-ADC(2)/aug-cc-pVDZ level of theories, respectively.

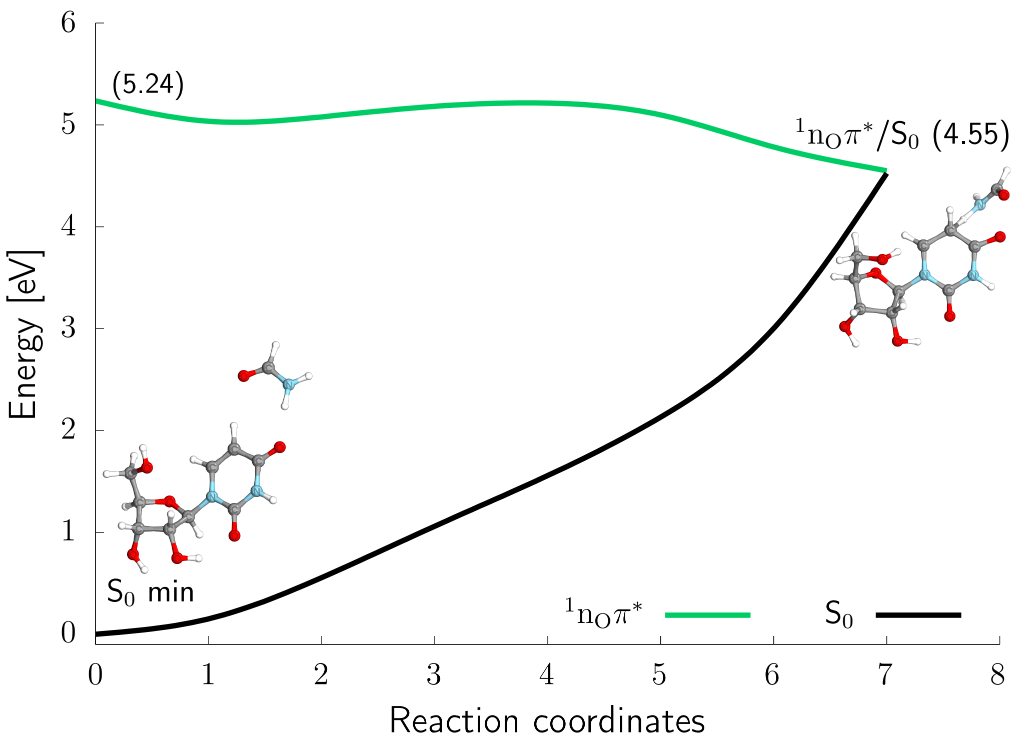

Fig. S46 – The radiationless photorelaxation channel shows a high-energy chemical reaction that initiates from the Franck-Codon region (5.24 eV). Then, the model system goes towards the $S_1(n_O\pi^*)/S_0$ conical intersection seam at 4.55 eV, which corresponds to the $S_1(n_O\pi^*)$ transition-state excited-state geometry. The photochemical reaction enables the hydrogen atom transfer from formamide to the uridine C5 atom in the concerted proton-coupled electron transfer manner (PCET). The concerted PCET results in forming the biradical system composed of hydrouridine and formamide radicals. Reaching the intersection seam (at 4.55 eV) allows for the deactivation to the electronic ground state. The green line corresponds to the $^1n_O\pi^*$ excited state. The black line depicts the electronic closed-shell ground state. The ground and excited state potential energy surfaces were obtained at the SCS-MP2/aug-cc-pVDZ and SCS-ADC(2)/aug-cc-pVDZ level of theories, respectively.

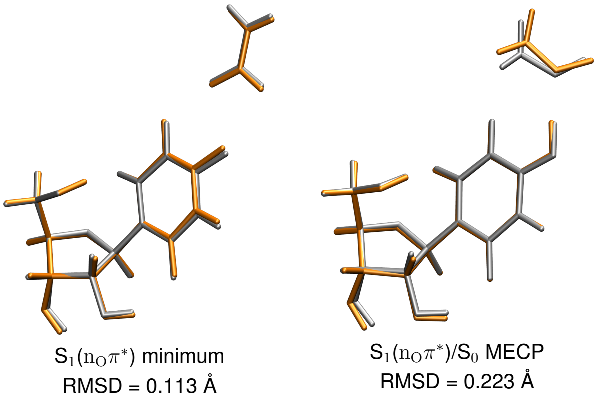

Fig. S47 – The silver and orange superimposed minimum-energy geometries obtained at the XMS-CASPT2/SA-3-CASSCF(10,9)/cc-pVDZ and SCS-ADC(2)/aug-cc-pVDZ level of theories, respectively.

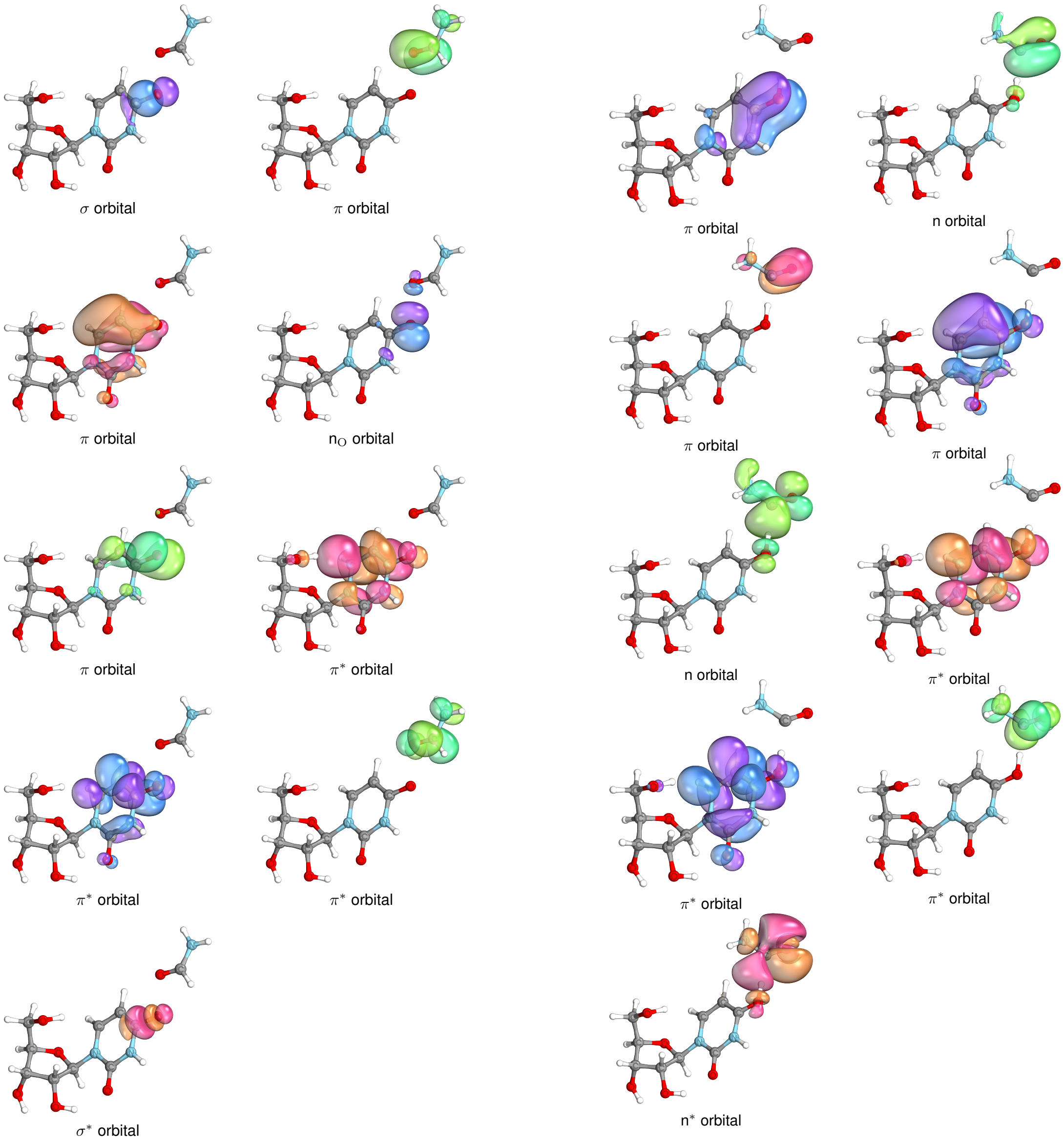

Fig. S48 – The left and right panels show the molecular orbitals which were selected to build an active space in the XMS-CASPT2/SA-3-CASSCF(10,9)/cc-pVDZ optimization for the S_1 and S_1/S_0 minimum-energy structures, respectively.

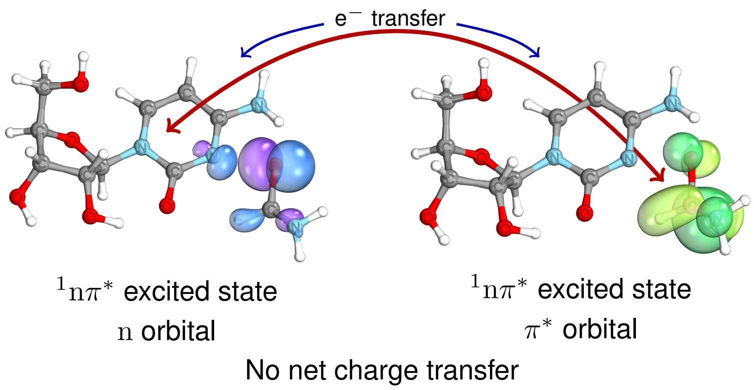

Fig. S49 – The S_1(nπ*) minimum-energy structure of the cytidine and formamide complex obtained at the SCS-ADC(2)/aug-cc-pVDZ level of theory. The shown natural transition orbitals represent the occupied n and virtual π* molecular orbitals involved in the S_1 state. The wavefunction analysis of the structure demonstrated that there is no net charge transfer between the molecules.

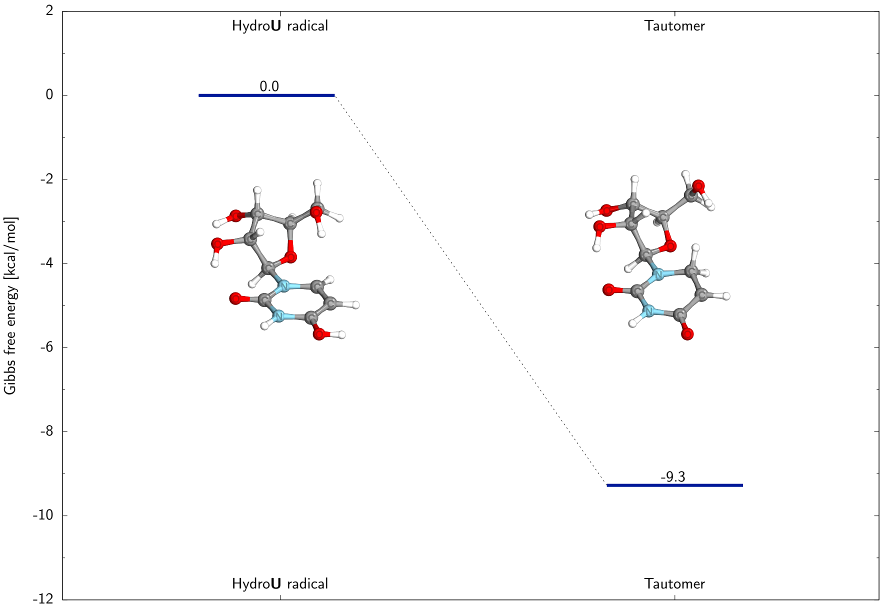

Fig. S50 – The Gibbs free energy profiles present two various tautomers of the hydrouridine radical having either a proton at the C4-O (0.0 kcal/mol) or C6 position (-9.3 kcal/mol), obtained at the PCM/ωB97X-D/def2-TZVP level.

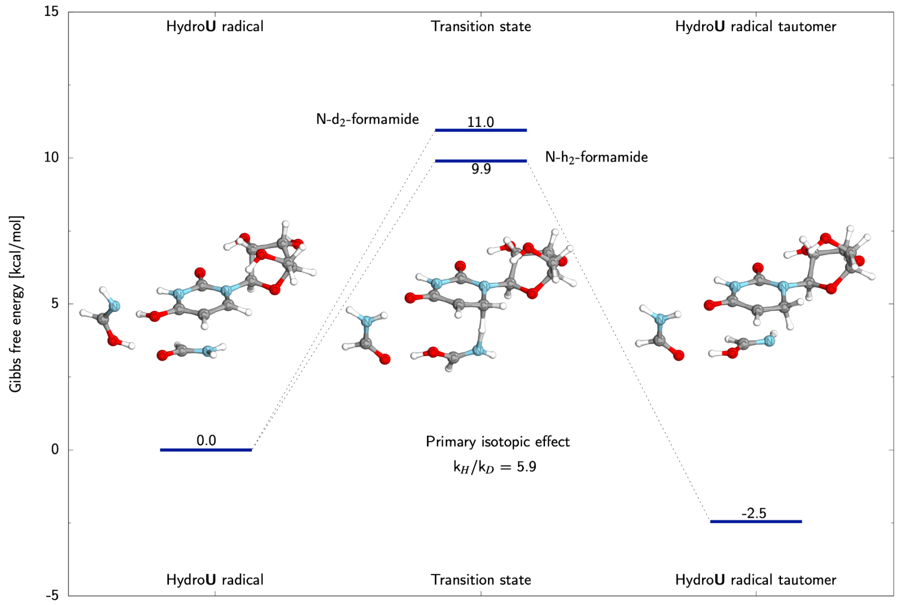

Fig. S51 – The Gibbs free energy profiles, obtained at the PCM/ωB97X-D/def2-TZVP level, show a proton transfer from the C4-O to the C6 site of the hydrouridine (U) radical through two formamide molecules, one in the enol and the other keto form, leading to more stable radical. The presented proton relay transition state was computed for N-h_2-formamide or N-d_2-formamide molecules to estimate the primary isotopic effect of the proton relay mechanism, that is $k_H/k_D = 5.9$.

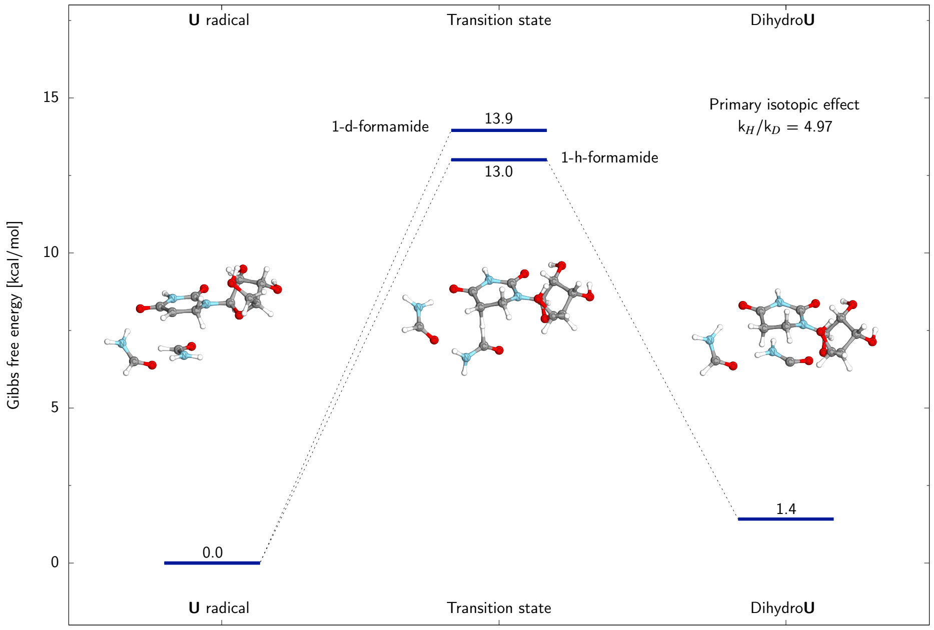

Fig. S52 – The Gibbs free energy profiles, obtained at the PCM/ωB97X-D/def2-TZVP level, demonstrate a hydrogen atom abstraction mechanism in which either 1-h-formamide or 1-d-formamide molecule donates the hydrogen atom to the C5 position of the nucleoside resulting in the formation of dihydrouridine and formamide radical. The presented transition state was computed for 1-h-formamide or 1-d-formamide molecules to evaluate the primary isotopic effect of the hydrogen atom abstraction process, that is $k_H/k_D = 4.97$.

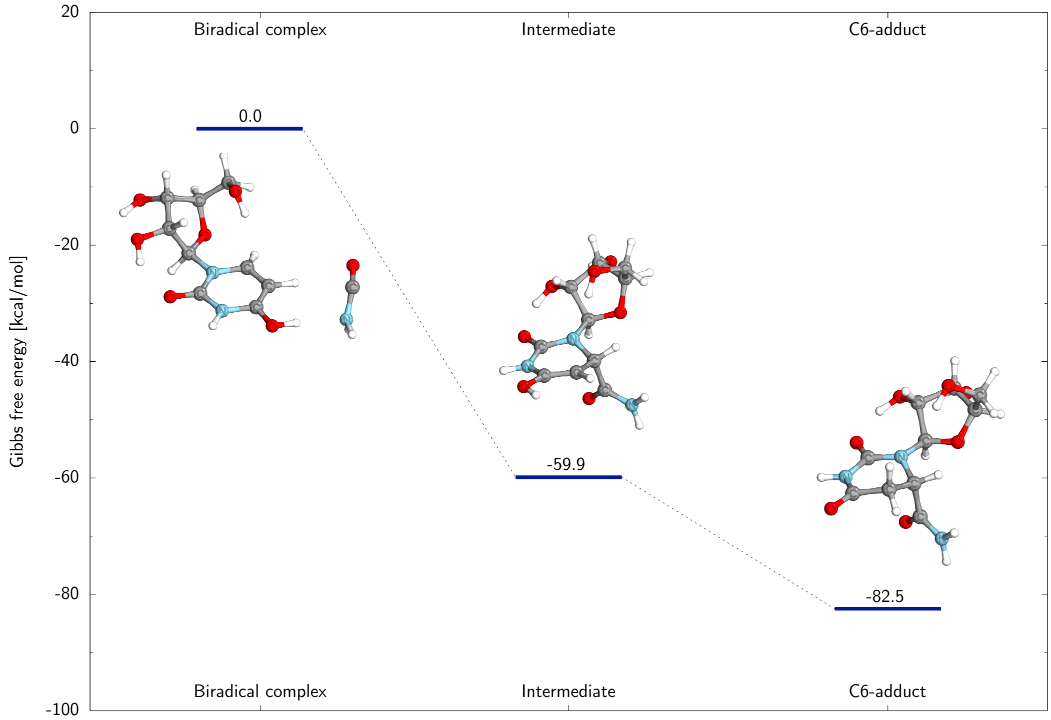

Fig. S53 – The Gibbs free energy profiles present the formation of the C6-adduct of the hydrouridine and formamide radicals, computed at the PCM/ωB97X-D/def2-TZVP level of theory.

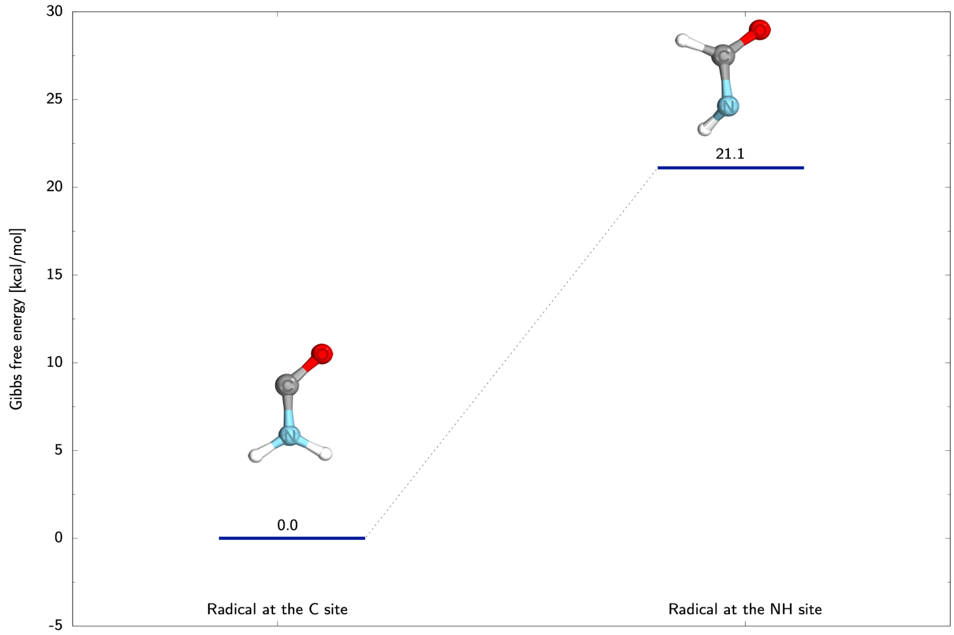

Fig. S54 – The Gibbs free energy profiles present the stability of formamide radicals obtained at the PCM/ωB97X-D/def2-TZVP level of theory.

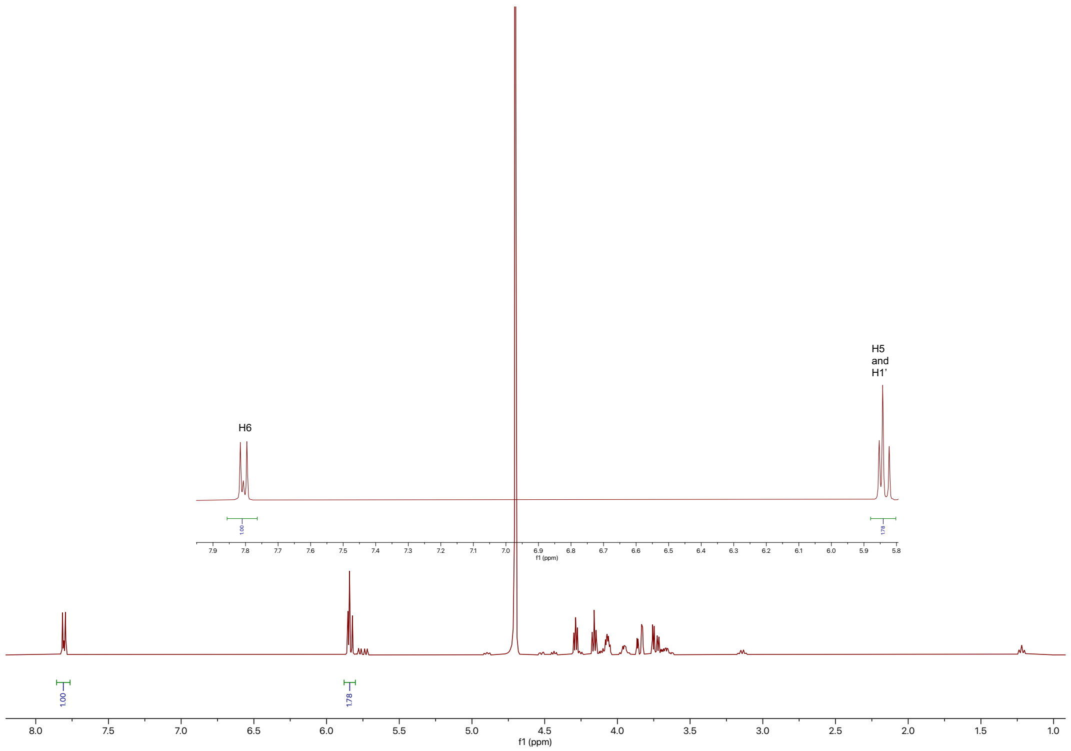

Figure S55. 5-*d*-uridine **29** was coeluted with uridine in HPLC and recorded in ^{1}H-NMR after 4 hour's irradiations of uridine in *N*-d_2-formamide (60 mM) at 254 nm.

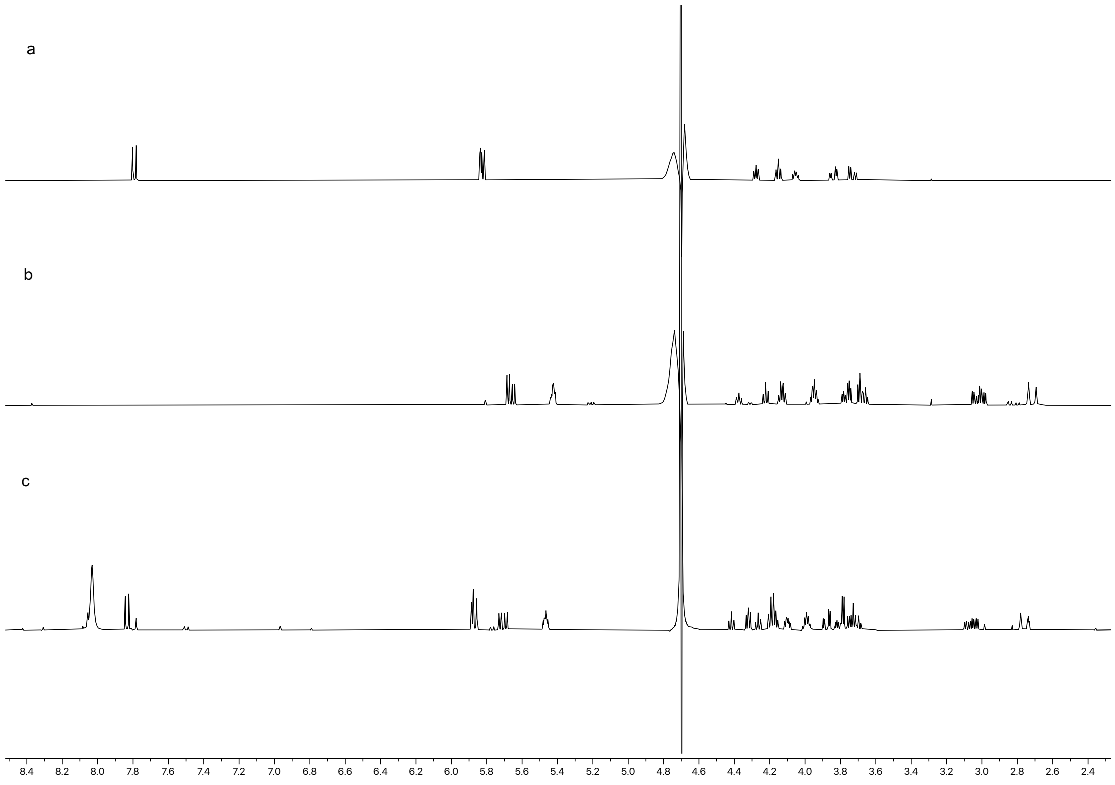

Figure S56. Uridine **2** was reformed by dehydration of uridine hydrate during evaporation of formamide at 60 °C under high vacuum. a) ^{1}H NMR of uridine **2** standard; b) ^{1}H NMR of uridine hydrate **4** formed from photoreaction of uridine in H_2O; c) ^{1}H NMR of the mixture after removing formamide from **4** formamide solution by evaporation.

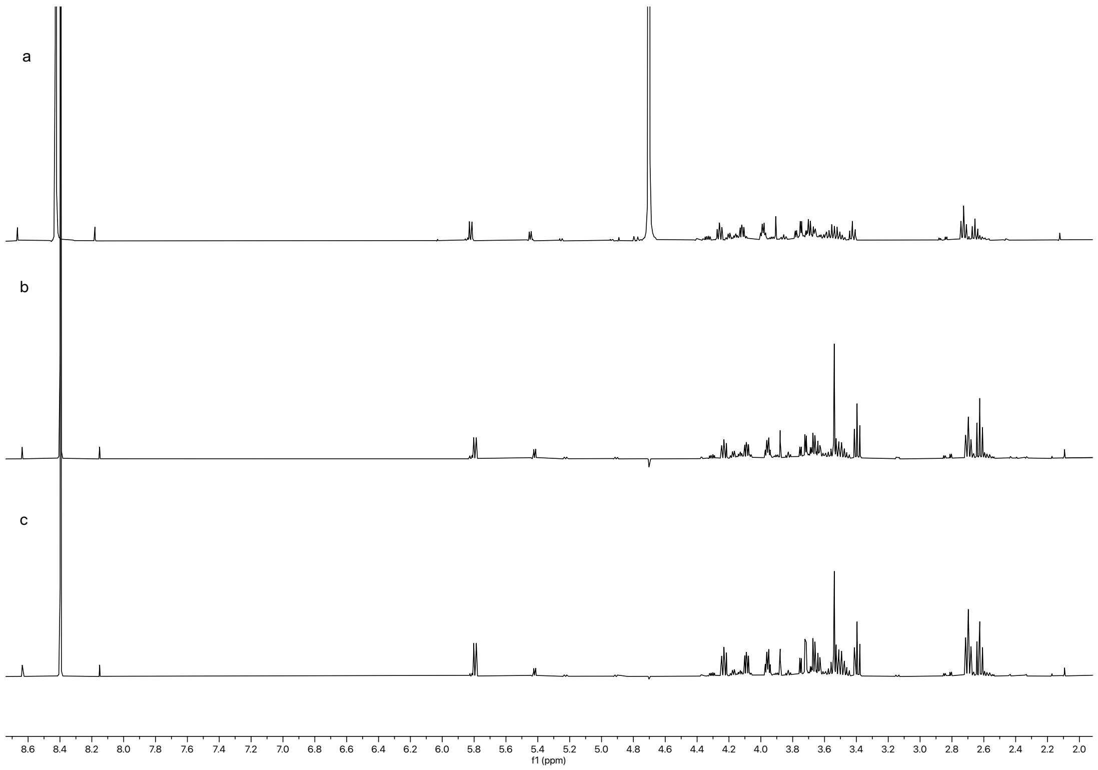

Figure S57. Dihydrouridine was formed (in 50% yield) by photoreaction of uridine in water in the presence of sodium formate (12 equivalents). a) ^{1}H NMR of the mixture after 2 hour's irradiations; b) as a), spiked with dihydrouracil; c) as b), spiked with dihydrouridine.

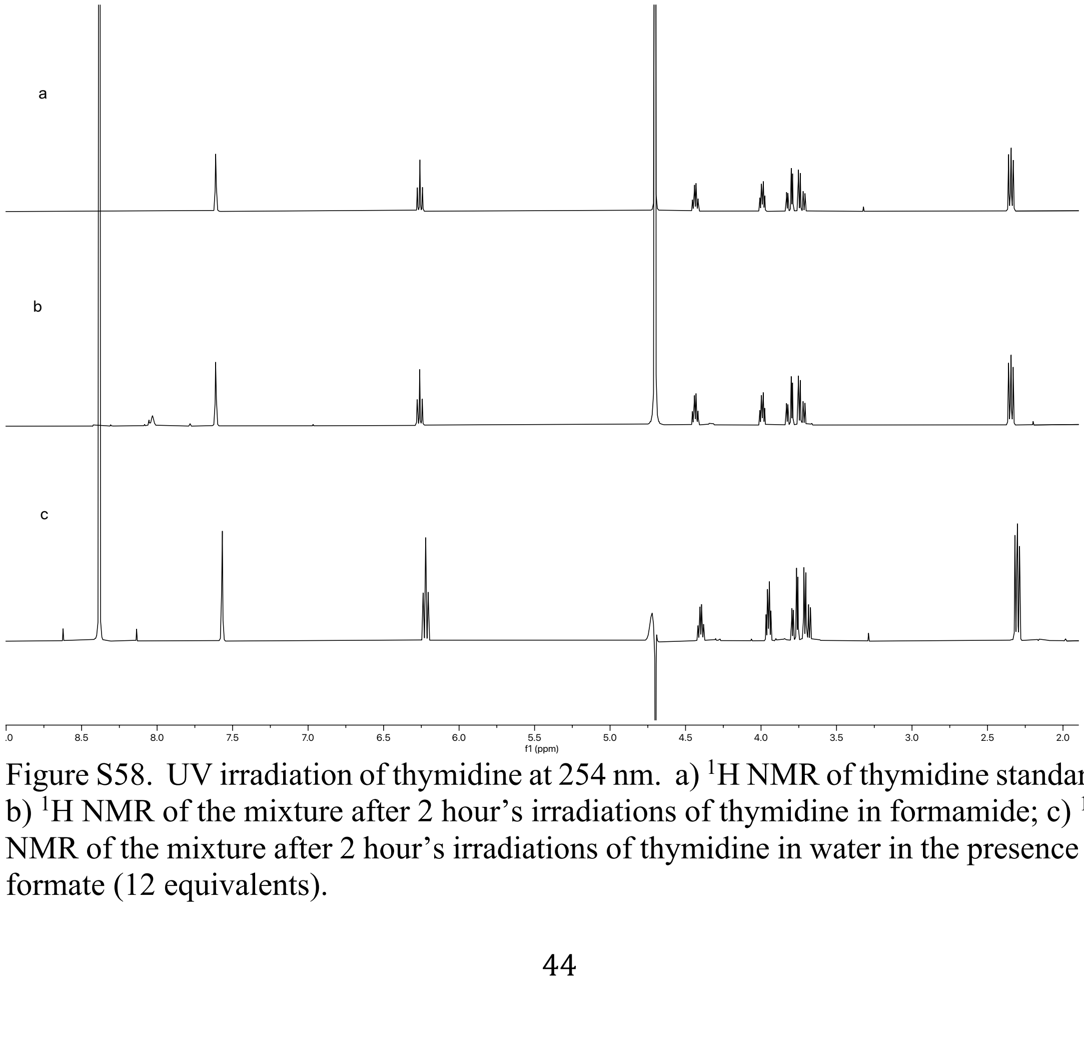

Figure S58. UV irradiation of thymidine at 254 nm. a) ^{1}H NMR of thymidine standard; b) ^{1}H NMR of the mixture after 2 hour's irradiations of thymidine in formamide; c) ^{1}H NMR of the mixture after 2 hour's irradiations of thymidine in water in the presence of formate (12 equivalents).

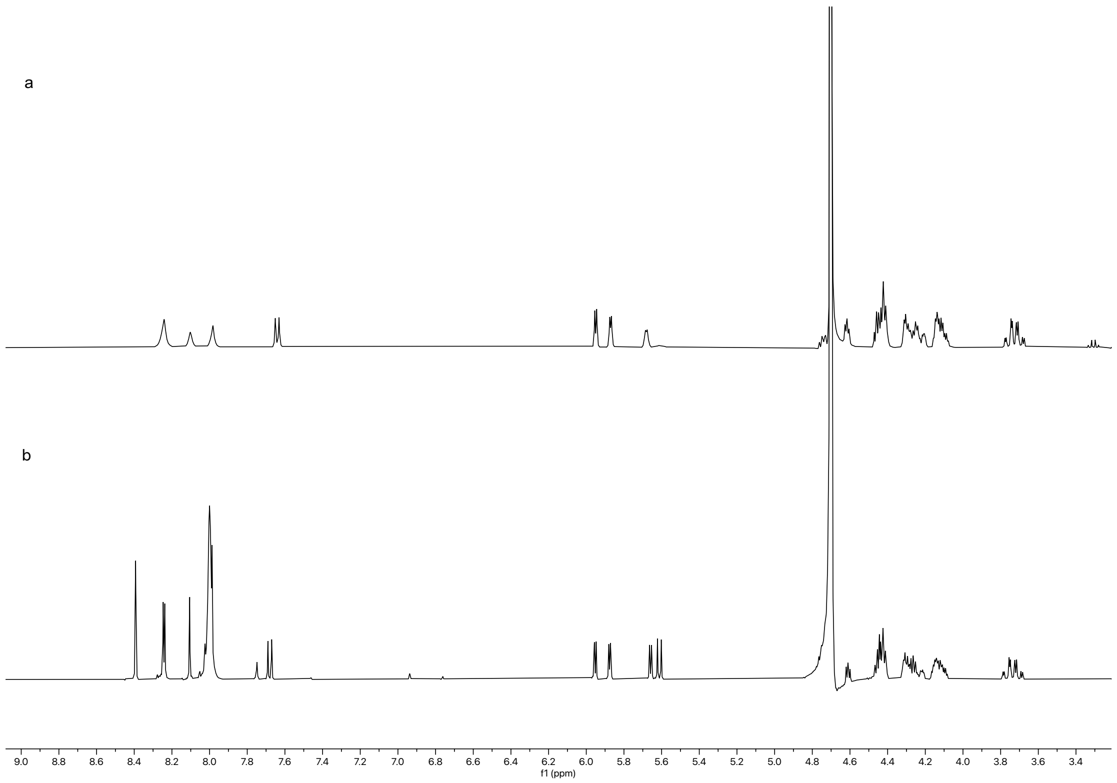

Figure S59. UV irradiation of trimer UAA at 254 nm. a) ^{1}H NMR of UAA before irradiation; b) ^{1}H NMR of the mixture after 2 hour's irradiations of UAA in formamide.

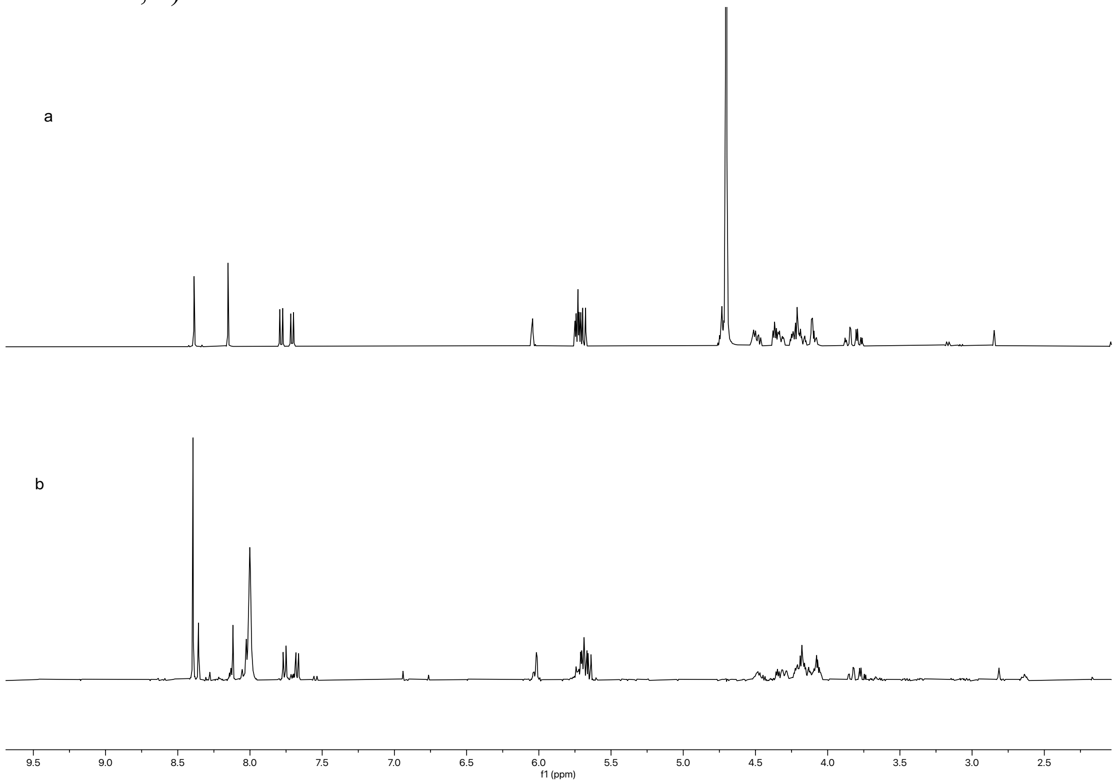

Figure S60. UV irradiation of trimer UAC at 254 nm. a) ^{1}H NMR of UAC before irradiation; b) ^{1}H NMR of the mixture after 2 hour's irradiations of UAC in formamide.

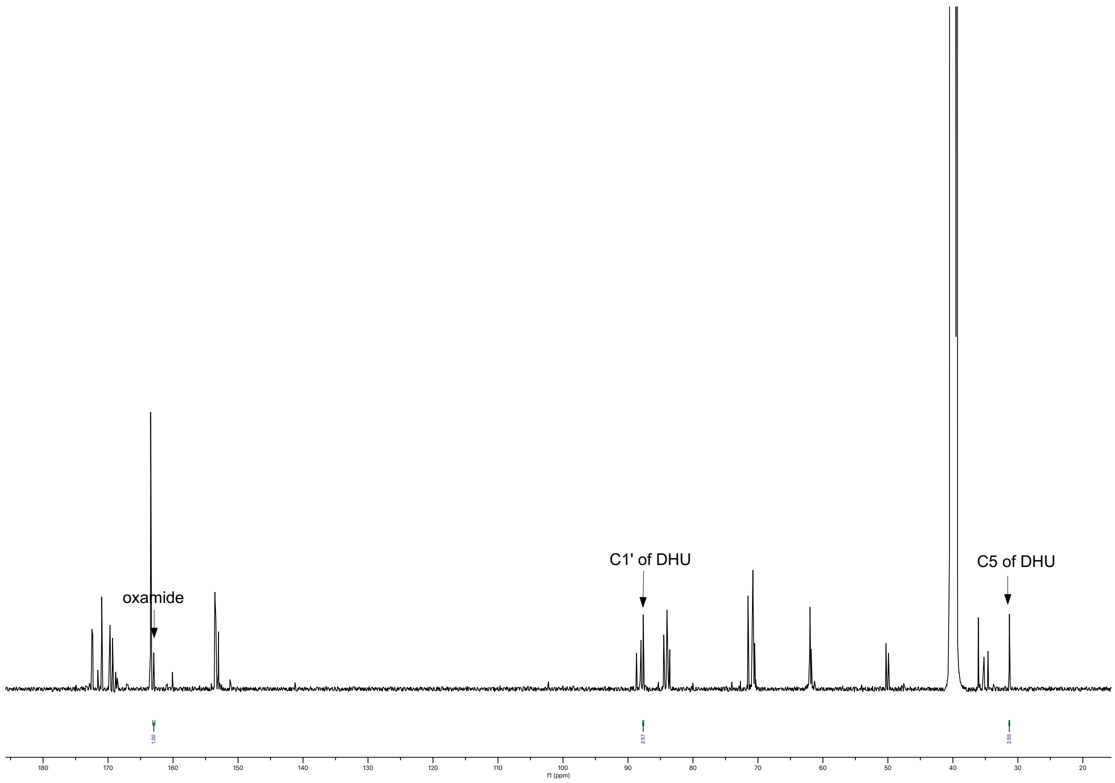

Figure S61. Quantitative ^{13}C NMR spectrum of the mixture after irradiation of 60 mM of uridine in formamide. (The yield of oxamide (20%) was calculated by using DHU (54%) as internal standard.)

References:

(1) P. Pracht, F. Bohle, S. Grimme, *Phys. Chem. Chem. Phys.* 2020, **22 (14)**, 7169–7192.
(2) S. Grimme, *J. Chem. Theory Comput.* 2019, **15 (5)**, 2847–2862.
(3) J.-D. Chai, M. Head-Gordon, *Phys. Chem. Chem. Phys.* 2008, **10 (44)**, 6615.
(4) F. Weigend, R. Ahlrichs, *Phys. Chem. Chem. Phys.* 2005, **7 (18)**, 3297.
(5) E. Cancès, B. Mennucci, J. Tomasi, *J. Chem. Phys.* 1997, **107 (8)**, 3032–3041.
(6) G. Scalmani, M. J. Frisch, *J. Chem. Phys.* 2010, **132 (11)**, 114110.
(7) M. J. Frisch, G. W. Trucks, H. B. Schlegel, G. E. Scuseria, M. A. Robb, J. R. Cheeseman, G. Scalmani, V. Barone, G. A. Petersson, H. Nakatsuji, X. Li, M. Caricato, A. V. Marenich, J. Bloino, B. G. Janesko, R. Gomperts, B. Mennucci, H. P. Hratchian, J. V. Ortiz, A. F. Izmaylov, J. L. Sonnenberg, D. Williams-Young, F. Ding, F. Lipparini, F. Egidi, J. Goings, B. Peng, A. Petrone, T. Henderson, D. Ranasinghe, V. G. Zakrzewski, J. Gao, N. Rega, G. Zheng, W. Liang, M. Hada, M. Ehara, K. Toyota, R. Fukuda, J. Hasegawa, M. Ishida, T. Nakajima, Y. Honda, O. Kitao, H. Nakai, T. Vreven, K. Throssell, Jr. J. A. Montgomery, J. E. Peralta, F. Ogliaro, M. J. Bearpark, J. J. Heyd, E. N. Brothers, K. N. Kudin, V. N. Staroverov, T. A. Keith, R. Kobayashi, J. Normand, K. Raghavachari, A. P. Rendell, J. C. Burant, S. S. Iyengar, J. Tomasi, M. Cossi, J. M. Millam, M. Klene, C. Adamo, R. Cammi, J. W. Ochterski, R. L. Martin, K. Morokuma, O. Farkas, J. B. Foresman, D. J. Fox, Gaussian˜16 Revision C.01. 2016.
(8) A. Hellweg, S. A. Grün, C. Hättig, *Phys. Chem. Chem. Phys.* 2008, **10 (28)**, 4119.
(9) C. Hättig, *Adv. Quantum Chem.* 2005, 37–60.

(10) A. Dreuw, M. Wormit, *Wiley Interdiscip. Rev. Comput. Mol. Sci.* 2015, **5 (1)**, 82–95.
(11) R. A. Kendall, T. H. Dunning, R. J. Harrison, *J. Chem. Phys*. 1992, **96 (9)**, 6796–6806.
(12) F. Plasser, *J. Chem. Phys.* 2020, **152 (8)**, 084108.
(13) B. G. Levine, J. D. Coe, T. J. Martínez, *J. Phys. Chem. B* 2008, **112 (2)**, 405–413.
(14) TURBOMOLE V7.3 2018, a Development of University of Karlsruhe and Forschungszentrum Karlsruhe GmbH, 1989-2007, TURBOMOLE GmbH, since 2007; Available from http://www.turbomole.com.
(15) S. Smidstrup, A. Pedersen, K. Stokbro, H. Jónsson, *J. Chem. Phys.* 2014, **140 (21)**, 214106.
(16) P. Plessow, *J. Chem. Theory Comput.* 2013, **9 (3)**, 1305–1310.
(17) Carlos. Gonzalez, H. Bernhard. Schlegel, *J. Chem. Phys.* 1990, **94 (14)**, 5523–5527.
(18) F. Neese, F. Wennmohs, U. Becker, C. Riplinger, *J. Chem. Phys.* 2020, **152 (22)**, 224108.
(19) K. Andersson, P. Malmqvist, B. O. Roos, *J. Chem. Phys*. 1992, **96 (2)**, 1218–1226.
(20) Kerstin. Andersson, P. Aake. Malmqvist, B. O. Roos, A. J. Sadlej, Krzysztof. Wolinski, *J. Phys. Chem.* 1990, **94 (14)**, 5483–5488.
(21) V. Veryazov, P. Å. Malmqvist, B. O. Roos, *Int. J. Quantum Chem*. 2011, **111 (13)**, 3329–3338.
(22) T. Shiozaki, *Wiley Interdiscip. Rev. Comput. Mol. Sci.* 2018, **8 (1)**, https://doi.org/10.1002/wcms.1331.
(23) R. Szabla, H. Kruse, J. Šponer, R. W. Góra, *Phys. Chem. Chem. Phys.* 2017, **19 (27)**, 17531–17537.
(24) M. J. Janicki, R. Szabla, J. Šponer, R. W. Góra, *Phys. Chem. Chem. Phys.* 2022. https://doi.org/10.1039/D2CP00801G.
(25) E. Marsili, A. Prlj, B. F. E. Curchod, *Phys. Chem. Chem. Phys.* 2021, **23 (23)**, 12945–12949.
(26) Y. Miura, Y. Yamamoto, S. Karashima, N. Orimo, A. Hara, K. Fukuoka, T. Ishiyama, T. Suzuki, *J. Am. Chem. Soc.* 2023, **145 (6)**, 3369–3381.
(27) M. Barbatti, *J. Am. Chem. Soc.* 2014, **136 (29)**, 10246–10249.
(28) R. F. Fink, *J. Chem. Phys.* 2010, **133 (17)**, 174113.
(29) J. Richardi, H. Krienke, P. H. Fries, *Chem. Phys. Lett.* 1997, **273 (3–4)**, 115–121.
